# Interrogating 1000 insect genomes for NUMTs: A risk assessment for estimates of species richness

**DOI:** 10.1371/journal.pone.0286620

**Published:** 2023-06-08

**Authors:** Paul D. N. Hebert, Dan G. Bock, Sean W. J. Prosser

**Affiliations:** Centre for Biodiversity Genomics, University of Guelph, Guelph, ON, Canada; University of Veterinary Medicine Vienna: Veterinarmedizinische Universitat Wien, AUSTRIA

## Abstract

The nuclear genomes of most animal species include NUMTs, segments of the mitogenome incorporated into their chromosomes. Although NUMT counts are known to vary greatly among species, there has been no comprehensive study of their frequency/attributes in the most diverse group of terrestrial organisms, insects. This study examines NUMTs derived from a 658 bp 5’ segment of the cytochrome *c* oxidase I (COI) gene, the barcode region for the animal kingdom. This assessment is important because unrecognized NUMTs can elevate estimates of species richness obtained through DNA barcoding and derived approaches (eDNA, metabarcoding). This investigation detected nearly 10,000 COI NUMTs ≥ 100 bp in the genomes of 1,002 insect species (range = 0–443). Variation in nuclear genome size explained 56% of the mitogenome-wide variation in NUMT counts. Although insect orders with the largest genome sizes possessed the highest NUMT counts, there was considerable variation among their component lineages. Two thirds of COI NUMTs possessed an IPSC (indel and/or premature stop codon) allowing their recognition and exclusion from downstream analyses. The remainder can elevate species richness as they showed 10.1% mean divergence from their mitochondrial homologue. The extent of exposure to “ghost species” is strongly impacted by the target amplicon’s length. NUMTs can raise apparent species richness by up to 22% when a 658 bp COI amplicon is examined versus a doubling of apparent richness when 150 bp amplicons are targeted. Given these impacts, metabarcoding and eDNA studies should target the longest possible amplicons while also avoiding use of 12S/16S rDNA as they triple NUMT exposure because IPSC screens cannot be employed.

## Introduction

The nuclear genomes of most animal species contain segments of the mitogenome [1] captured during the repair of double-strand breaks associated with meiotic recombination [2, 3]. Many of these NUMTs (nuclear DNA sequences of mitochondrial origin) are short, but some include much of the mitochondrial genome [4]. Their prevalence reflects both recurrent integration events and subsequent duplication and diversification. For example, more than 750 NUMTs, ranging in length from 100 bp to 16,106 bp, comprise 0.01% of the human genome [4, 5]. A third of them have arisen through distinct insertion events; the rest likely reflect duplications following integration [6–8]. Extensive variation is apparent in their age; some entered the nuclear genome tens of millions of years ago while others are recent [5, 9]. While mechanisms of NUMT insertion are not fully characterized [10], their incorporation seems to follow the entanglement of mtDNA with nDNA during cell division [11] as densities are highest near centromeres [12, 13]. Although most NUMTs are not transcribed, some appear to regulate gene activity [14] while others impact the phenotype by disrupting gene function; such cases are, for example, responsible for certain human diseases [10]. While NUMTs can offer novel phylogenetic insights [15] because sequence change is slowed 3x–4x after their transfer into the nuclear genome [16], they also represent a complication for identification systems that employ mitochondrial markers for species discrimination [17–19].

NUMT counts differ markedly among animal lineages and are positively correlated with size of the nuclear genome [10], but they do vary among closely related taxa. For example, species of *Apis* (honeybee) have many more NUMTs than most other members of their family [8]. In taxa with multiple NUMTs, sequence divergence from mtCOI often shows considerable variation reflecting their different timing of incorporation. Those with > 2% sequence divergence pose complexity to approaches using mitochondrial markers for species identification, such as the COI region employed for DNA barcoding [20]. While NUMTs with an IPSC (indel or premature stop codon) can be identified and filtered, those lacking these features are readily mistaken for the target mitochondrial marker, inflating estimates of diversity in contexts ranging from studies of dietary composition [21] to species richness [17]. To evaluate their impact on such applications, we utilized public nuclear and mitochondrial sequence data to examine the prevalence of COI-derived NUMTs in 1,002 insect species. Among these taxa, 668 possessed a nuclear assembly derived from high coverage data making it possible to estimate genome size, and, hence, to examine the relationship between genome size and NUMT abundance/attributes. Analysis of this dataset also allowed evaluation of their impacts on the varied analytical approaches that employ mitochondrial markers, especially COI, for biodiversity assessments.

## Materials and methods

### Nuclear genome dataset

Analysis began with extraction of metadata for the 1,478 insect species with nuclear genome assemblies in NCBI’s Genome database (https://www.ncbi.nlm.nih.gov/genome/) in late 2021 using the assembly-stats option of the ‘ncbi-genome-download’ package (https://github.com/kblin/ncbi-genome-download; see also **S1 Fig** for an overview of the data analysis pipeline). Sequence coverage, contig N50, and assembly level (i.e., contig, scaffold, chromosome) were recorded and this information was used to select a representative assembly for each species when several were available. Specifically, we favoured chromosome over scaffold over contig assemblies. When a species had multiple genomes with the same assembly level, we chose the one with the highest coverage. We next used ‘taxize’ R [22] to record the membership of each species in an insect order and family. Thirty-two of the 1,478 assemblies were subsequently excluded because of data problems: 15 derived from bacterial endosymbionts (see S1 File–Nuclear genome sizes), 13 lacked a species identification, 2 were hybrids, and 2 others (*Nasutitermes exitiosus*, *Xenocatantops brachycerus*) had such small nuclear assemblies that they appear unlikely to be valid. The remaining 1,446 assemblies were downloaded between 11/29/21–12/2/21 using ‘ncbi-genome-download’.

### COI barcode dataset from BOLD

In order to screen nuclear genomic sequences for the presence of COI-derived NUMTs, a COI query sequence is required. Ideally, this sequence would originate from the specimen used to obtain the nuclear assembly, but this was not always possible due to either the absence of mitogenomic data, or to the fact that many insect nuclear genomes have been assembled from multiple individuals (e.g. [23, 24]). In such cases, a closely related COI query sequence can suffice to detect most if not all NUMTs in a species (pers. obs., data not shown). To this end, we searched BOLD for COI-5’ sequences > 645 bp from the 1,446 species and downloaded all those meeting this criterion [25]. For sequences > 665 bp, the barcode region was excised using Aliview [26]. If more than one Barcode Index Number (BIN; [27]) was associated with a binomen (as expected for unrecognized species complexes), the dominant BIN was used. Those flagged as contaminants, those with stop codons, and those marked as problematic were omitted. After applying these filters, COI barcodes were recovered from 783 (54.1%) of the 1,446 species.

### Mitogenome dataset

We searched for mitogenomes from the 1,446 species to provide additional COI barcodes and to make it possible to ascertain if the incidence of NUMTs for the COI barcode region mirrored that for other segments of the mitogenome (e.g. [28, 29]). We first used ‘ncbi-acc-download’ (https://github.com/kblin/ncbi-acc-download) to obtain mitogenomes from the NCBI Organelle Genome Resources (https://www.ncbi.nlm.nih.gov/genome/organelle/. On 12/1/21, this repository included mitogenomes for 2,897 insect species. Of these, 391 overlapped with our 1,446 species while mitogenomes for another 13 species were archived with their nuclear genome assembly. Among these 404 NCBI-sourced mitogenomes, 219 were annotated, while 185 were not. As a final step, because genome assemblies can possess ‘overlooked’ mitogenomes [30], we screened all nuclear assemblies to identify scaffolds likely to represent unannotated mitogenomes (see S1 File–Identification of new mitogenomes). All mitogenomes lacking an annotation, whether derived from NCBI or from mitogenome mining, were annotated using the MITOS server (http://mitos.bioinf.uni-leipzig.de/index.py; [31]). We then filtered presumptive mitogenomes, retaining only those with all 13 protein-coding genes found in animal mitogenomes [32] and with the standard gene order (see S1 File–Mitogenome filtering and annotation). These filters produced mitogenomes for 440 species (30.4% of the 1,446 total species), of which 332 were from NCBI and 108 were newly recovered from nuclear assemblies.

### Combined COI barcode dataset

The COI barcode dataset needed for NUMT detection was assembled by combining the mitogenome and BOLD datasets as follows. For the 440 species with mitogenomes, we used BEDTools getfasta (v.2.30.0; [33]) to extract the full-length COI sequence and then employed Aliview to isolate the 658 bp barcode region. All 440 mitogenome-derived COI barcodes were then run through the BOLD Identification tool (http://boldsystems.org/index.php/IDS_OpenIdEngine) to verify their derivation from the correct species. This step resulted in the removal of 21 mitogenome-derived barcodes and their source mitogenomes as they were either misidentified or derived from contamination, leaving 419 species. All new mitogenomes (sequence and annotation files) are available on figshare (10.6084/m9.figshare.22939934). Finally, we incorporated COI barcodes for 583 additional species that were represented in the 783 BOLD-derived set but did not overlap with the mitogenome-derived set. Thus, the combined dataset includes COI barcodes for 1,002 of the 1,446 target species (69.3%), including 419 that are mitogenome-derived, and 583 BOLD-derived (see also **S1 Fig**). These sequences are available as a dataset (DS-NUMTINS) on BOLD dx.doi.org/10.5883/DS-NUMTINS.

### NUMT abundance, density, and size distribution

Before analysis, each nuclear genome was filtered to exclude residual mitochondrial DNA sequences. First, we removed all scaffolds with the term ‘mitochondrion’ in their FASTA header. This header is included before each scaffold sequence, and lists a unique identifier for each scaffold, the source organism, and a description of the sequence. The inclusion of ‘mitochondrion’ in the metadata for this subset of scaffolds indicated they were inferred to have a mitogenome origin during assembly. Most of the scaffolds filtered during this stage approximated the size (mean = 11.3 kb) expected for a mitochondrial genome, but some were shorter or longer (range = 0.2–45.5 kb). While short sequences likely represent partial mitogenome scaffolds, the longer sequences could either represent mitogenomes with an expanded intergenic region [34] or NUMTs. As we could not reliably discriminate between these alternatives, we removed the 15 scaffolds with a length exceeding 20 kb. Consequently, our estimates of NUMT abundance should be viewed as conservative. Second, we removed all unannotated scaffolds that we identified as a mitogenome (see S1 File–Identification of new mitogenomes).

We then interrogated the nuclear genomes of the 1,002 species for NUMTs derived from the barcode region using BLASTn searches that employed the COI barcode from each species as the query. BLAST parameters included a maximum expectation value (-evalue = 0.0001) and a percent identity > 60% (-perc_identity 60) to the query. In practice, > 99% of the NUMTs recovered through this approach showed ≥ 65% identity to the query sequence. We only consider BLAST hits ≥ 100 bp in subsequent analyses for two reasons. First, when matches involve sequences < 100 bp, the average BLAST E-value approaches the threshold (10^−6^) considered reliable for DNA-based homology matches [35]. Second, most studies which employ DNA for species identification (e.g. [36–38]) target amplicons ≥ 100 bp so results are unaffected by shorter NUMTs.

We processed the BLASTn results to remove hits with 100% query coverage (± 1 bp) that were also very similar (ID ≥ 99%) to the query COI barcode sequence. We reasoned that such sequences either represented segments of the mitochondrial genome retained in the nuclear data despite our mitigation efforts or recently integrated NUMTs whose presence would have no impact on estimates of species richness because standard protocols employ a > 2% divergence for OTU designation. The remaining hits were presumed to be valid, enabling a conservative count of the COI NUMTs for each species. To investigate the length distribution of NUMTs exceeding the 658 bp COI barcode, we repeated the prior steps using full-length COI sequences (ca. 1,500 bp) as the query, employing records derived from the 419 species with mitogenomes (**S7 Table**).

### NUMT counts for COI versus other regions of the mitogenome

We next determined if the incidence of NUMTs for the COI barcode region was similar to that for other coding regions of the mitogenome as expected if all regions of the mitogenome are equally susceptible to nuclear integration. This analysis employed the fasta_windows_v1.1.sh script (https://github.com/kdillmcfarland/sliding_windows/) to partition each of the 419 mitogenomes into 15–22 non-overlapping fragments matching the COI barcode (i.e., window size = 658 bp; slide size = 658 bp), and including the other 12 protein-coding genes and the two rRNA genes. They were extracted from the full-length mitogenomes using the annotation files and BEDTools getfasta as described for COI above. While the annotation files recovered all 14 genes from most mitogenomes, some *de novo* annotations were incomplete, reducing the apparent length of a few mitogenomes (see S1 File–Mitogenome filtering and annotation). BLAST was used to assess the number of NUMTs derived from each fragment in each species as described for COI barcodes. We then generated a mean NUMT count for the set of fragments from each species to create a mitogenome-wide average and compared it with the NUMT count for the barcode region using a linear model in R v. 4.1.0 [39] and log_2_-transformed the values for both metrics. To confirm that the relationship between these two variables was not impacted by heavy sampling of certain insect genera, the analysis was repeated with a dataset containing one haphazardly selected species per genus.

### Patterns of NUMT variation across insect taxa

We examined the impact of the quality of nuclear genome assemblies (sequence coverage, assembly level) on NUMT counts. A Wilcoxon rank-sum test in R was used to compare NUMT counts from low and high coverage assemblies (see S1 File–Nuclear genome sizes) while the relationship between NUMT counts and contig N50 was evaluated using Spearman’s rank correlation in R. As NUMT counts typically increase with genome size [10], we used Spearman’s coefficient in R to examine the strength of this correlation for the 668 insect species whose high coverage assembly allowed estimation of their genome size.

To visualize variation in NUMT counts among the 668 species and its relationship to genome size, we built separate circular cladograms based on COI barcodes in RAxMLGUI v2.0.7 [40] for the five major orders and the 12 minor orders. We then used the R package “ggtree” [41] to overlay bars showing either NUMT counts or genome size on each cladogram. To test for differences in NUMT counts among orders, we used Kruskal-Wallis rank sum tests in R. Because sample sizes for most orders were low, we restricted this analysis to the five major orders.

### NUMT recognition and impacts on estimates species richness

NUMTs are typically recognized via screens for indels or premature stop codons (IPSCs) [1]. To determine if the NUMTs identified in our analysis were diagnosable, we first searched for indels. Specifically, we screened each NUMT for frameshift indels (i.e., those not in a multiple of three) using a custom R script. We then examined each NUMT for premature stop codon(s) by uploading all NUMTs to BOLD where they were translated and screened for stop codons employed by insect mitogenomes.

To determine the impact of NUMTs on estimates of species richness, we considered five length categories (100–150 bp, 151–300 bp, 301–450 bp, 451–600 bp, 601–658+ bp) recoverable by high-throughput sequencing (HTS) platforms [42–44]. These categories are hereafter designated as C1, C2, C3, C4, and C5. We directed particular attention to the subset of C5 NUMTs (C5*) that spanned the barcode region (651–661 bp), that lacked an IPSC, and that possessed >2% divergence from mtCOI in their source species.

Because NUMTs lacking IPSCs can impact species diagnosis, we compared the proportion of diagnosable NUMTs for each category using a homogeneity chi-square test in R. We also compared the length and nucleotide divergence for diagnosable/non-diagnosable NUMTs of COI using Spearman’s rank sum tests. We employed a 2% sequence divergence threshold to categorize NUMTs lacking IPSCs into either distinct Operational Taxonomic Units (OTUs) that would inflate the species count (NUMTs > 2% divergence) or into haplotypes that would be grouped with their parent species, inflating its intraspecific COI variation (NUMTs < 2% divergence). To ascertain their impact on estimates of species richness, we used the Refined Single Linkage (RESL) algorithm [27] to generate OTU counts for the NUMT array derived from the 668 species for NUMTs with three lengths (150 bp, 300 bp, 658 bp).

### Impact of analytical protocols on NUMT exposure

Any COI NUMT can contain the binding sites for the primers used to recover a segment of the barcode region so long as its length exceeds that of the target amplicon. For example, a 300 bp segment of COI cannot be recovered from C1 and C2 NUMTs, but it might be retrieved from C3–C5 NUMTs with the likelihood of its inclusion being determined by the category’s fractional coverage of the full 658 bp barcode region. Consequently, the NUMT exposure for any category is:

Exposure=meanlengthofcategory/lengthofthebarcoderegion

As a result, the number of C3 NUMTs which will be amplified by a primer set targeting a 300 bp region of COI = # C3 NUMTs multiplied by their exposure (375 bp/658 bp = 0.57). Exposure rises to 0.80 (525/658) for C4, and to 0.96 (625/658) for C5.

### DNA barcodes

Expansion of the DNA barcode reference library requires the assembly of sequence information for the 658 bp 5’ region of COI. Barcode library construction ordinarily targets a single 658 bp amplicon, but DNA extracts with degraded DNA require the examination of two or more amplicons. Work on lightly degraded extracts typically examines two C3 amplicons (307 bp, 407 bp) that jointly span the barcode region [45]. This analytical approach increases the risk of NUMT exposure, and the resultant assembly has three possible compositions (2 mtCOI sequences, 2 NUMTs, mtCOI/NUMT chimera). Studies on heavily degraded DNA extracts, such as those from century-old museum specimens, must usually examine 100–150 bp amplicons [46, 47] so all five categories must be considered with exposure varying 5-fold among the length categories (C1 = 0.19, C2 = 0.34, C3 = 0.57, C4 = 0.80, C5 = 0.96). In this case, total exposure involves summing the values for the five categories.

### Metabarcoding

Metabarcoding studies typically examine freshly collected bulk samples so the resultant DNA extracts contain template that can be amplified for the 658 bp barcode region. However, most studies target a shorter segment (typically < 500 bp; [48–50]) because species recovery is facilitated by high read depth [51,52] and this is achieved more cost-effectively by short-read platforms [42].

### eDNA

After its release into the environment, DNA is rapidly degraded [53, 54]. As a result, most eDNA studies employ primer sets which amplify short (e.g. < 150 bp) templates [55–57] because shorter stretches of DNA persist longer in the environment [58]. Even so, we note that the use of longer fragments in eDNA studies has been increasing (e.g. [59]; reviewed in Cristescu & Hebert [60]).

## Results

### NUMT counts: Impact of sequence coverage and assembly contiguity

BLASTn detected 16,570 (≥ 20 bp) and 9,812 (≥ 100 bp) NUMTs derived from the COI barcode region among the 1,002 species (17 orders, 149 families, 591 genera) with both a genome assembly and DNA barcode sequence. **S1 Table** lists these hits together with their key attributes (sequence, length, similarity to query sequence). Most of these species (987/1,002) had a coverage estimate for their genome assembly. These values varied by six orders of magnitude and were bimodal with the low distribution possessing a mean/median coverage of 1.02x/1.07x while the high distribution had a mean/median of 124.1x/76.0x (**S2 Fig**). As coverage estimates were obtained from NCBI metadata rather than through direct calculation, errors could be present. Given this bimodality in coverage, the break point (5x) between the distributions was used to designate the nuclear assembly for each species as either low coverage (hereafter LC) or high coverage (hereafter HC). The 15 species lacking an estimate were assigned as LC.

**Fig 1:**
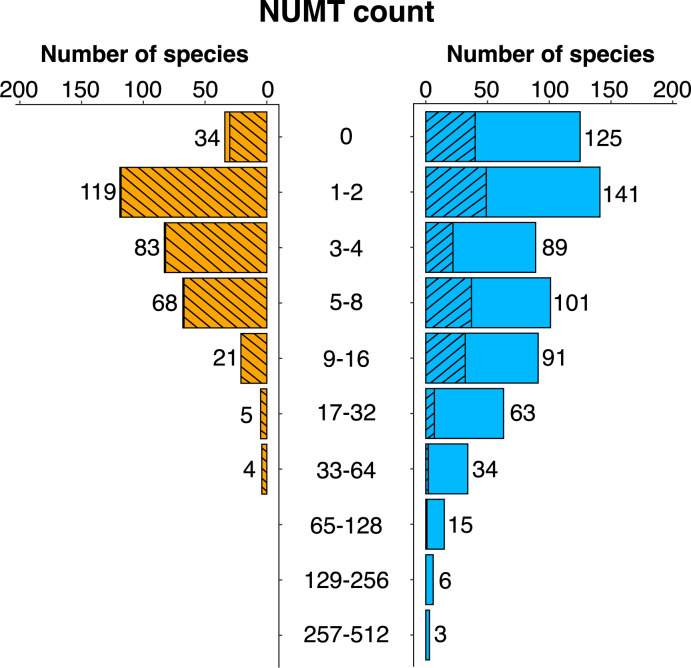
Number of NUMTs (≥ 100 bp) derived from the barcode region of COI for 1,002 insect species. NUMT counts are plotted separately for species with low (334) and high (668) coverage assemblies. Orange = low; blue = high; slashed bars = Lepidoptera. Analysis considered species with both a DNA barcode sequence and a nuclear assembly with a coverage estimate.

The number of COI NUMTs showed marked variation among taxa; 159 of the 1,002 species had none, while the others possessed from 1 to 443 (**Fig 1**). Among the 668 HC species, the number in each log_2_ interval from 0–32 NUMTs per genome showed less than two-fold variation, followed by a halving of the species number with each subsequent doubling in the NUMT count. The 334 LC species showed a similar pattern, but the highest NUMT values were missing, leading to a lower average NUMT count (4.1 versus 12.6) (**Table 1**, **S3 Fig**). However, the HC and LC species groups differ in taxonomic composition: 97.9% of LC species (327/334) were Lepidoptera versus 28.4% in the HC set. The difference in average NUMT count between coverage classes was greatly reduced (LC = 4.1; HC = 5.7) when analysis compared members of this order and became insignificant when analysis only examined the six families in both datasets (Sign test, *P* = 0.22, **S3 Table**). Genome contiguity (contig N50) did not impact counts in the LC (Spearman’s rank correlation: *p* = 0.09, *P* = 0.12, n = 334) or HC (Spearman’s rank correlation: *p* = -0.01, *P* = 0.88, n = 668) assemblies. Because genome size estimates obtained from assembly length were unreliable for LC species (see S1 File–Nuclear genome sizes; **S2 and S4 Figs**), detailed analysis focused on the HC set (**S5 Fig**). In total, they possessed 8,423 NUMTs ≥ 100 bp with counts ranging from 0–443 per species (**Table 1** and **S2 Table**). As 126 of the HC species lacked NUMTs, the others possessed an average of 15.5.

**Table 1:** NUMT attributes for species with high and low coverage nuclear assemblies.

Coverage	n	# NUMTs	Count	Length	Proportion
			Mean/Range	Mean/Range (bp)	with IPSC
High	668	8,423	12.6; 0–443	271 ± 177; 100–754	0.67
Low	334	1,380	4.1; 0–49	254 ± 164; 100–709	0.46

A total of 668 insect species with high coverage nuclear assemblies (≥ 5x) and 334 species with low coverage nuclear assemblies (< 5x) are included. The 15 species lacking an estimate were assigned as LC.

### Lengths and diagnosis of COI NUMTs

**Fig 2:**
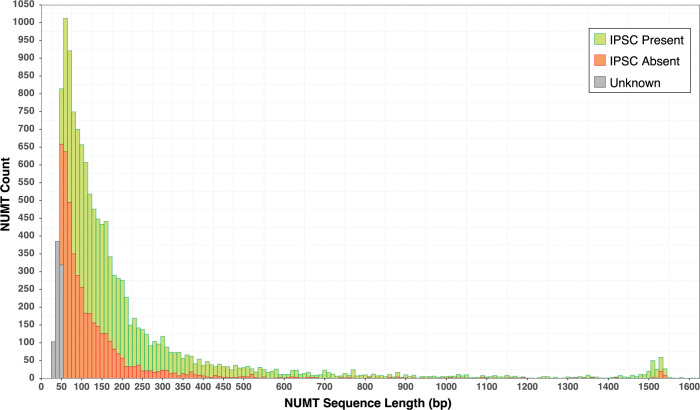
Length distribution of COI NUMTs for 283 insect species as revealed by using a full-length (ca. 1,500 bp) COI query. Lengths only show the region corresponding to COI; the secondary peak circa 1,500 bp reflects NUMTs that extend beyond COI and those with an internal insertion. The proportion of NUMTs with a frameshift indel or premature stop codon (IPSC) is shown for each length category.

When analysis considered COI NUMTs recovered with 658 bp barcode queries, lengths varied from 100–754 bp in the 1,002 species (**Table 1**). Most were short; 30% were < 150 bp, 71% were < 300 bp, and 88% were < 600 bp. NUMTs recovered using a full-length COI query sequence from 283 of the HC species ranged from the low cut-off (100 bp) to circa 1,550 bp, the length of the gene (**Fig 2**). The secondary peak near the upper value was an artifact reflecting the fact that some NUMTs included COI together with upstream and/or downstream gene regions. Ignoring this peak, the length distribution of COI NUMTs closely approximated a Pereto distribution (alpha = 1).

Sequence similarity of the 8,423 NUMTs to their COI barcode query ranged from 64–100% Short NUMTs can be recognized because of their truncation even when they have 100% identity, but full-length NUMTs lacking divergence are undetected as shown by the presence of NUMTs in the lower right but not upper right corner of **S5 Fig**. Two-thirds of these NUMTs possessed IPSCs, but this percentage varied among the five length categories (X^2^ = 190.0; *P* < 10^−5^, df = 4), increasing from 57% in those 100–150 bp to 77% for those 451–600 bp (**Table 2** and **Fig 2**). The percentage of NUMTs > 600 bp with an IPSC declined to 64%, likely reflecting their more recent integration as evidenced by their lower divergence from mtCOI than the other length categories (4.8% versus 10.9%). Considering all NUMT lengths, sequence divergence from the mtCOI query was greater for those with IPSCs (18.6%) than for those without (10.0%) (**S1 Table**). Among the 5,607 NUMTs with an IPSC, 3,571 possessed both diagnostic features; 1,528 only had an indel, and 508 only possessed a stop codon.

**Table 2:** Number of NUMTs with/without IPSCs.

	With IPSC		Without IPSC		
Size Range (bp)	# NUMTs	Mean Divergence (%)	# NUMTs	Mean Divergence (%)	Total
C1(100–150)	1,453	19.1 ± 6.7	1,092	12.01 ± 7.1	2,545
C2(151–300)	2,401	19.6 ± 7.5	978	10.8 ± 7.3	3,379
C3(301–450)	693	19.2 ± 8.1	238	7.9 ± 6.5	931
C4(451–600)	446	18.2 ±9.3	135	7.2 ± 5.9	581
C5(601–661)	614	13.0 ± 8.5	373	4.8 ± 4.1	987
**Total**	**5,607**	**18.6** ± **7.9**	**2,816**	**10.1** ± **7.2**	**8,423**

Five length categories (C1–C5) are considered, including mean sequence divergence between these NUMTs and their mitochondrial COI homologue. Analysis considered the 668 species with a high coverage genome.

### NUMT counts for COI relative to mitogenome-wide counts

**Fig 3:**
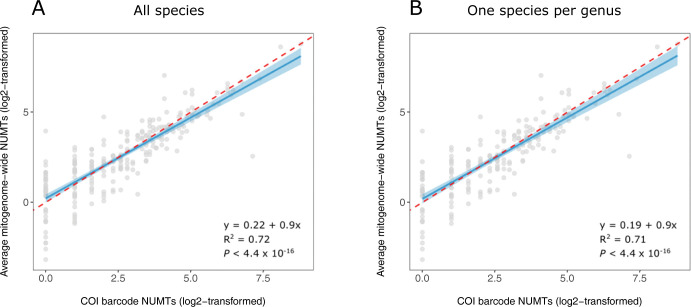
Correlation between NUMT counts for the COI barcode and average mitogenome-wide NUMT counts for 242 species with a mitogenome and ≥ 5x nuclear assembly. 75 species lacking NUMTs were excluded from analysis. (a) 242 species; (b) One species from each of the 191 genera. The red dashed line has an intercept of 0 and a slope of 1.

The NUMT count for the COI barcode region was a strong predictor of the mean count for other mitogenome coding regions (*R*^2^ = 0.72) in the HC species (**Fig 3**). This relationship was unchanged when analysis considered one species per genus (*R*^2^ = 0.71). Moreover, the slope of the regression was close to 1.0 indicating that NUMT counts for COI match those for other coding regions in the mitochondrial genome.

### Variation in genome sizes and COI NUMT counts among insect taxa

Because prior studies have shown that genome size influences NUMT count, all genome size data for insect species were assembled. The resulting compilation included 1,838 species representing 26 of 27 insect orders, and 229 of their 1,000 component families (**S4 Table**). Mean genome size varied 60-fold from 130 Mb in Strepsiptera to 7,737 Mb in Orthoptera (**S5 Table**). While congeneric species had similar genome sizes (**S7 Fig**), those in different families within an order often showed marked divergence. For example, among the nine orders represented by at least five families, the ratio of high/low genome sizes varied 8-fold (22.3–Coleoptera, 2.9–Lepidoptera) (**S6 Table**). Considering all HC taxa, the count of COI NUMTs was positively correlated with genome size (*R*^*2*^ = 34%), and r-squared rose to 56% when counts for the entire mitogenome were considered (**S6 Fig**). Congeneric species showed limited variation in both genome size and NUMT counts (**S7 Fig**). When all NUMTs > 100 bp were considered, there was a 100-fold difference in mean counts among the 17 insect orders (**Table 3**). Among the five major orders, Hemiptera and Hymenoptera had much higher counts (23.0, 17.8 respectively) than Coleoptera, Diptera, and Lepidoptera (9.8, 8.0, 5.3 respectively) (**Table 3**).

**Table 3:** Mean genome size and counts for two lengths of COI NUMTs.

Order	n	Mean Genome Size (Mb)	Mean Count	Mean Count
			≥ 100 bp	≥ 658 bp
Orthoptera	5	2391	140.6	4.4
Phasmatodea	2	2318	138.5	6.0
Blattodea	5	1558	84.0	1.2
Odonata	2	1146	32.5	0.0
Plecoptera	2	371	30.5	0.5
Hemiptera	49	660	23.0	1.0
Ephemeroptera	2	327	1.0	0.5
Siphonaptera	1	776	51.0	0.0
Megaloptera	1	768	28.0	0.0
Hymenoptera	131	330	17.8	0.8
Coleoptera	54	562	9.8	0.7
Diptera	213	284	8.0	0.6
Strepsiptera	1	156	7.0	0.0
Lepidoptera	190	529	5.3	0.3
Trichoptera	7	791	5.0	1.3
Thysanoptera	1	416	1.0	0.0
Neuroptera	2	549	0.0	0.0
**Total**	**668**	**453**	**12.6**	**0.6**

Analysis considers 668 species with genome assemblies ≥ 5x belonging to 17 orders.

**Fig 4:**
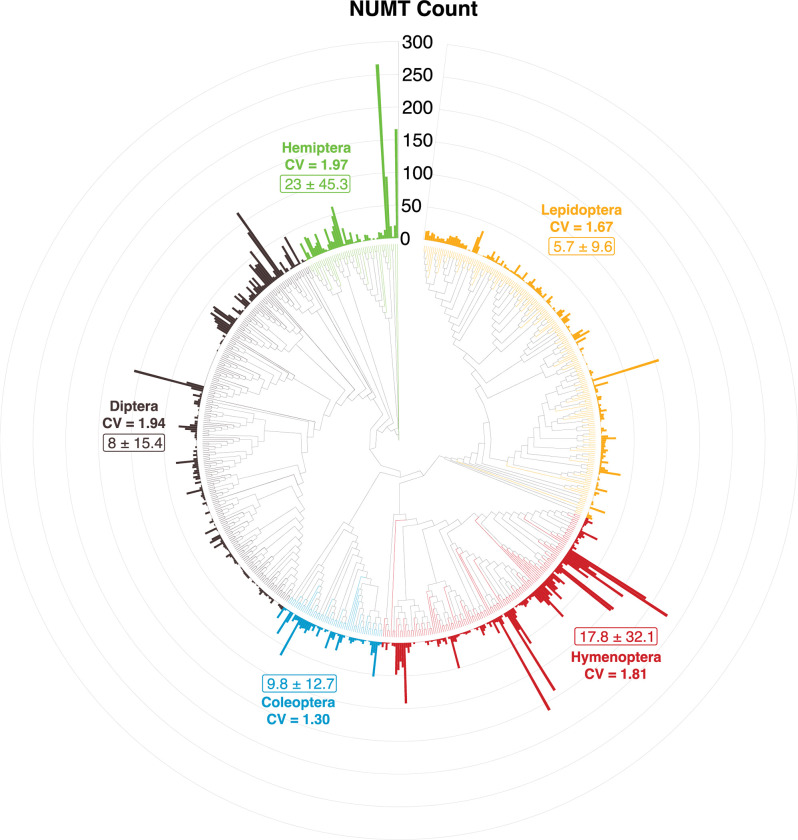
Variation in NUMT count (bar at tip of each node) among the 637 insect species with high coverage genomes from the five major insect orders. This circular cladogram is based on sequence divergence in the 658 bp COI barcode region with ordinal monophyly enforced (RAxML). The node histogram was generated with the R package ‘ggtree’. Rectangles show mean NUMT counts (= /- standard deviation) for each order while CV = coefficient of variation.

**Fig 4** displays the variation in NUMT counts for the 637 species in the five major orders, and an assessment of their intra-ordinal variation. Among them, Hemiptera and Diptera had the most variable NUMT counts (CV = 1.97 and 1.94 respectively), followed by Hymenoptera, Lepidoptera, and Coleoptera. While overall patterns further reveal that allied species generally show congruent NUMT counts, some show marked divergence from closely related taxa. **S8 Fig** displays variation in both genome size and NUMT counts among these taxa while **S9 Fig** provides the same information for the 31 species in the 12 minor orders.

### NUMTs and DNA-based identifications

**Fig 5:**
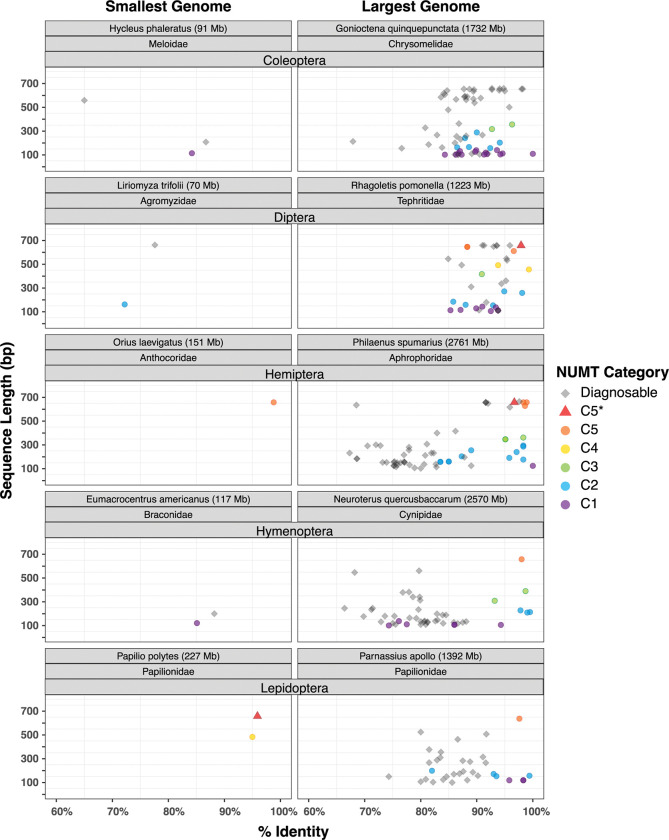
Bivariate plots showing the length and sequence divergence from mitochondrial COI for each NUMT in ten species with the smallest and largest reported genome size for each of the five major insect orders. Gray indicates NUMTs with an IPSC (indel and/or premature stop codon). Other colours indicate five length categories of NUMTs lacking these features.

**Fig 5** displays three key attributes (length, sequence divergence from mtCOI, presence/absence of IPSC) for each NUMT detected in the two species with the greatest genome size difference in the five major insect orders. These paired comparisons show consistently higher NUMT counts in species with large genome sizes. **S10 Fig** expands this representation of counts and attributes to all 668 HC species. Among their 8,423 NUMTs, 5,607 had an IPSC while the other 2,816 did not (**Table 2**). They included all five length categories: 1,092 C1 (100–150 bp), 978 C2 (151–300 bp), 238 C3 (301–450 bp), 135 C4 (451–600 bp), and 373 C5 (600–658+ bp). Most (2,545) of these NUMTs occurred as a single copy in the genome, but others were represented by up to ten copies.

**Fig 6:**
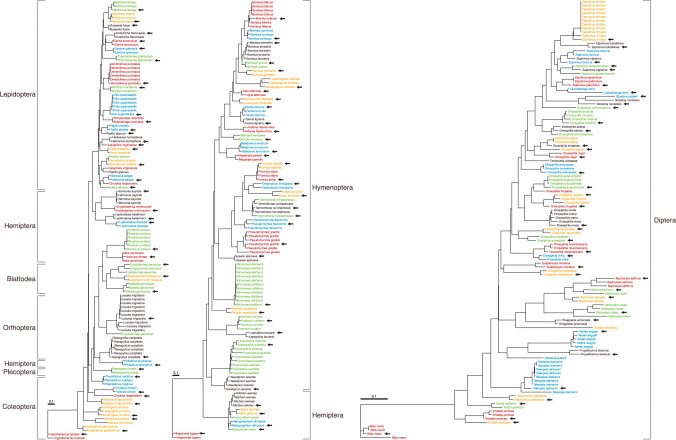
Sequence divergences among 226 C5* NUMTs and the mtCOI from their 113 source species. The maximum likelihood tree was generated using FastTree [61] (doi:10.1371/journal.pone.0009490) using the Jukes-Cantor distance model. Each arrow indicates the mtCOI sequence for a species while colours aid the recognition of conspecific sequences. Based on the 658 bp barcode region, seven of eight orders were recovered as monophyletic (Hemiptera was the exception).

When analysis employs primers for the full barcode region, only C5* NUMTs can inflate the species count. Among the 373 C5 NUMTs, 226 in 113 species were C5*. Most of these species possessed just one or two C5*, but two had ten (**S11 Fig**). In the 69 species with a single C5*, the NUMTs showed a wide range of divergence (2.1–24.2%) from mtCOI and the same pattern extended to species with several C5* (**S12 Fig**). A ML tree indicated that the C5* NUMT(s) in each species typically showed closest affinity to their mtCOI counterpart (**Fig 6**). Species with several C5* often possessed several similar or identical NUMTs dispersed in their genome. For example, all 10 in the hymenopteran *Mimumesa dahlbomi* showed low sequence divergence (0.26%) while 9 of 10 in the dipteran *Zaprionus ornatus* were identical (**Fig 6**). Because of these and similar cases of close sequence similarity, RESL assigned the 226 C5* NUMTs to 139 OTUs, a conversion percentage of 65%. By comparison, RESL assigned the 668 mtCOI sequences from their source species to 632 OTUs, a 95% conversion percentage. If a study recovered all C5* NUMTs, the OTU count for HC species would be inflated by 22% [(139 + 632)/632]. RESL indicated that NUMTs in shorter length categories (150 bp, 300 bp) showed a conversion percentage of 67%, similar to that for C5* (**S13 Fig**).

### Analytical protocols–impact on NUMT exposure

NUMT exposure varies fivefold among the three analytical protocols employed to recover a full-length barcode sequence (**Table 4**). Studies targeting a single 658 bp amplicon could encounter up to 226 C5* amplicons, 34% of the species count. By comparison, studies targeting two amplicons (307/407 bp) could recover 578 NUMTs (87% of species count), while the analysis of 100–150 bp amplicons could encounter 1,118 NUMTs (167% of species count). Because a third of the NUMTs in each length category have identical or similar sequences, the count of distinct OTUs would show less inflation: 22%, 58%, and 111% respectively. As metabarcoding and eDNA studies typically examine just a single amplicon, this reduces NUMT exposure in comparison to the multi-amplicon approaches required to recover full-length barcodes from degraded DNA. However, because they typically target short amplicons, both approaches, particularly eDNA, are exposed to the inflation of the OTU counts caused by NUMTs.

**Table 4:** Impact of analytical protocol on exposure to non-diagnosable NUMTs (i.e., those without an IPSC) for the 668 HC species.

*Protocol*	# Amplicons	Length (bp)	# NUMT x Exposure	Total/Species
*Barcode Library*	1	651–661	C5* = 226 x 1	226/668 = 0.34
			**TOTAL = 226**	
*Barcode Library*	2	307, 407	C3 = 238 x 0.57 +	578/668 x 2 = 1.74
			C4 = 135 x 0.80 +	
			C5 = 373 x 0.96	
			**TOTAL = 578**	
*Barcode Library*	5	100–150	C1 = 1092 x 0.19	1118/668 x 5 = 8.35
			C2 = 978 x 0.34	
			C3 = 238 x 0.57	
			C4 = 135 x 0.80	
			C5 = 373 x 0.96	
			**TOTAL = 1118**	
*eDNA*	1	100–150^a^	C1 = 1092 x 0.19	1118/668 = 1.67
			C2 = 978 x 0.34	
			C3 = 238 x 0.57	
			C4 = 135 x 0.80	
			C5 = 373 x 0.96	
			**TOTAL = 1118**	
*Metabarcoding*	1	300–450	C3 = 238 x 0.57 +	0.87
			C4 = 135 x 0.80 +	
			C5 = 373 x 0.91	
			**TOTAL = 578**	

For NUMT # see Table 2. Total/species = # of amplicons x NUMT exposure/668. See Materials section for explanation of the exposure value.

^a^ fragments analysed in eDNA studies can also be longer (e.g. 313 bp when using the Leray primer set [62]), in which case the impact of NUMTs will be similar to estimates provided for the metabarcoding protocol.

## Discussion

The presence of NUMTs in insect genomes has been known for 40 years [63], but details on their abundance and attributes have only slowly gained clarity. Early studies revealed that NUMTs range widely in size [4], that NUMT counts vary among taxa [8], and that sequence change slows after nuclear integration [1, 64]. Because of the latter property, NUMTs can illuminate deep time events [65, 66]. However, they can also obscure the present, especially for approaches that employ mitochondrial gene regions as a basis for specimen identification and species discovery [67, 68]. This complexity arises because DNA-based biodiversity assessments employ primers that amplify the target region in diverse taxa so they also amplify NUMTs within their nuclear genomes.

Although past work has revealed NUMTs in many insect lineages [8, 12, 19, 29, 69–71], no prior study has systematically characterized their abundance and attributes. In addressing this gap, the present study confronted some limitations. Coverage was too low for a third of the nuclear assemblies to allow the estimation of genome size. Among those with high coverage, 20% lacked a mitogenome or corresponding COI sequence although they undoubtedly resided in the sequence data employed to generate the nuclear assembly [30]. This conclusion was reinforced by the fact that the present study recovered 108 new mitogenomes by simply inspecting the sequences used for assembly of the nuclear genome. As genome sequencing programs expand, the joint assembly of mitochondrial and nuclear genomes should be expected.

### Variation in NUMT counts

Despite data constraints, this study has provided a good overview of NUMT counts and distribution across the class Insecta. COI NUMTs were detected in 16 of the 17 orders examined, and the exception (Neuroptera) was only represented by two species. Among the 668 HC species, 126 lacked COI NUMTs (≥ 100 bp), but the others averaged 15.6 with counts ranging from 1 to 443. Some previous studies (e.g. [28, 29]) suggested that the COI barcode region might be incorporated more frequently into the nuclear genome than other mitochondrial segments, but our mitogenome-wide scan does not support this hypothesis. Specifically, we found that the count of NUMTs from the COI barcode region is a strong predictor of mitogenome-wide NUMT counts, with a regression slope close to 1 (**Fig 3**). In accord with expectations [10], NUMT counts were positively correlated with genome size, and *R*^*2*^ rose from 34% to 56% when analysis extended from the COI barcode to the entire mitogenome. As the barcode region represents just 4% of the mitochondrial genome, this increase was expected, but it does mean that the prediction of COI NUMT counts from genome size will be imprecise. However, given this correlation and the mean count of 13 NUMTs for the barcode region, insect genomes likely possess an average of about 325 NUMTs.

Although larger sample sizes are required to tighten confidence estimates, our analysis revealed a 100-fold difference in mean COI NUMT counts among the 17 insect orders with genome data. This variation largely reflected the interplay between a deterministic factor, genome size, and a stochastic process, the inclusion of COI versus another mitochondrial region in the NUMT array for a species. Because of their generally small genome sizes, NUMT counts were usually low in the four orders that comprise > 90% of all insect species–Coleoptera, Diptera, Hymenoptera, and Lepidoptera [72]. As Orthoptera has, by far, the largest mean genome size of the 27 orders, it is no surprise that the first insect NUMT was discovered in it [63], and that many subsequent studies highlighting the complexities introduced by NUMTs have focused on orthopterans [17, 28, 69, 73, 74]. While the present results confirm that COI NUMTs occur in most insect genomes, they do indicate that they are much less common in the most species-rich orders.

The current results provide a first sense of taxa within each major order with elevated NUMT counts, but more data are needed to allow a lineage-by-lineage threat assessment. For example, the species of Coleoptera examined in this study displayed little variation in NUMT counts, but the family with the largest genome size (e.g. Phengodidae) was not represented. Similarly, the low NUMT count for Lepidoptera reflected results from just a third of its families. Among 37 families of Hymenoptera, the Cynipidae possessed much larger genome sizes than the others, and its members showed elevated NUMT counts. However, some species in other families (e.g. Apidae, Formicidae) had high NUMT counts despite a small genome size, indicating that risk assessments will require consideration of other factors. Importantly, representatives of the most species-rich families of insects (e.g. Braconidae, Cecidomyiidae, Chironomidae, Ichneumonidae, Phoridae) all had low NUMT counts.

### NUMT attributes and recognition

Aside from documenting their prevalence and distribution, this study has clarified two attributes that determine the influence of NUMTs on measures of species diversity–their lengths and the fraction with an IPSC. Nearly 50% of the COI NUMTs detected in this study were too short (< 100 bp) to impact most biodiversity assessments, but species did possess an average of 12.6 NUMTs above this threshold. With a mean length of 272 bp, just 5% spanned the 658 bp barcode region and two thirds of them had an IPSC. Although 83% of the 668 species lacked a NUMT that could inflate the species count when analysis targeted an amplicon spanning the barcode region, the other 113 species possessed one or more NUMTs with the collective potential to raise perceived species richness by 22%. Our results further suggest that analyses examining other protein-coding segments of the mitogenome would generate similar inflation while studies examining 12S/16S rDNA will increase exposure 3-fold because an IPSC filter cannot be applied.

### Impact of NUMTs on DNA-based identification workflows

The much higher copy number of mtCOI should reduce exposure to NUMTs. On average, diploid insect nuclear genomes are about 60,000x larger than their mitochondrial counterparts (1,000 Mb versus 16 Kb). In extracts prepared from whole insects, mtDNA typically comprises less than 0.5% of the total DNA [75]. Presuming two copies of each NUMT per nuclear genome, mitochondrial gene regions will enter PCR with a 150x higher count (60,000 x .005/2). While this difference favours their recovery, variation in amplification can erase it (e.g., a NUMT with a 20% higher PCR efficiency will dominate the final amplicon pool after 35 cycles). The risk of recovering a mix of mtCOI and its NUMT amplicons will extend to every species whose nuclear genome includes binding sites for the primers employed. As a consequence, NUMTs pose risks to all workflows underpinning DNA-based biodiversity assessments–from construction of the DNA barcode reference library to its use for inferring the species composition of environmental samples whether by metabarcoding or eDNA. If all NUMTs were recovered, OTU counts would be inflated by 22% if analysis targeted 658 bp amplicons, by 58% at 300 bp, and by 111% at 150 bp. These inflation factors presume our analysis recovered all NUMTs in the 1,002 species, but this is not the case as polymorphic NUMTs can only be detected when multiple genomes of each species are available for analysis [5, 76]. Finally, we note that our assessment of NUMT impacts considered only OTU counts (i.e., richness). Measures of diversity that take into account both richness and evenness (e.g. Simpson’s index and Shannon’s index [77]) could alleviate some of these impacts, depending on the relative frequency of NUMT-rich taxa in the biological communities under investigation.

## Conclusions

This study indicates that the interpretational complexities introduced by NUMTs for studies of insect biodiversity vary with taxonomy, analytical protocol, and target gene region. It reveals that most species (83%) lack NUMTs long enough to complicate barcode recovery when analysis targets the full 658 bp barcode region. However, the remaining species present an exposure risk because they possessed enough C5* NUMTs to increase the perceived species count by 22% if all were recovered. Because shorter amplicons are more abundant, their analysis has the potential to further inflate the species count. Finally, the gene region targeted for analysis can impact NUMT exposure. Ribosomal genes (12S, 16S) triple exposure because their NUMTs cannot be filtered via IPSC scans.

The present study only considered insects, but a similar analysis on marine invertebrates generated congruent results [78]. The complexities introduced by NUMTs can be further managed by improving informatics platforms and by modifying existing molecular protocols. Improvements to informatics platforms can include filtering based on open reading frame length and using nucleotide profiles built via hidden Markov model analyses of large COI barcodes datasets [79]. As well, they can include filtering based on IPSCs, combined with alignments to known COI barcode datasets [68]. As an additional informatics mitigation strategy, BOLD could include a repository for all C5* NUMTs. Based on this study, their inclusion would only increase the overall sequence count by about 30%. Such a resource would then be used, via a database match step, to exclude NUMTs from new sequence data. As well, curated databases of COI barcodes (e.g. [80]) can help identify records that derive from mtCOI in a manner similar to that described in Andujar et al. [68]. Mitigation strategies involving molecular protocols should maximize the length of DNA segments used in metabarcoding and eDNA workflows. Continued improvements in third-generation sequencers will likely facilitate these efforts, and the transition to longer amplicon lengths (e.g., [81]). Lastly, molecular protocols such as long-range PCR and reverse-transcription (RT)-PCR can help discriminate NUMTs from their mtCOI counterparts. RT-PCR in particular is a notable mitigation strategy, as it exploits the fact that NUMT sequences are not transcribed [78, 82].

While this study has clarified the threats posed by NUMTs, empirical work is needed to understand their actual recovery and the factors that influence it. For example, does the higher initial template concentration of mtCOI diminish the risk of NUMT recovery? Is the ratio of NUMT/mtCOI reads stable for a particular species or, if not, what explains the variation? These investigations have begun (per obs.), but they must be expanded to both understand and mitigate the impacts of NUMTs on biodiversity assessments.

## Supporting information

S1 Fig Overview of the data analysis pipeline. Colour-coding is used to differentiate between the assembly and filtering of datasets, as well as analyses focusing on COI-derived NUMTs or mitogenome-wide NUMTs. Smaller boxes with gray shading are used to list the main computational methods employed for each task.
(PDF)

S2 Fig Relationship between sequence coverage (log-transformed) and assembly length of the nuclear genome (log-transformed). Pearson’s correlation coefficient was obtained separately for low (< 5x) and high (≥ 5x) coverage assemblies. *P* values are corrected for multiple comparisons using the Bonferroni method. Grey circles show 15 assemblies that were included among insect genomes on NCBI although they were bacterial (see S1 File–Nuclear genome sizes). The histogram shows the log-transformed distribution of sequence coverage. The dotted line indicates 5x coverage.
(PDF)

S3 Fig Test of the statistical significance of the number of NUMTs (≥ 100 bp) derived from the barcode region of COI for 334 species with low genome coverage and 668 species with high genome coverage. Wilcoxon rank-sum test; *P* = 1.2 x 10^−4^.
(PDF)

S4 Fig Relationship between flow cytometry estimates of genome size (log-transformed) and nuclear genome assembly length for 148 insect species with both metrics. Only species with high coverage assemblies (≥5x) were included. The inset shows the same analysis performed for a reduced dataset of one species per genus (*N* = 74 species). Pearson’s correlation coefficient was calculated for both analyses with *P* values corrected for multiple comparisons using the Bonferroni method.
(PDF)

S5 Fig Plot of the 8,423 COI NUMTs (≥ 100 bp) identified in high coverage nuclear genomes from 668 insect species. The length of each NUMT is shown as well as its sequence divergence from mitochondrial COI. Values > 658 bp arise through insertions while those < 658 bp reflect deletions or the original incorporation of a truncated fragment. Yellow = NUMT with a frameshift indel and/or a stop codon. Red = NUMT lacking these features.
(PDF)

S6 Fig Correlation between log assembly length and log2 count of COI NUMTs (≥ 100 bp) for species with ≥ 5x coverage. Above: NUMT counts for COI barcode region (n = 668). Below: Mean NUMT counts for 658 bp segments of entire mitogenome (n = 391).
(PDF)

S7 Fig Correlation in log_2_ NUMT (≥ 100 bp) counts and log_2_ genome size for 72 pairs of congeneric species. (PDF)

S8 Fig Circular cladograms based on sequence divergence in the 658 bp COI barcode region for 637 insect species in the five major insect orders with high coverage nuclear genomes. Bars at the tip of each node indicate NUMT count or genome size.
(PDF)

S9 Fig Circular cladograms based on sequence divergence in the 658 bp COI barcode region for 31 insect species in 12 minor insect orders with high coverage nuclear genomes. Bars at the tip of each node indicate NUMT count or genome size.
(PDF)

S10 Fig Bivariate plots showing variation in length and sequence divergence from mtCOI for each NUMT in the 668 species with high coverage assemblies organized by order and family. Gray indicates NUMTs with IPSCs (indels or premature stop codons) that enable their recognition and exclusion. Other colours show the varied length categories of NUMTs lacking IPSCs.
(PDF)

S11 Fig Genome size and the number of C5* COI NUMTs in the 668 species with a high coverage genome. (PDF)

S12 Fig Plot of C5* COI NUMTs (i.e. no IPSC, > 2% divergence from mitochondrial homologue, length = 651–661 bp) in 113 species. All C5* NUMTs (solid circles) from a species are connected via dotted lines to a point representing the mean divergence (open circles) for that species (this connection is absent in species with only one C5* NUMT). The other 555 HC species lacked C5* NUMTs. The other orders include Orthoptera (lanes 5 & 9), Phasmatodea (lane 8), Blattodea (lane 1 = 7% divergence, lane 2 & 3), and Plecoptera (lane 1 = 2% divergence).
(PDF)

S13 Fig The relationship between the number of Operational Taxonomic Units for two length categories of NUMTs (150 bp, 300 bp). Data was obtained using a sliding window analysis across the 658 bp barcode region using RESL [27].
(PDF)

S1 Table 16,584 BLASTn hits for the 1,002 insect species considered in this study. The hit sequence, hit sequence length, and percent identity to the query sequence are shown, along with taxonomic information on each species, as well as details on genome assemblies. Hit sequence lengths and contig N50 scores are in bp; genome assemblies are in Mb; genome size estimates based on flow cytometry measurements (FCM) are in bp. Each assembly is assigned to a coverage category–low (< 5x) or high (≥ 5x).
(XLSX)

S2 Table NUMT summary for each of the 668 insect species with a high coverage nuclear assembly. Count, mean sequence length (bp), and sequence similarity to its mitochondrial COI counterpart are shown. The NUMT array is provided for each species as well as separately for NUMTs with/without IPSCs. Taxonomic assignments, nuclear genome assembly size (Mb), and contig N50 values (bp) are included for each species.
(XLSX)

S3 Table Number of species in each of 28 families of Lepidoptera represented in the high (190 species) or low (327) coverage datasets. (DOCX)

S4 Table Genome size estimates for 1,843 insect species. Representatives from 229 families are included, compiled from two sources: 889 species with ≥ 5x coverage from NCBI and 954 species from cytometry [83]. Cytometry estimates were only retained for species lacking a genome assembly.
(XLSX)

S5 Table Mean genome sizes (Mb) for 26 insect orders and the distribution of mean genome sizes for their 128 component families with genome size data. ^ Order employing direct development, * order with incomplete metamorphosis. Those lacking a symbol develop via complete metamorphosis. n = number of families examined in each order.
(DOCX)

S6 Table Ratio of largest/smallest mean genome size for families in nine insect orders. Analysis only considered orders represented by five or more families. * represented by a single species.
(DOCX)

S7 Table List of the 419 insect species with a mitogenome employed in this study and its source. (DOCX)

S1 File Supplementary materials. (DOCX)
