# Supplementary figures and images for: Interrogating 1000 insect genomes for NUMTs: A risk assessment for estimates of species richness
Source: PLoS One. 2023 Jun 8;18(6):e0286620. doi: 10.1371/journal.pone.0286620 (PMC10249859; doi:10.1371/journal.pone.0286620)

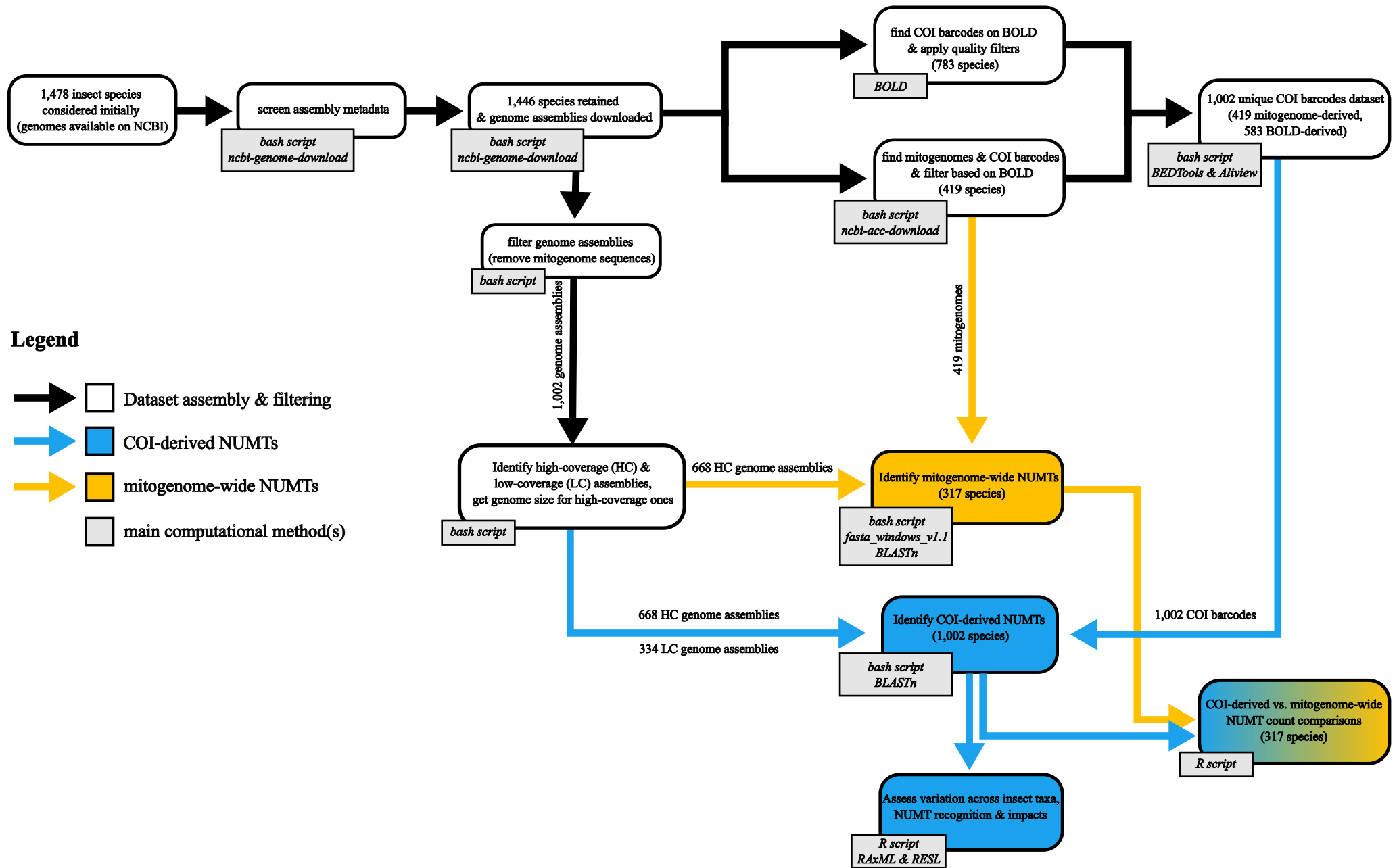

Supplement: S1 Fig — Colour-coding is used to differentiate between the assembly and filtering of datasets, as well as analyses focusing on COI-derived NUMTs or mitogenome-wide NUMTs. Smaller boxes with gray shading are used to list the main computational methods employed for each task. (PDF) [file pone.0286620.s001.pdf]

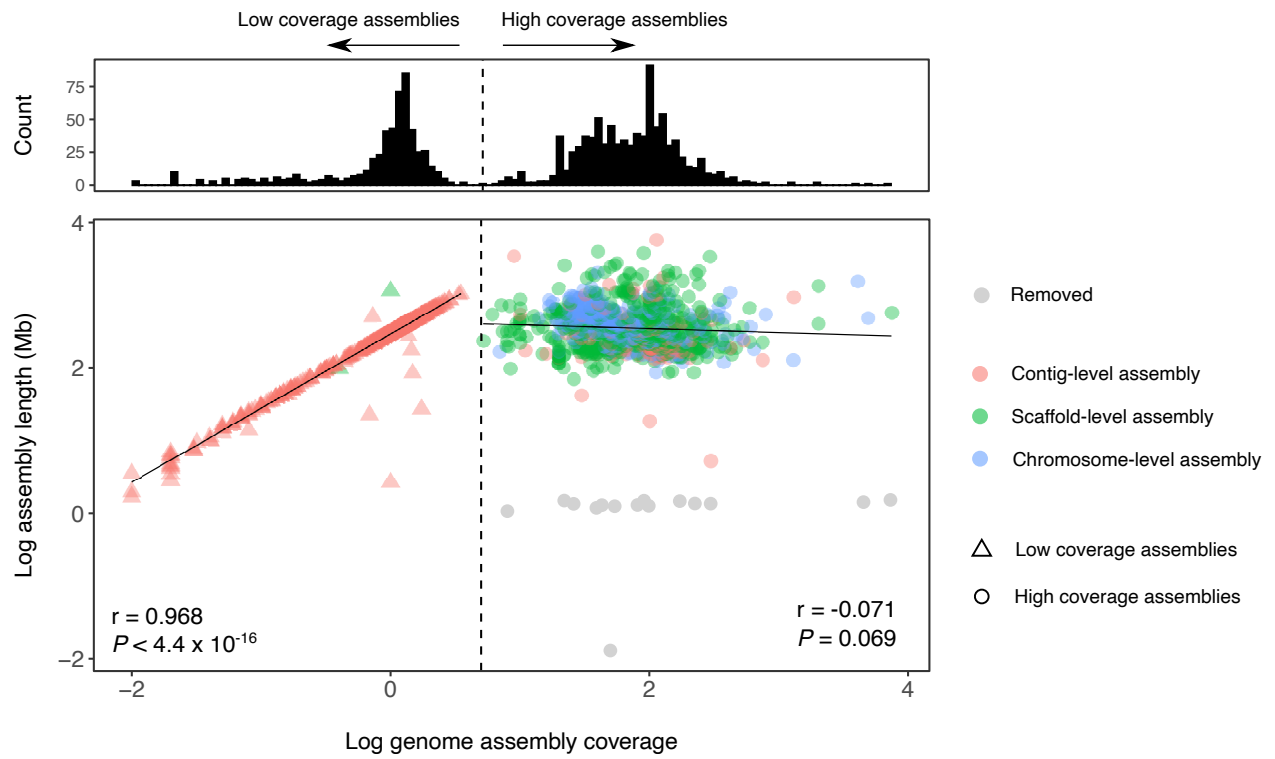

Supplement: S2 Fig — Pearson’s correlation coefficient was obtained separately for low (< 5x) and high (≥ 5x) coverage assemblies. P values are corrected for multiple comparisons using the Bonferroni method. Grey circles show 15 assemblies that were included among insect genomes on NCBI although they were bacterial (see S1 File–Nuclear genome sizes). The histogram shows the log-transformed distribution of sequence coverage. The dotted line indicates 5x coverage. (PDF) [file pone.0286620.s002.pdf]

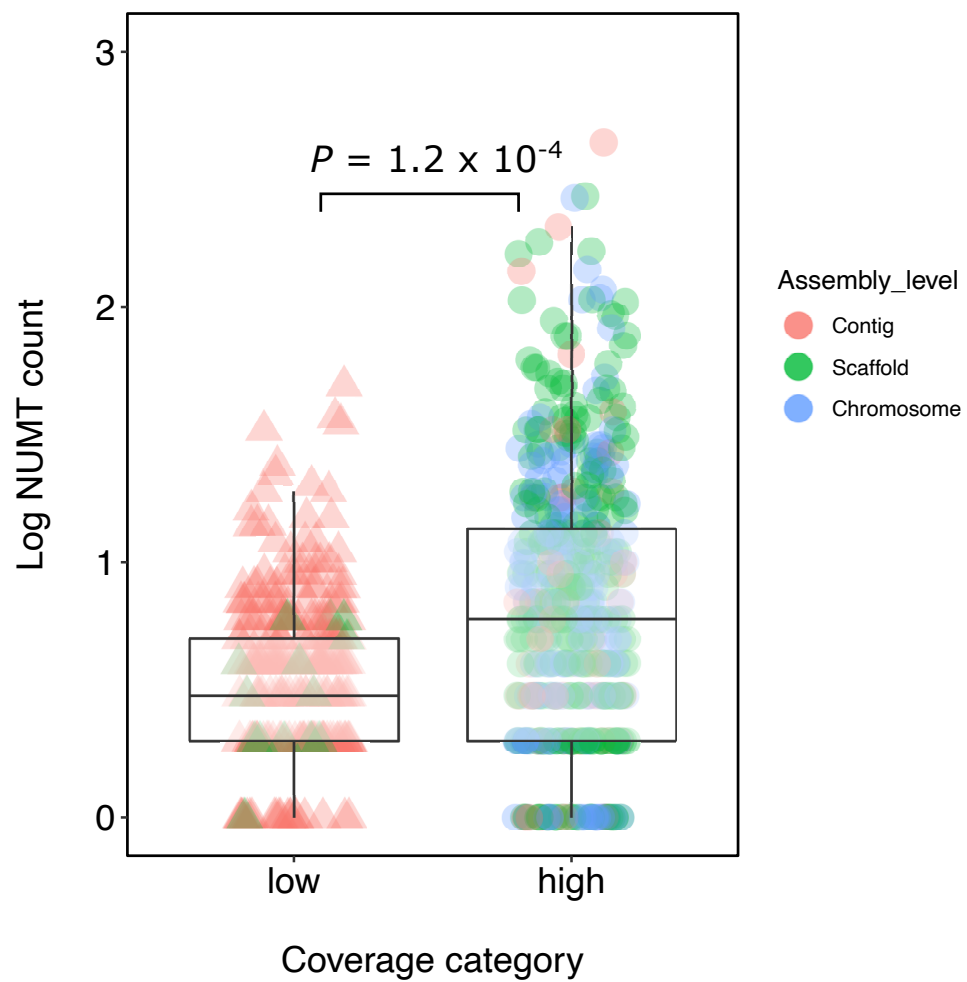

Supplement: S3 Fig — Wilcoxon rank-sum test; P = 1.2 x 10^−4^. (PDF) [file pone.0286620.s003.pdf]

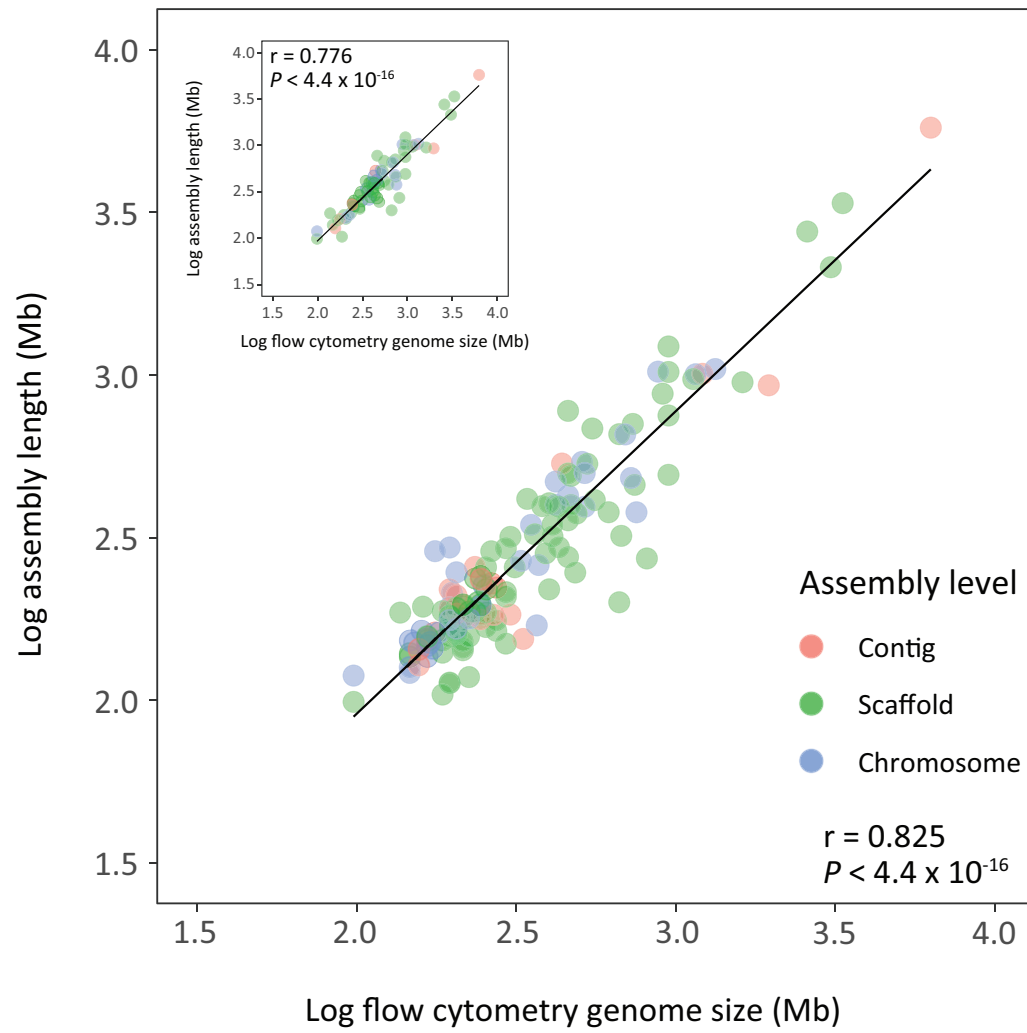

Supplement: S4 Fig — Only species with high coverage assemblies (≥5x) were included. The inset shows the same analysis performed for a reduced dataset of one species per genus (N = 74 species). Pearson’s correlation coefficient was calculated for both analyses with P values corrected for multiple comparisons using the Bonferroni method. (PDF) [file pone.0286620.s004.pdf]

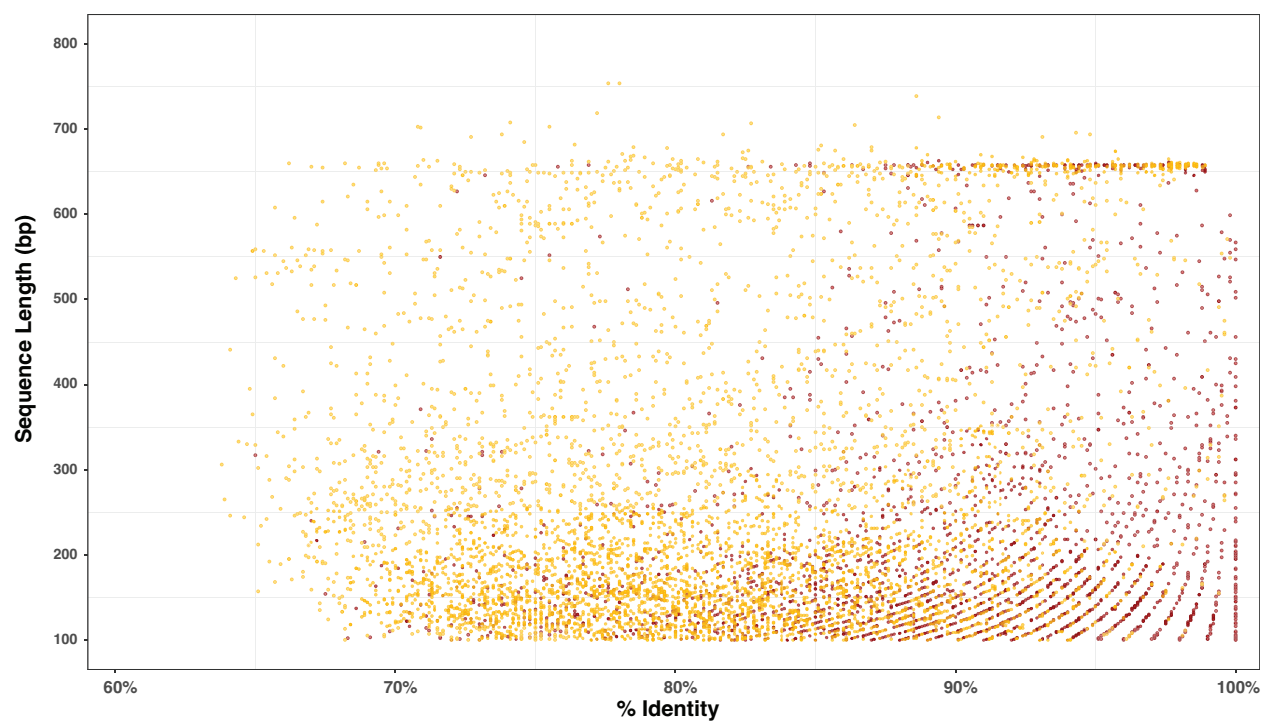

Supplement: S5 Fig — The length of each NUMT is shown as well as its sequence divergence from mitochondrial COI. Values > 658 bp arise through insertions while those < 658 bp reflect deletions or the original incorporation of a truncated fragment. Yellow = NUMT with a frameshift indel and/or a stop codon. Red = NUMT lacking these features. (PDF) [file pone.0286620.s005.pdf]

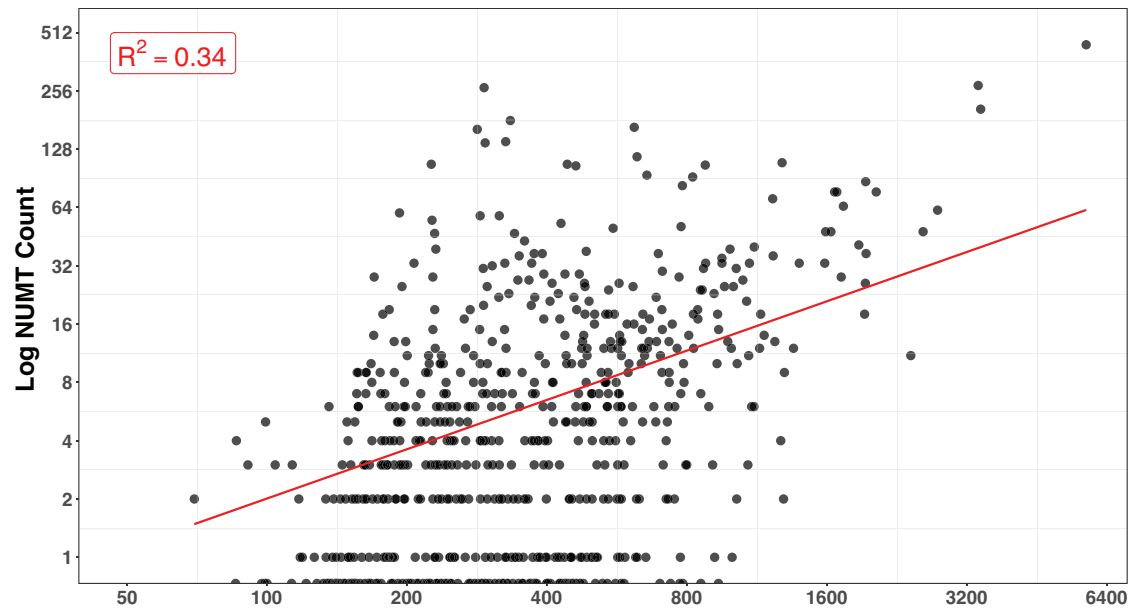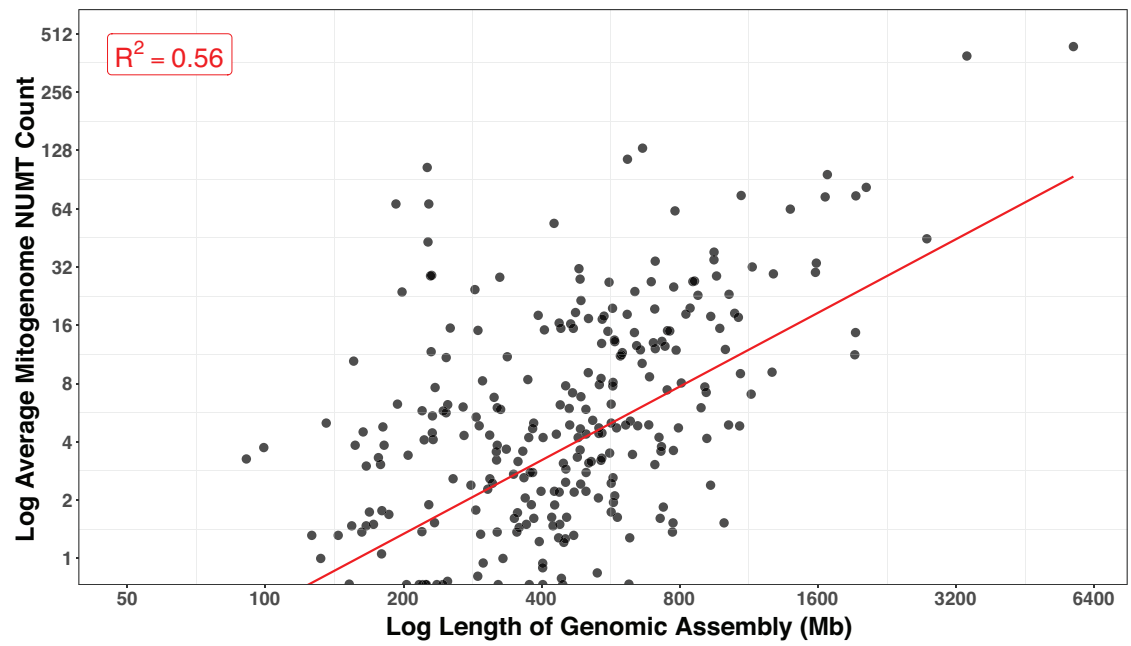

Supplement: S6 Fig — Above: NUMT counts for COI barcode region (n = 668). Below: Mean NUMT counts for 658 bp segments of entire mitogenome (n = 391). (PDF) [file pone.0286620.s006.pdf]

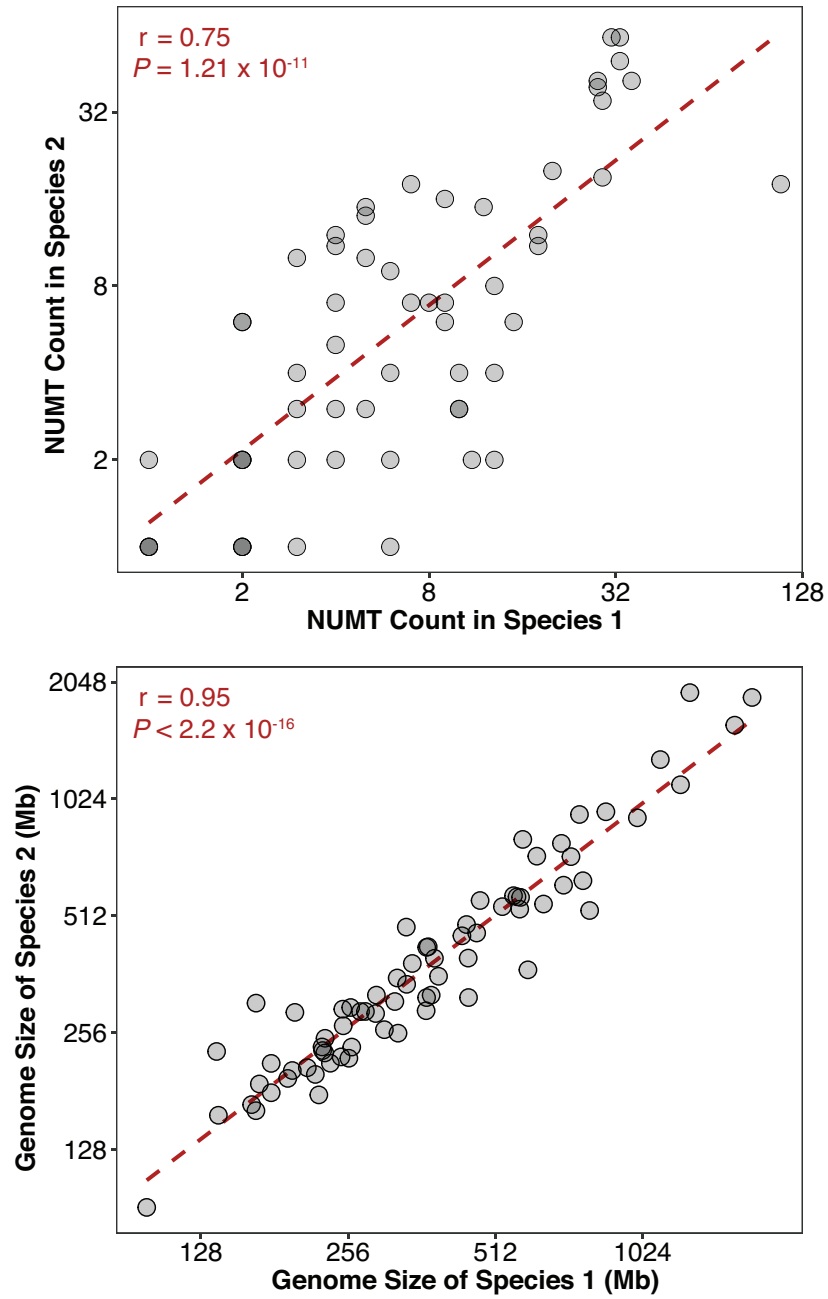

Supplement: S7 Fig — (PDF) [file pone.0286620.s007.pdf]

**NUMT Count**

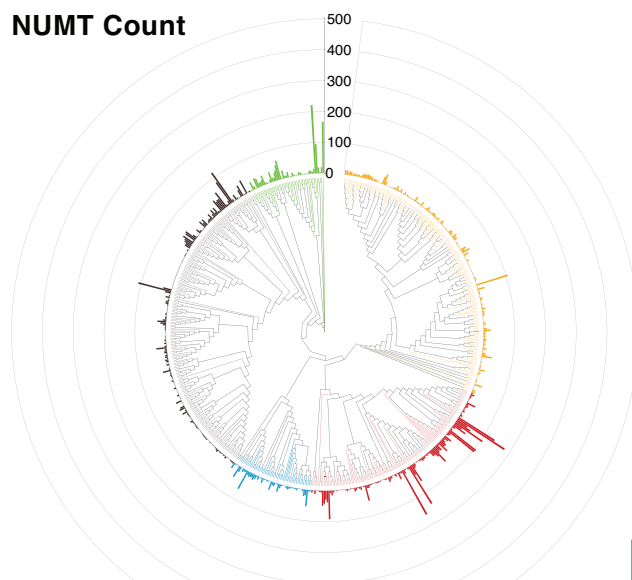

**Genome Size (Mb)**

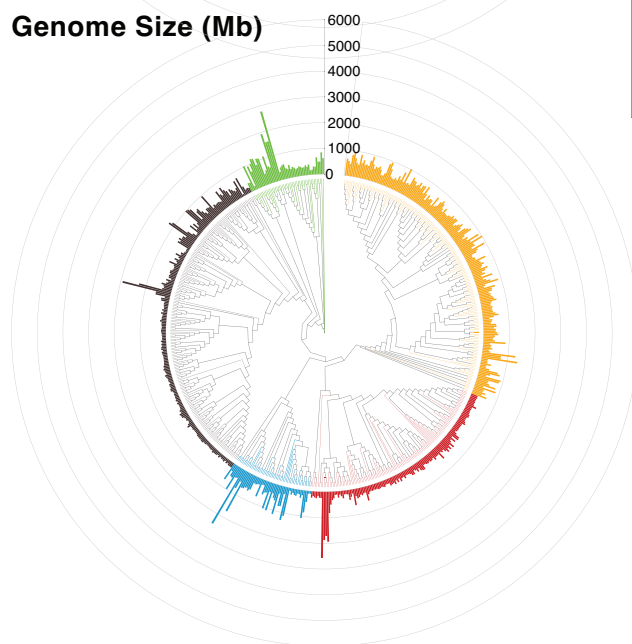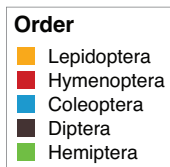

Supplement: S8 Fig — Bars at the tip of each node indicate NUMT count or genome size. (PDF) [file pone.0286620.s008.pdf]

**NUMT Count**

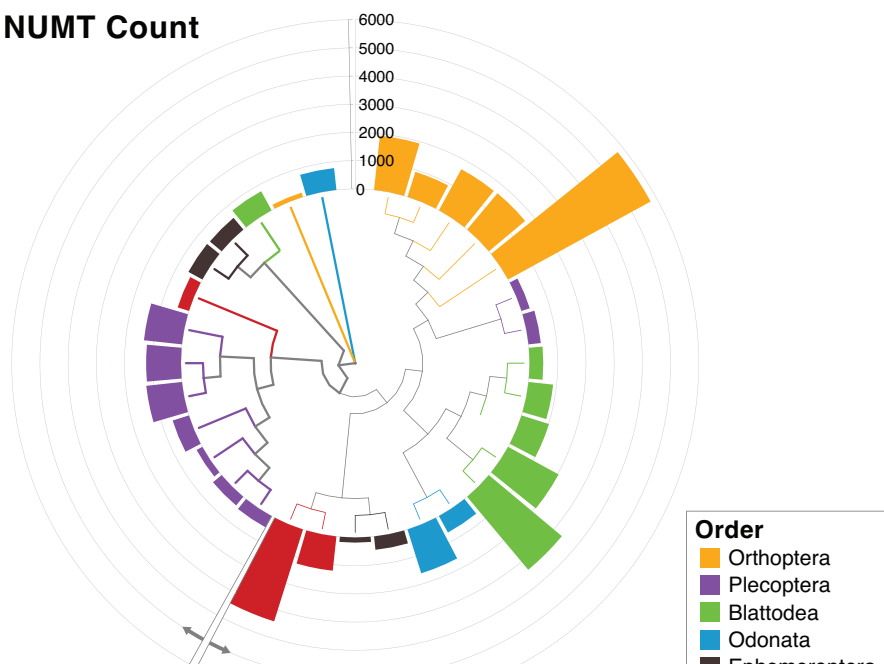

**Genome Size (Mb)**

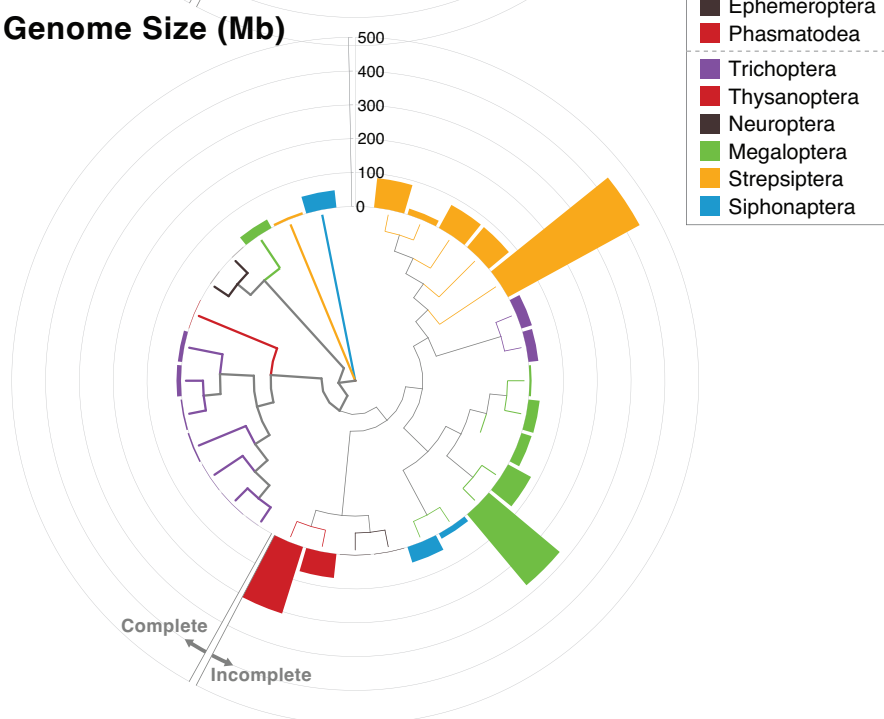

Supplement: S9 Fig — Bars at the tip of each node indicate NUMT count or genome size. (PDF) [file pone.0286620.s009.pdf]

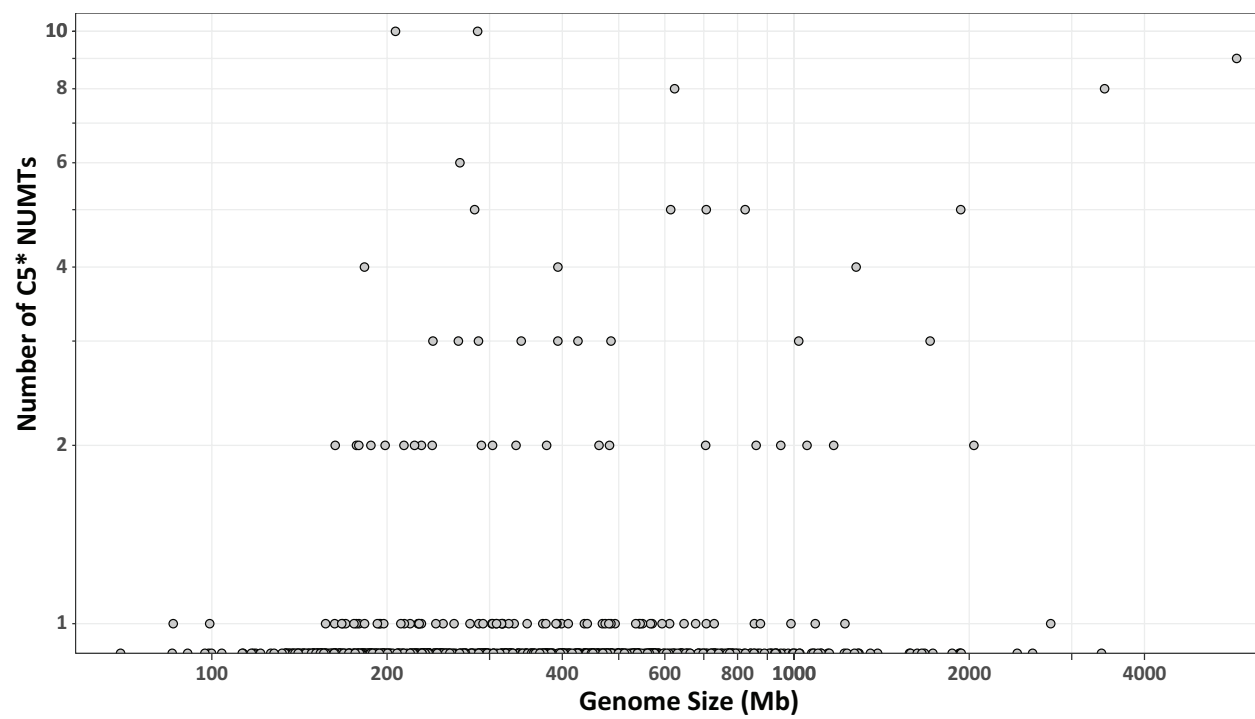

Supplement: S11 Fig — (PDF) [file pone.0286620.s011.pdf]

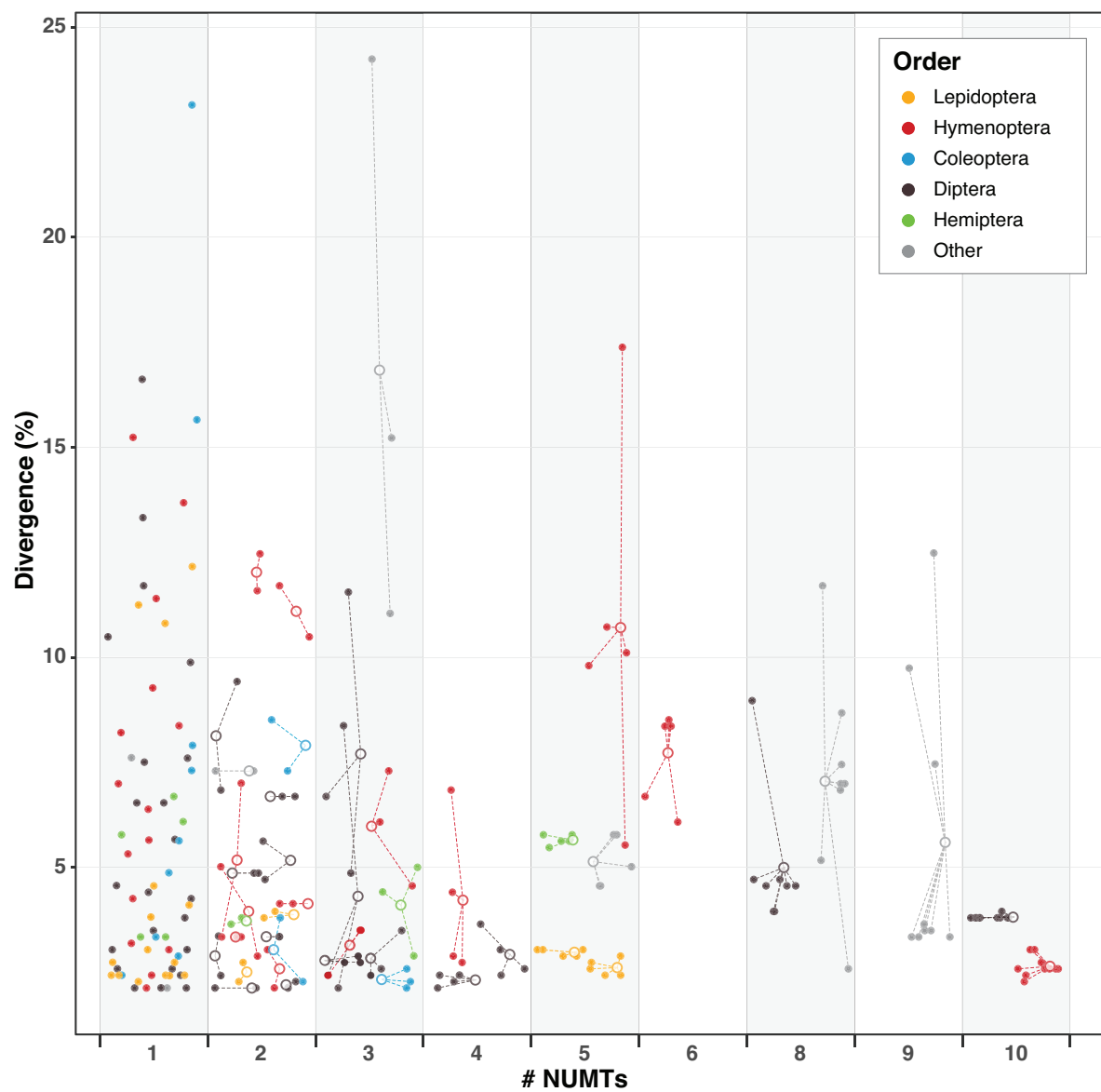

Supplement: S12 Fig — All C5* NUMTs (solid circles) from a species are connected via dotted lines to a point representing the mean divergence (open circles) for that species (this connection is absent in species with only one C5* NUMT). The other 555 HC species lacked C5* NUMTs. The other orders include Orthoptera (lanes 5 & 9), Phasmatodea (lane 8), Blattodea (lane 1 = 7% divergence, lane 2 & 3), and Plecoptera (lane 1 = 2% divergence). (PDF) [file pone.0286620.s012.pdf]

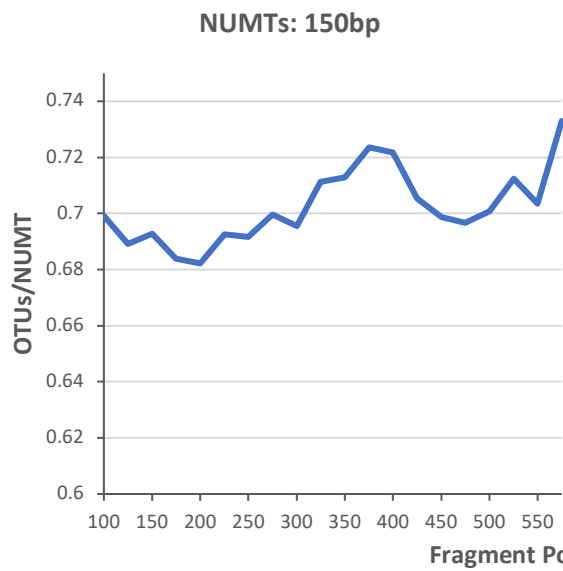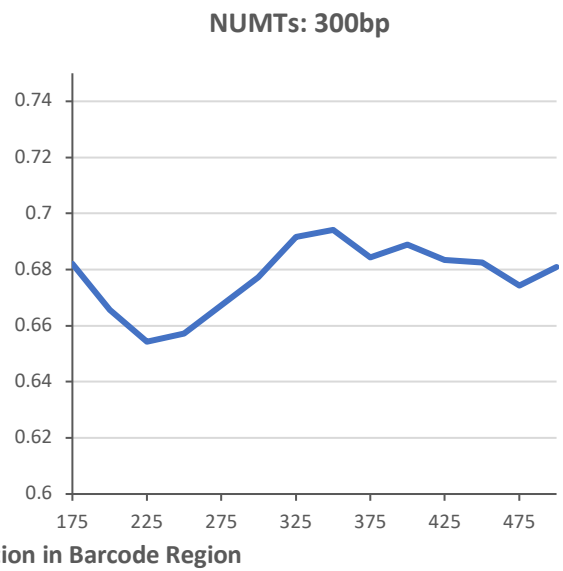

Supplement: S13 Fig — Data was obtained using a sliding window analysis across the 658 bp barcode region using RESL [27]. (PDF) [file pone.0286620.s013.pdf]
