# Supplementary material for: Interrogating 1000 insect genomes for NUMTs: A risk assessment for estimates of species richness
Source: PLoS One. 2023 Jun 8;18(6):e0286620. doi: 10.1371/journal.pone.0286620 (PMC10249859; doi:10.1371/journal.pone.0286620)

Coleoptera (pg 1 of 4)

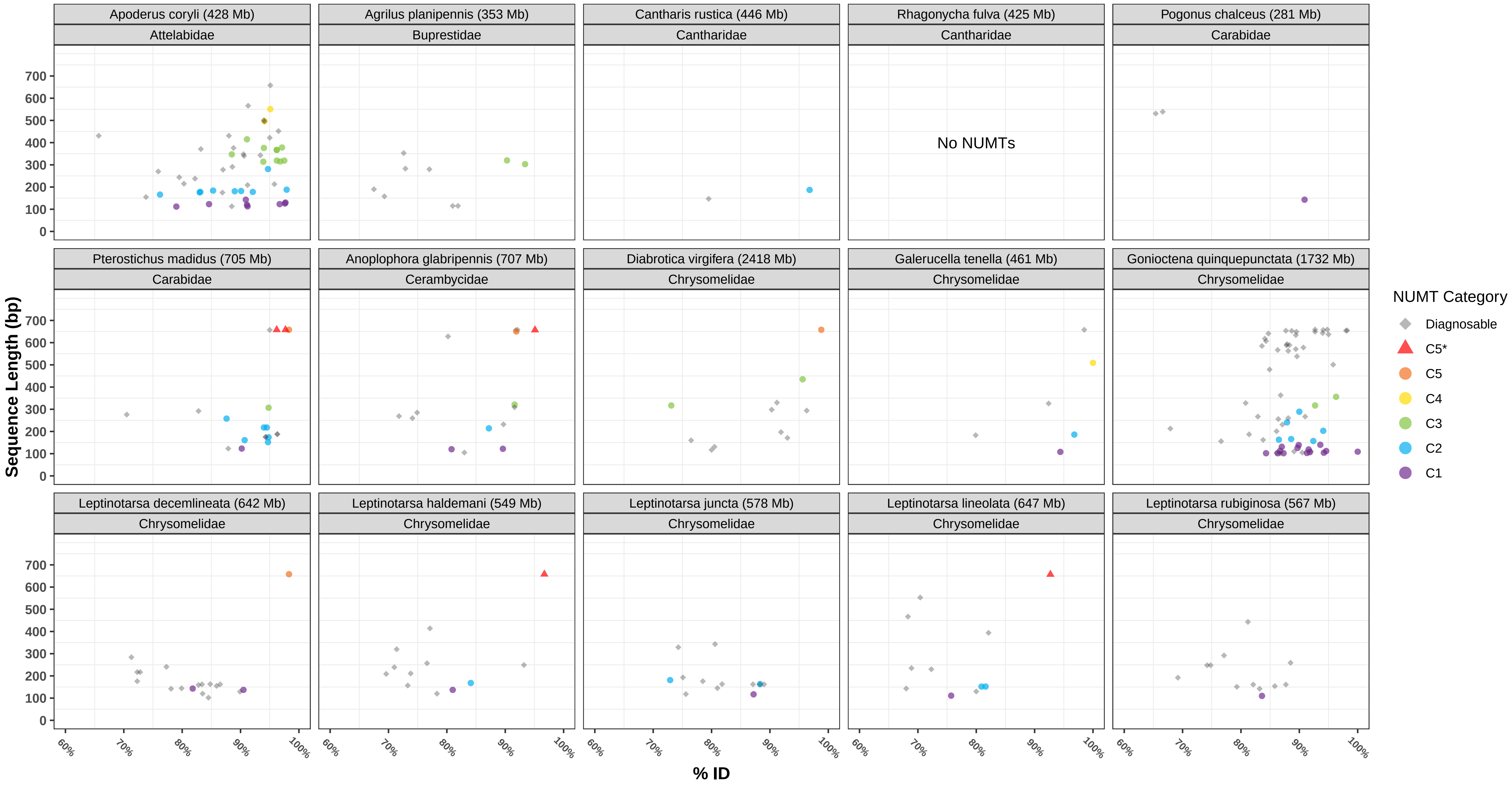

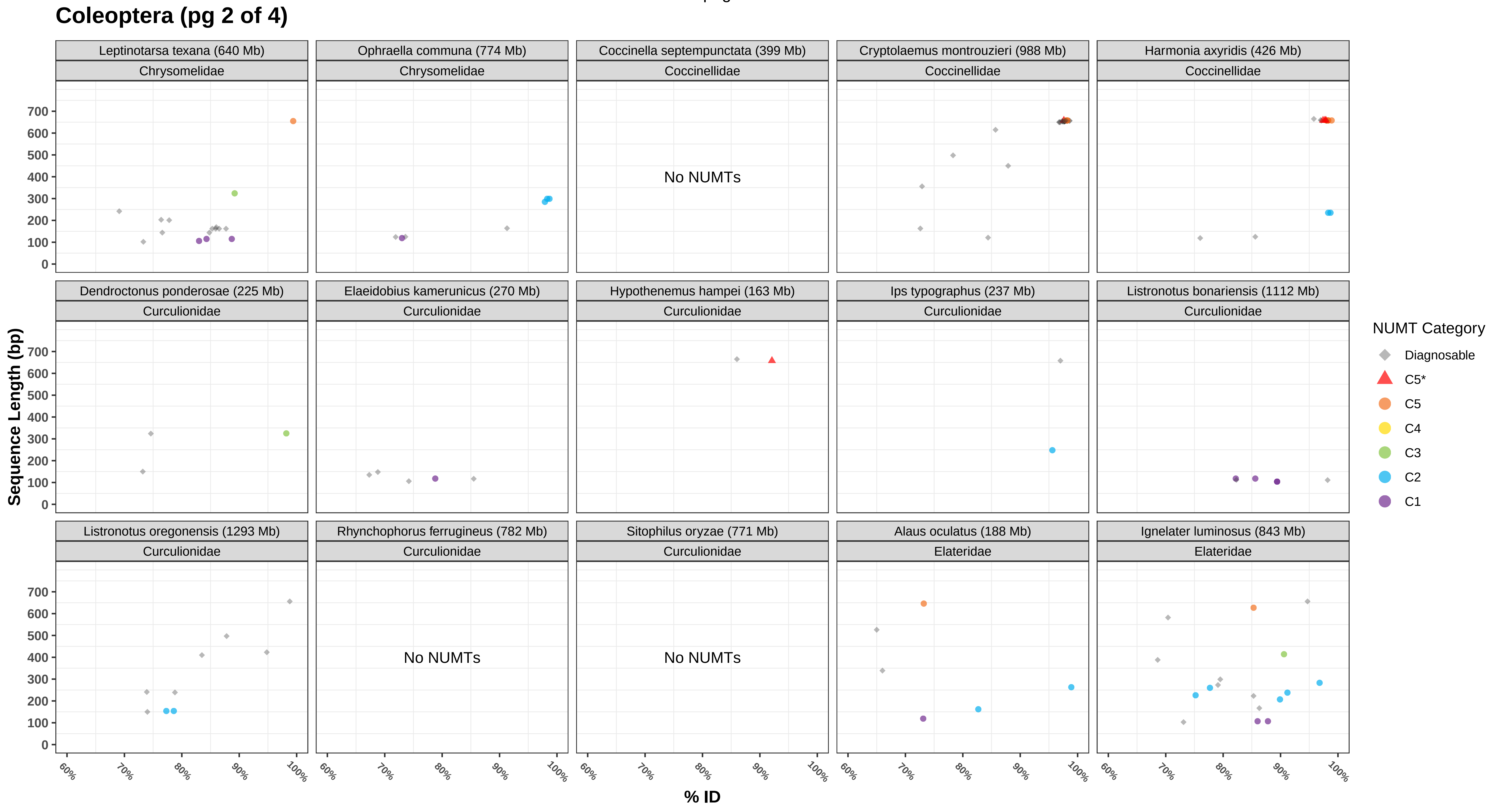
Coleoptera (pg 2 of 4)
Leptinotarsa texana (640 Mb)
Chrysomelidae
Ophraella communa (774 Mb)
Chrysomelidae
Coccinella septempunctata (399 Mb)
Coccinellidae
No NUMTs
Cryptolaemus montrouzieri (988 Mb)
Coccinellidae
Harmonia axyridis (426 Mb)
Coccinellidae
Dendroctonus ponderosae (225 Mb)
Curculionidae
Elaeidobius kamerunicus (270 Mb)
Curculionidae
Hypothenemus hampei (163 Mb)
Curculionidae
Ips typographus (237 Mb)
Curculionidae
Listronotus bonariensis (1112 Mb)
Curculionidae
Listronotus oregonensis (1293 Mb)
Curculionidae
Rhynchophorus ferrugineus (782 Mb)
Curculionidae
No NUMTs
Sitophilus oryzae (771 Mb)
Curculionidae
No NUMTs
Alaus oculatus (188 Mb)
Elateridae
Ignelater luminosus (843 Mb)
Elateridae
Sequence Length (bp)
0
100
200
300
400
500
600
700
% ID
60%
70%
80%
90%
100%
NUMT Category
Diagnosable
C5*
C5
C4
C3
C2
C1

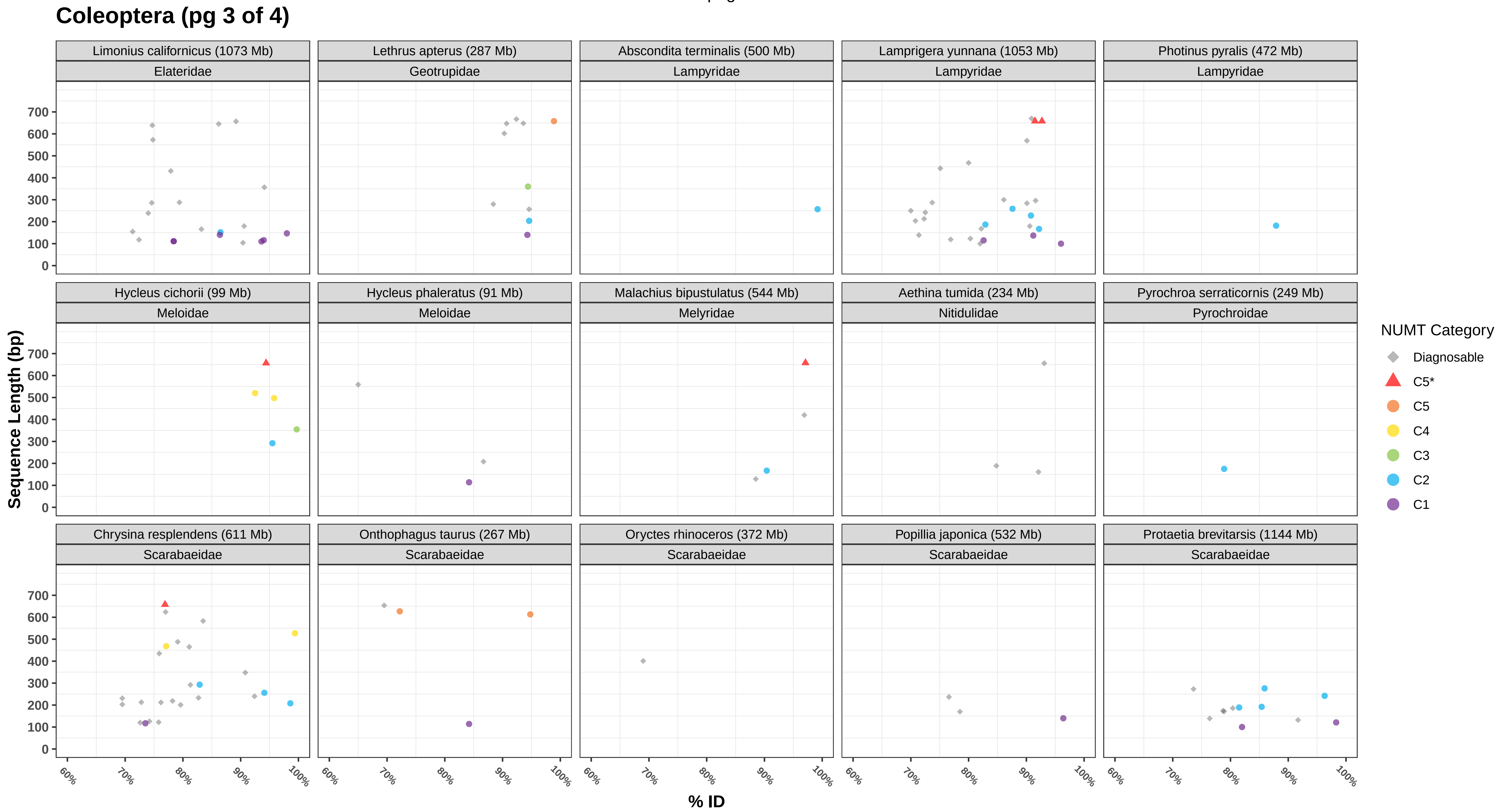
Coleoptera (pg 3 of 4)
Limonius californicus (1073 Mb)
Elateridae
Lethrus apterus (287 Mb)
Geotrupidae
Abscondita terminalis (500 Mb)
Lampyridae
Lamprigera yunnana (1053 Mb)
Lampyridae
Photinus pyralis (472 Mb)
Lampyridae
Hycleus cichorii (99 Mb)
Meloidae
Hycleus phaleratus (91 Mb)
Meloidae
Malachius bipustulatus (544 Mb)
Melyridae
Aethina tumida (234 Mb)
Nitidulidae
Pyrochroa serraticornis (249 Mb)
Pyrochroidae
Chrysina resplendens (611 Mb)
Scarabaeidae
Onthophagus taurus (267 Mb)
Scarabaeidae
Oryctes rhinoceros (372 Mb)
Scarabaeidae
Popillia japonica (532 Mb)
Scarabaeidae
Protaetia brevitarsis (1144 Mb)
Scarabaeidae
Sequence Length (bp)
0
100
200
300
400
500
600
700
% ID
60%
70%
80%
90%
100%
NUMT Category
Diagnosable
C5*
C5
C4
C3
C2
C1

Coleoptera (pg 4 of 4)

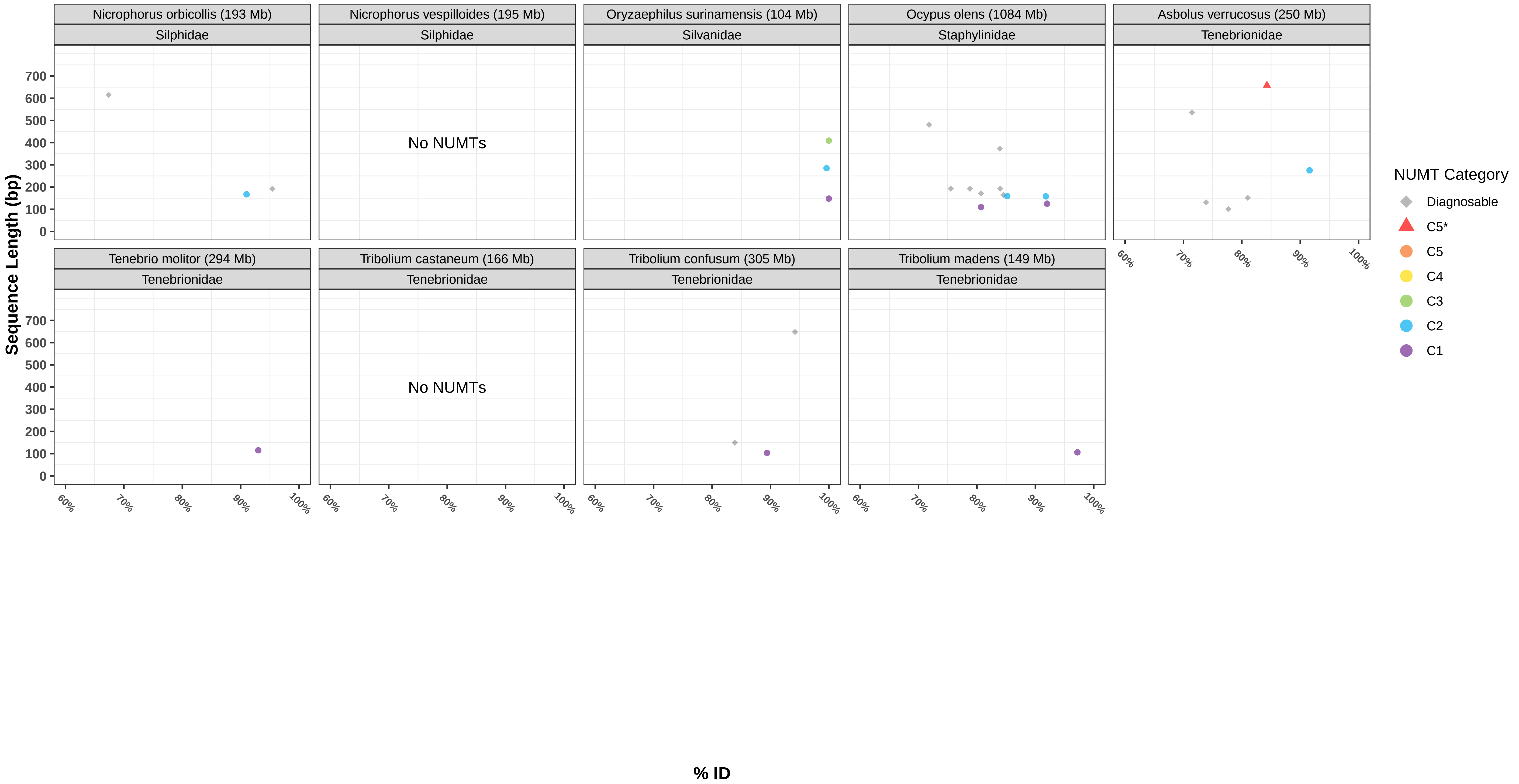

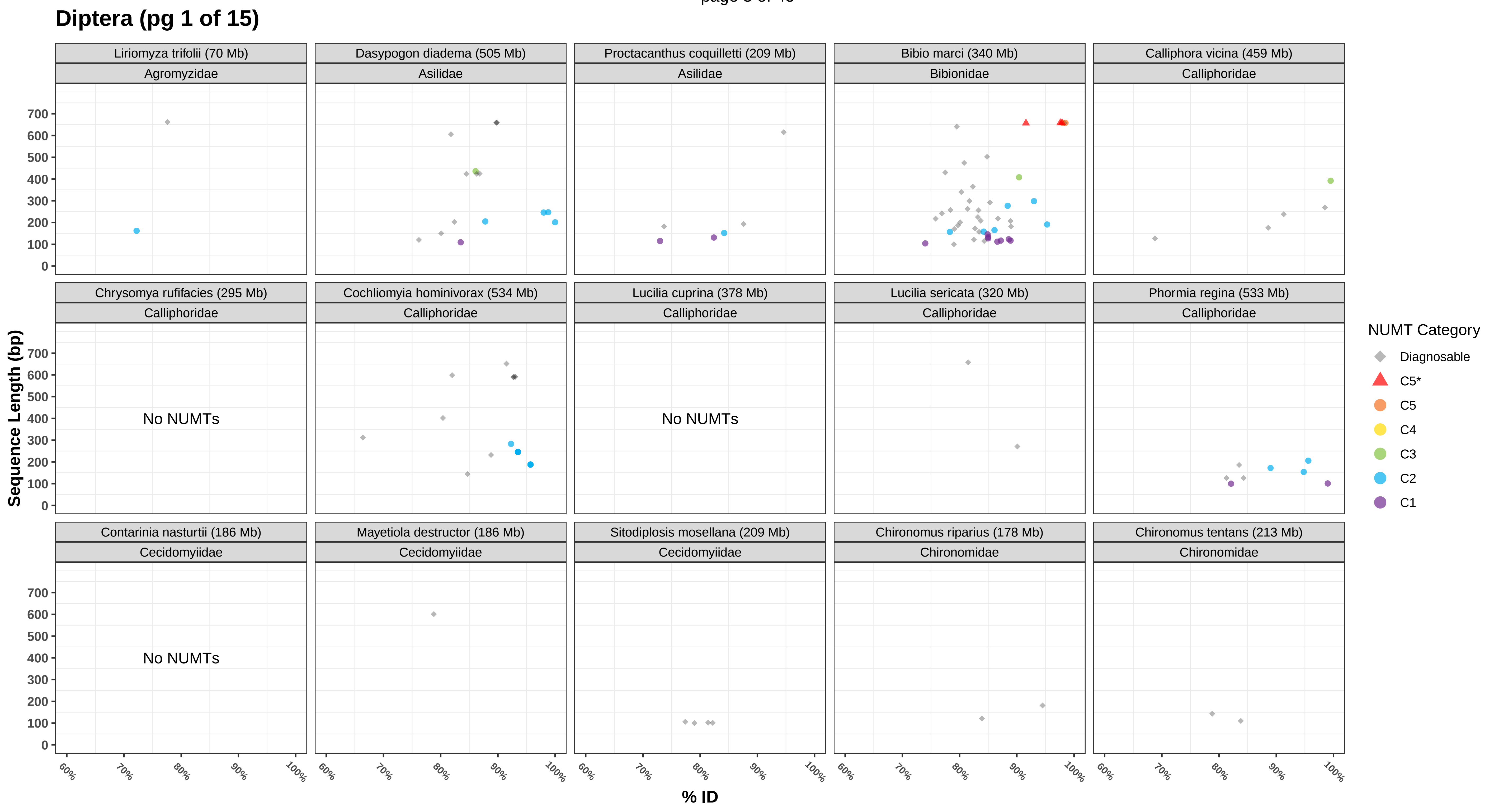
Diptera (pg 1 of 15)
Liriomyza trifolii (70 Mb)
Agromyzidae
Dasypogon diadema (505 Mb)
Asilidae
Proctacanthus coquilletti (209 Mb)
Asilidae
Bibio marci (340 Mb)
Bibionidae
Calliphora vicina (459 Mb)
Calliphoridae
Chrysomya rufifacies (295 Mb)
Calliphoridae
No NUMTs
Cochliomyia hominivorax (534 Mb)
Calliphoridae
Lucilia cuprina (378 Mb)
Calliphoridae
No NUMTs
Lucilia sericata (320 Mb)
Calliphoridae
Phormia regina (533 Mb)
Calliphoridae
Contarinia nasturtii (186 Mb)
Cecidomyiidae
No NUMTs
Mayetiola destructor (186 Mb)
Cecidomyiidae
Sitodiplosis mosellana (209 Mb)
Cecidomyiidae
Chironomus riparius (178 Mb)
Chironomidae
Chironomus tentans (213 Mb)
Chironomidae
Sequence Length (bp)
0
100
200
300
400
500
600
700
% ID
60%
70%
80%
90%
100%
NUMT Category
Diagnosable
C5*
C5
C4
C3
C2
C1

Diptera (pg 2 of 15)

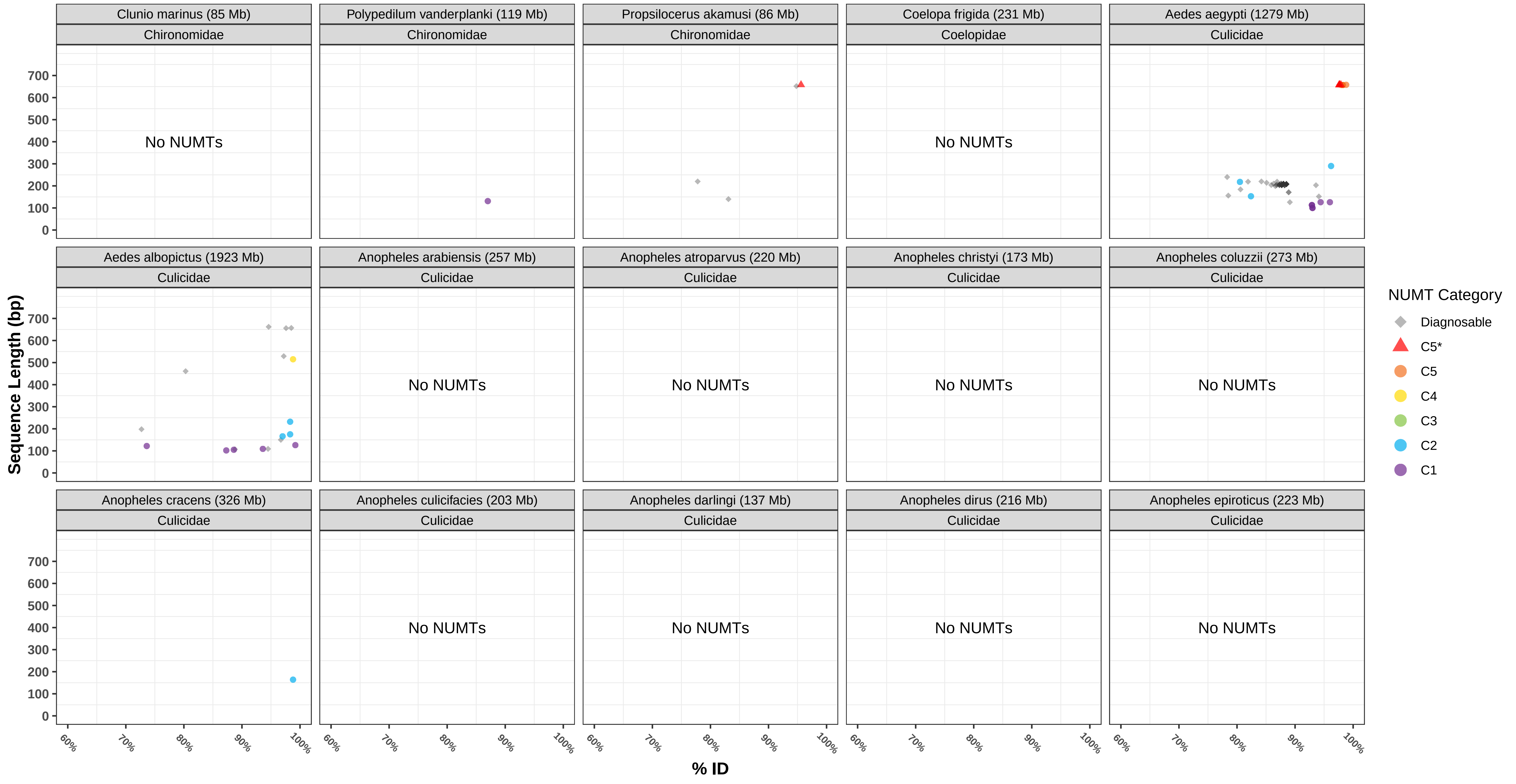

Diptera (pg 3 of 15)

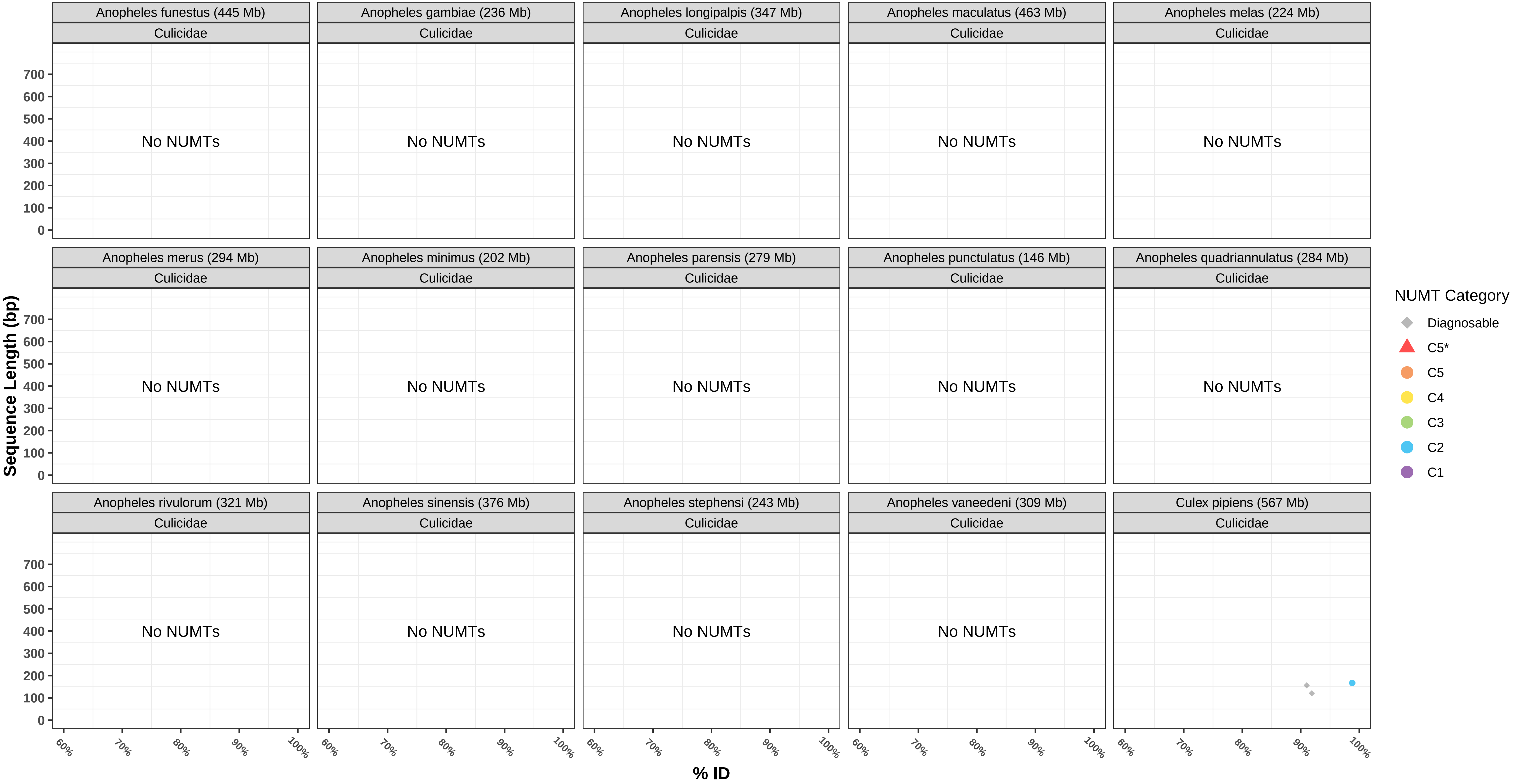

Diptera (pg 4 of 15)

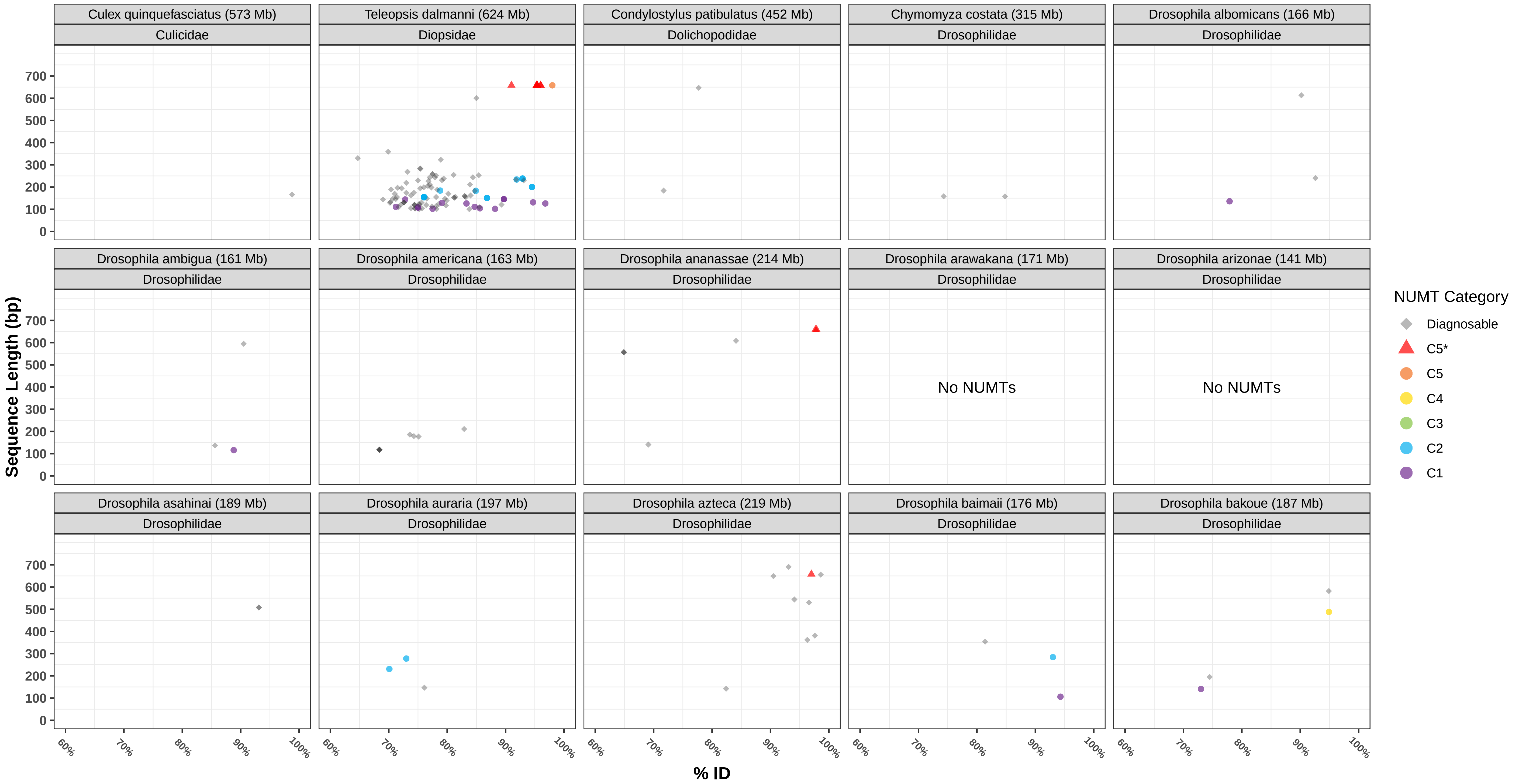

Diptera (pg 5 of 15)

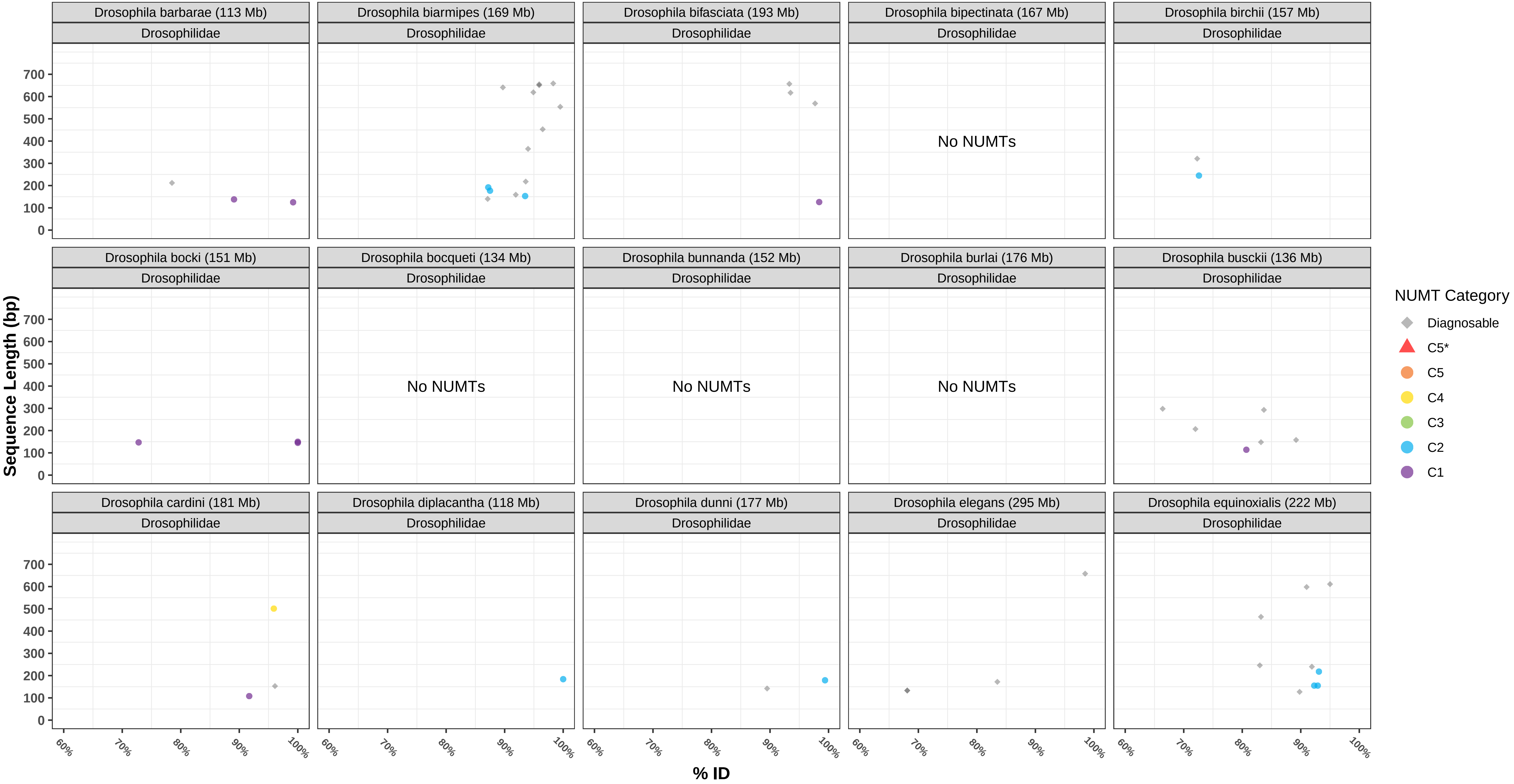

Diptera (pg 6 of 15)

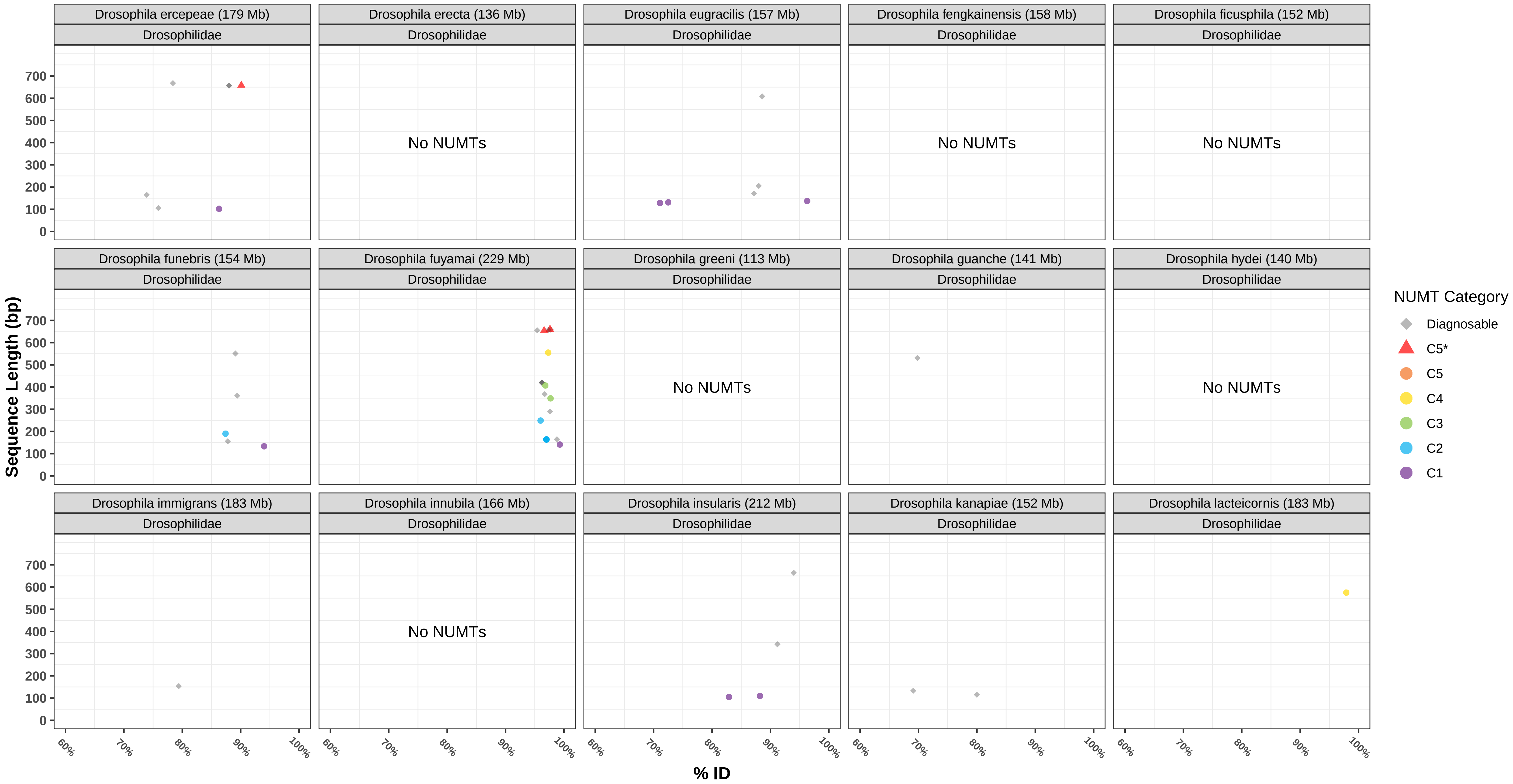

Diptera (pg 7 of 15)

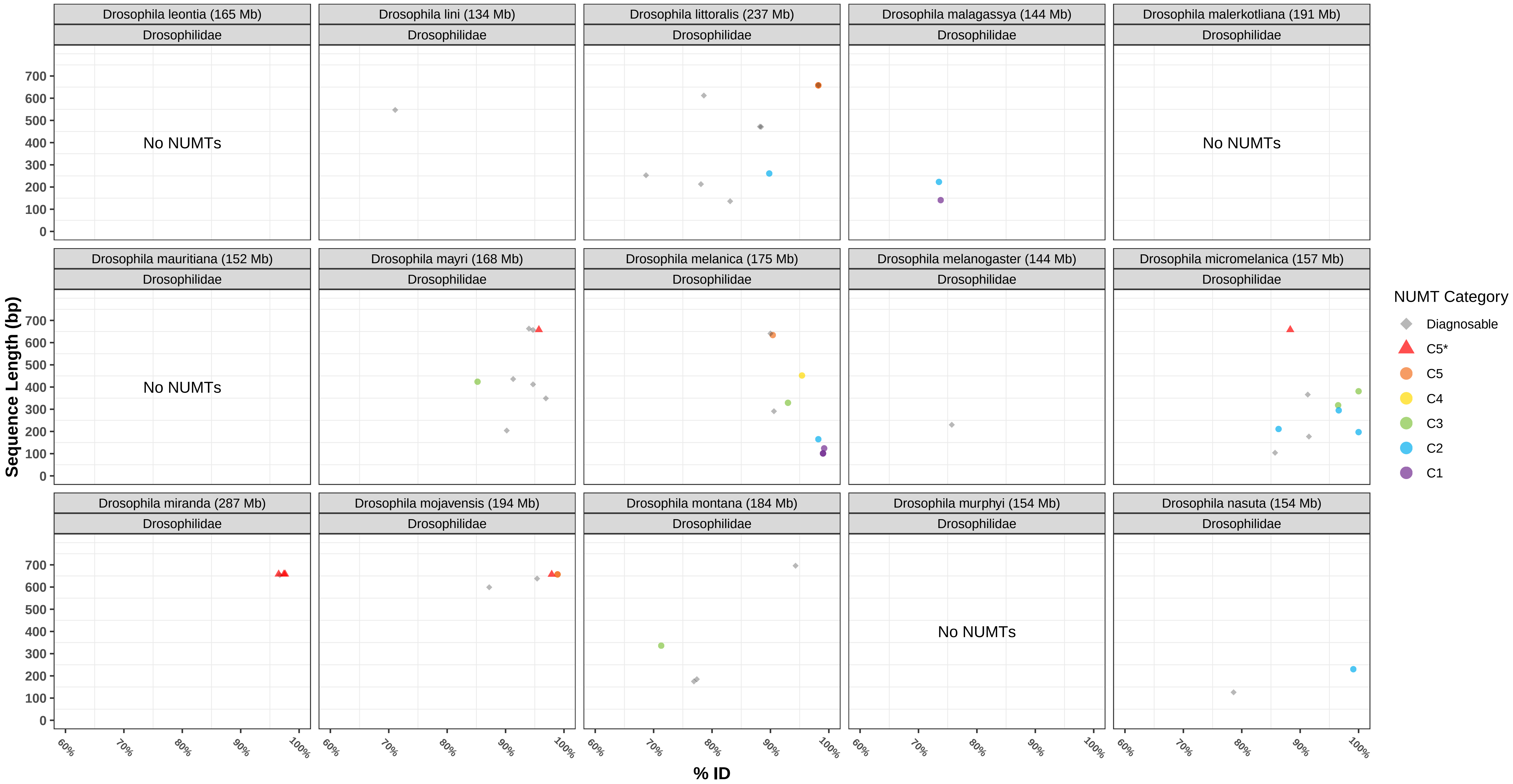

Diptera (pg 8 of 15)

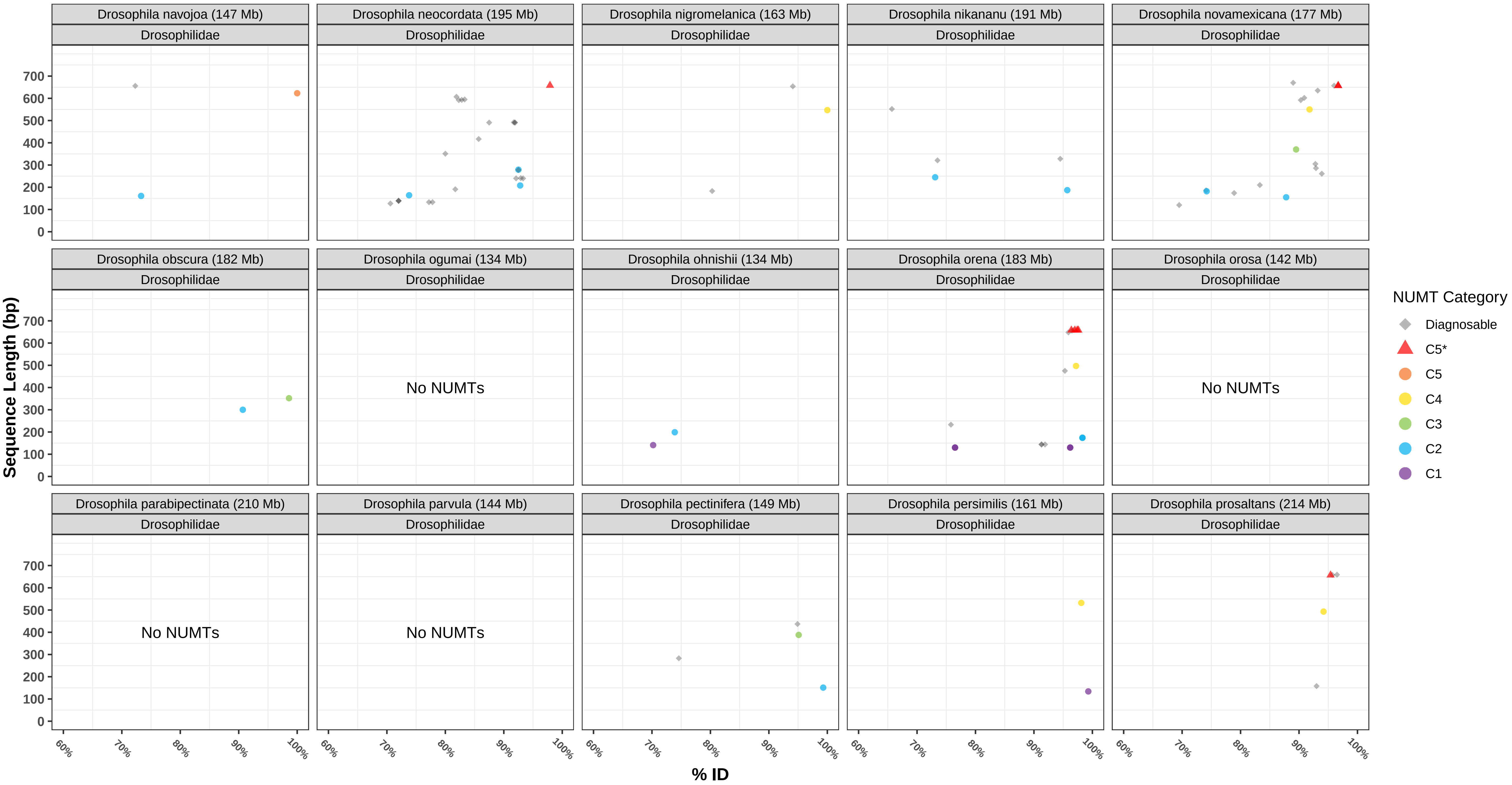

Diptera (pg 9 of 15)

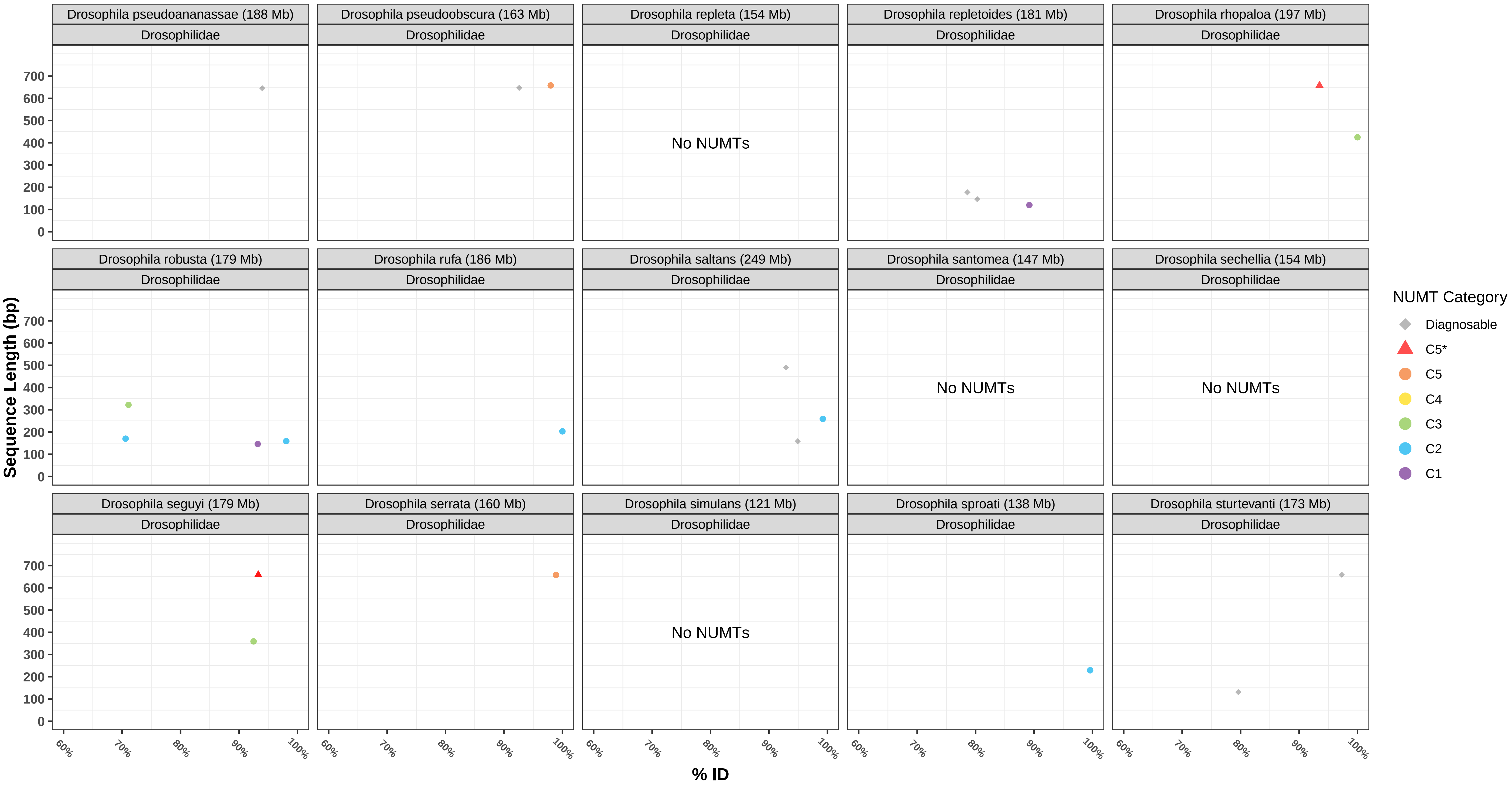

Diptera (pg 10 of 15)

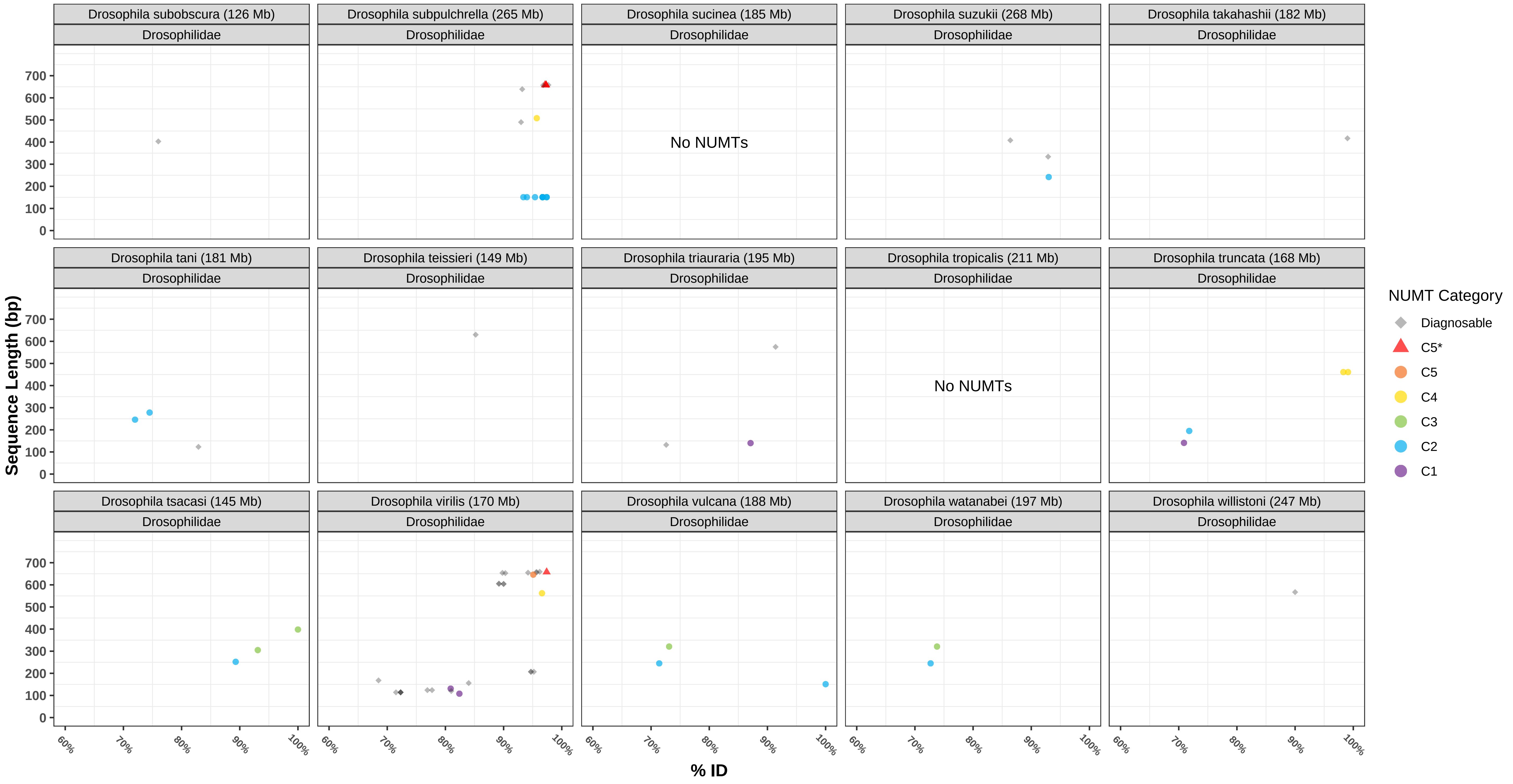

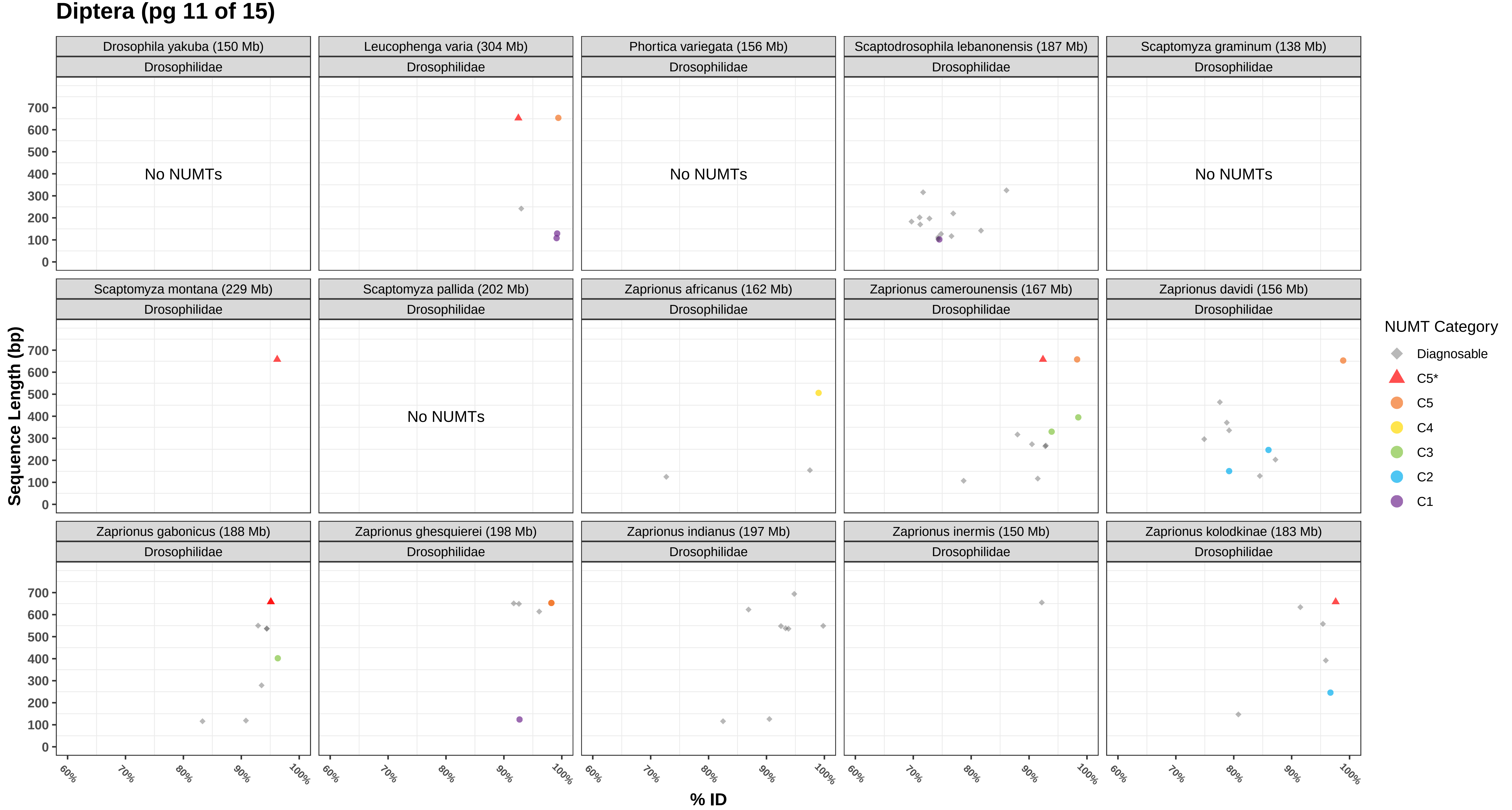

Diptera (pg 11 of 15)
Drosophila yakuba (150 Mb)
Drosophilidae
No NUMTs
Leucophenga varia (304 Mb)
Drosophilidae
Phortica variegata (156 Mb)
Drosophilidae
No NUMTs
Scaptodrosophila lebanonensis (187 Mb)
Drosophilidae
Scaptomyza graminum (138 Mb)
Drosophilidae
No NUMTs
Scaptomyza montana (229 Mb)
Drosophilidae
Scaptomyza pallida (202 Mb)
Drosophilidae
No NUMTs
Zaprionus africanus (162 Mb)
Drosophilidae
Zaprionus camerounensis (167 Mb)
Drosophilidae
Zaprionus davidi (156 Mb)
Drosophilidae
Zaprionus gabonicus (188 Mb)
Drosophilidae
Zaprionus ghesquierei (198 Mb)
Drosophilidae
Zaprionus indianus (197 Mb)
Drosophilidae
Zaprionus inermis (150 Mb)
Drosophilidae
Zaprionus kolodkinae (183 Mb)
Drosophilidae
Sequence Length (bp)
0
100
200
300
400
500
600
700
% ID
60%
70%
80%
90%
100%
NUMT Category
Diagnosable
C5*
C5
C4
C3
C2
C1

Diptera (pg 12 of 15)

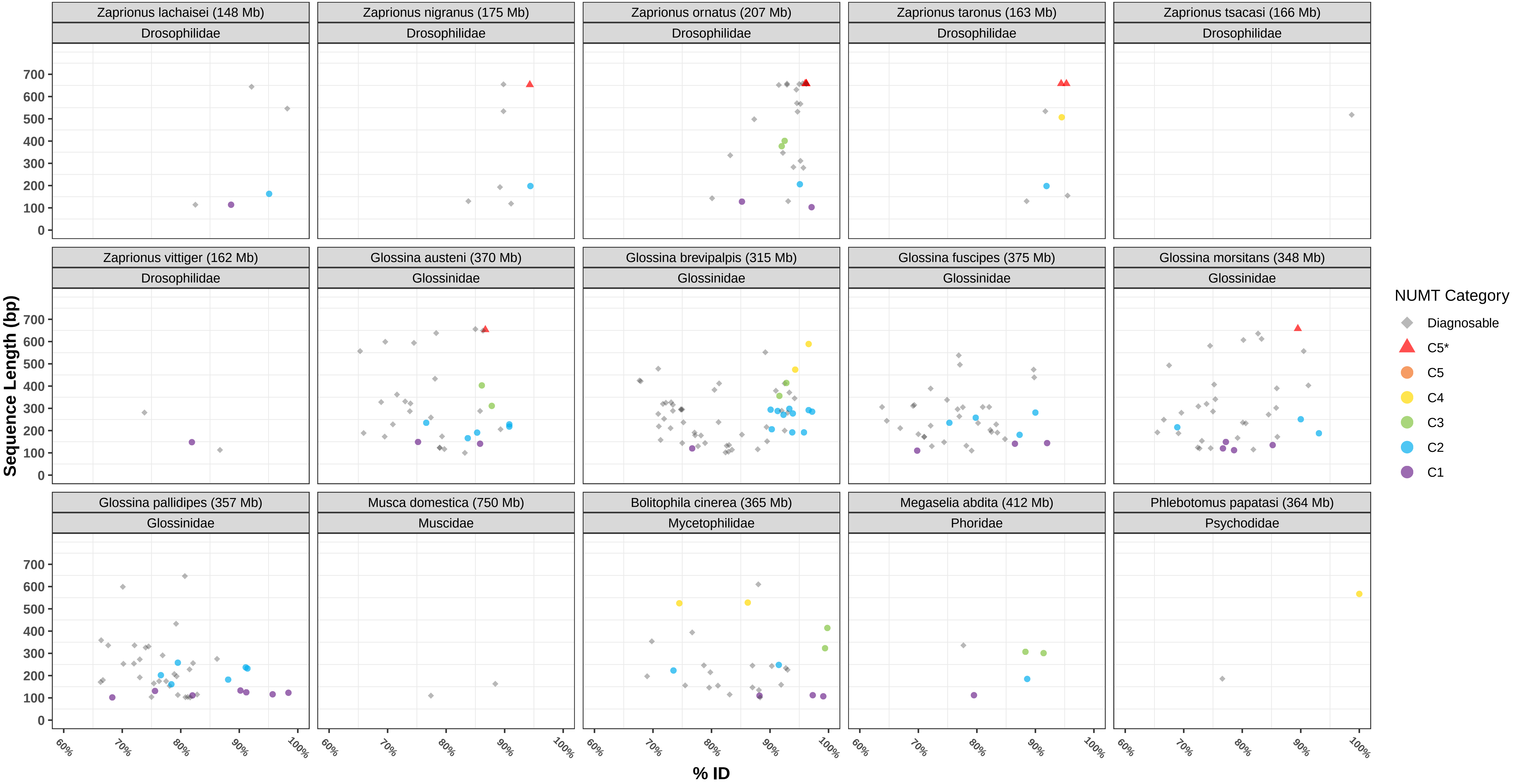

Diptera (pg 13 of 15)

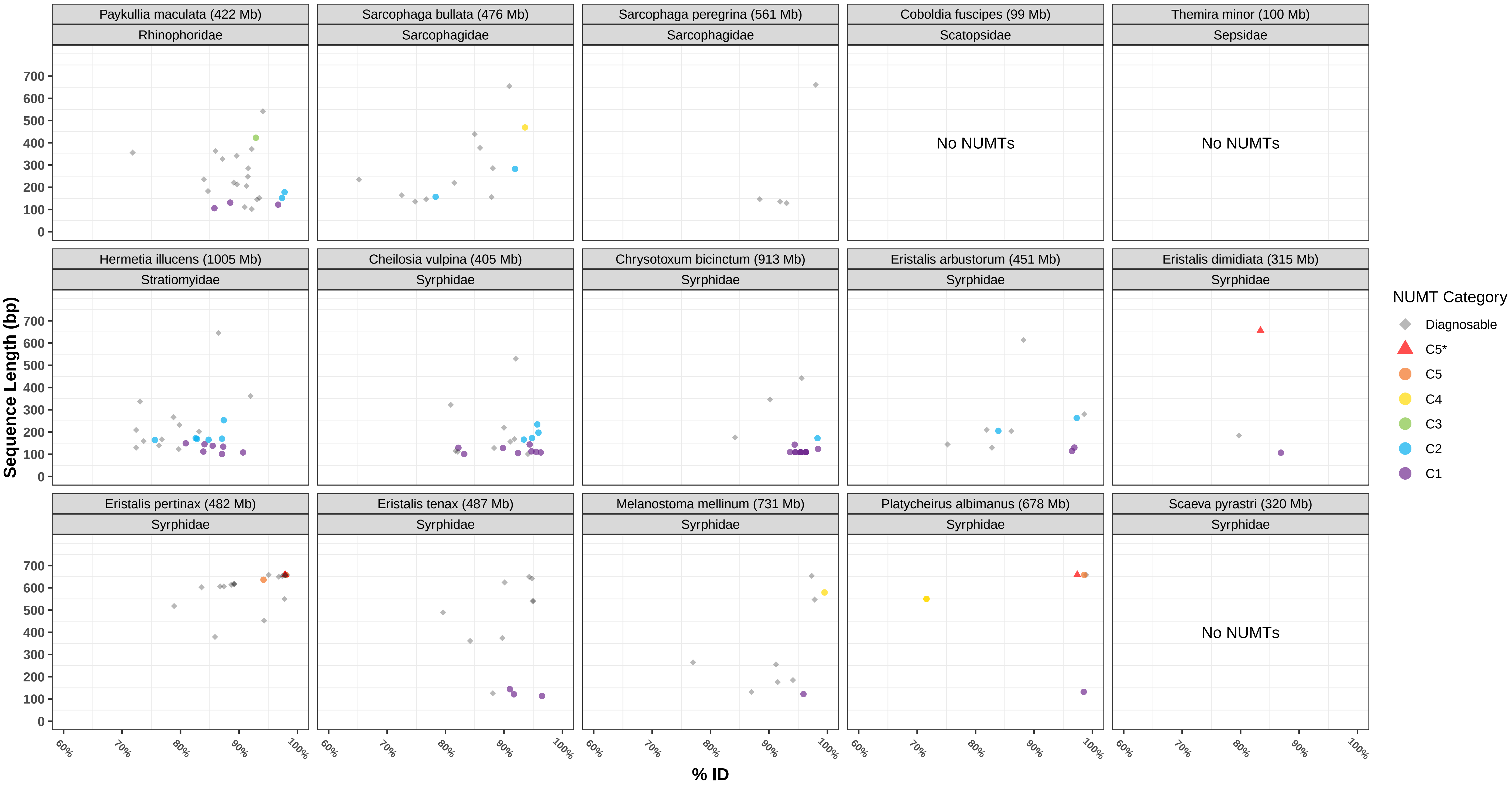

Diptera (pg 14 of 15)

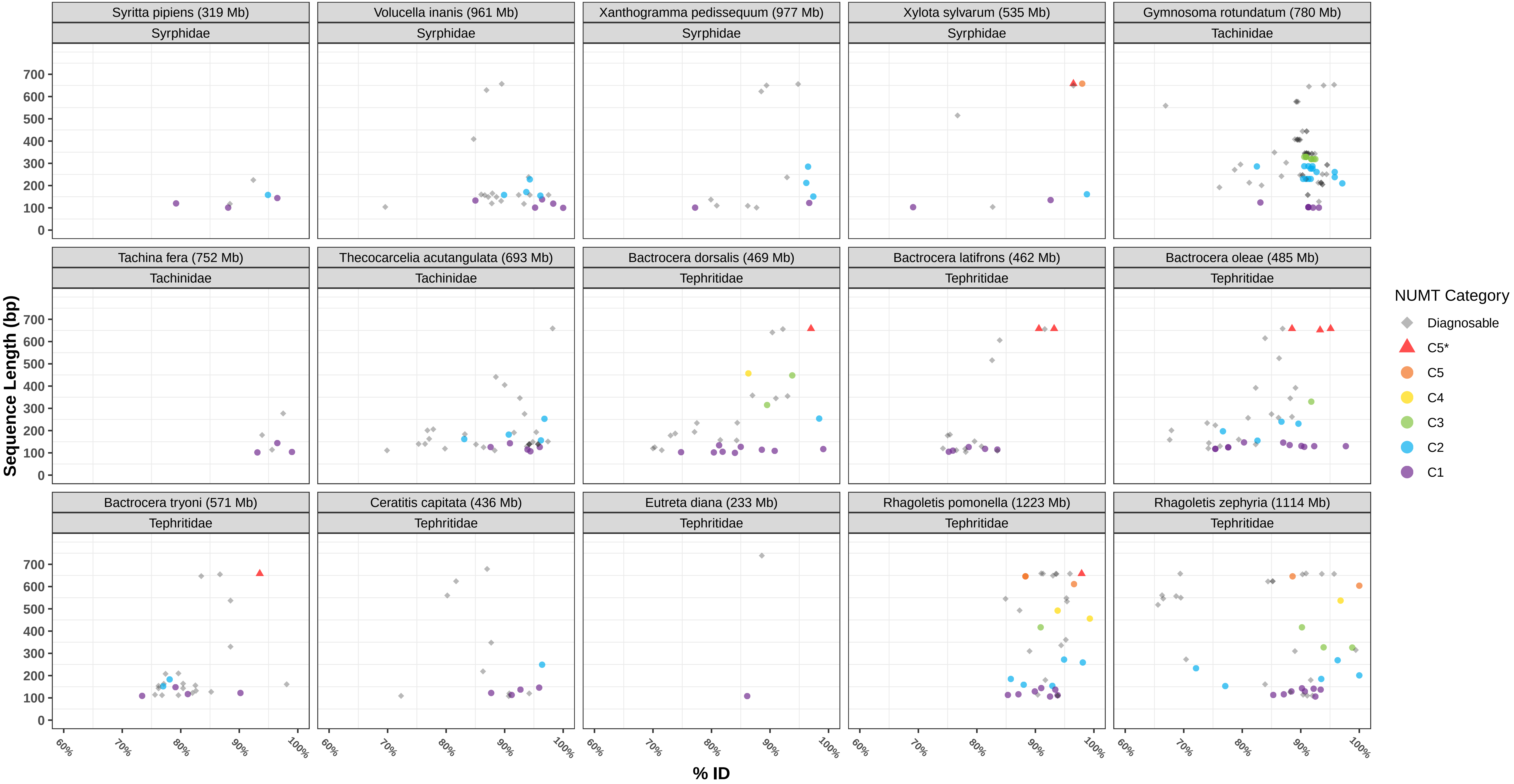

Diptera (pg 15 of 15)

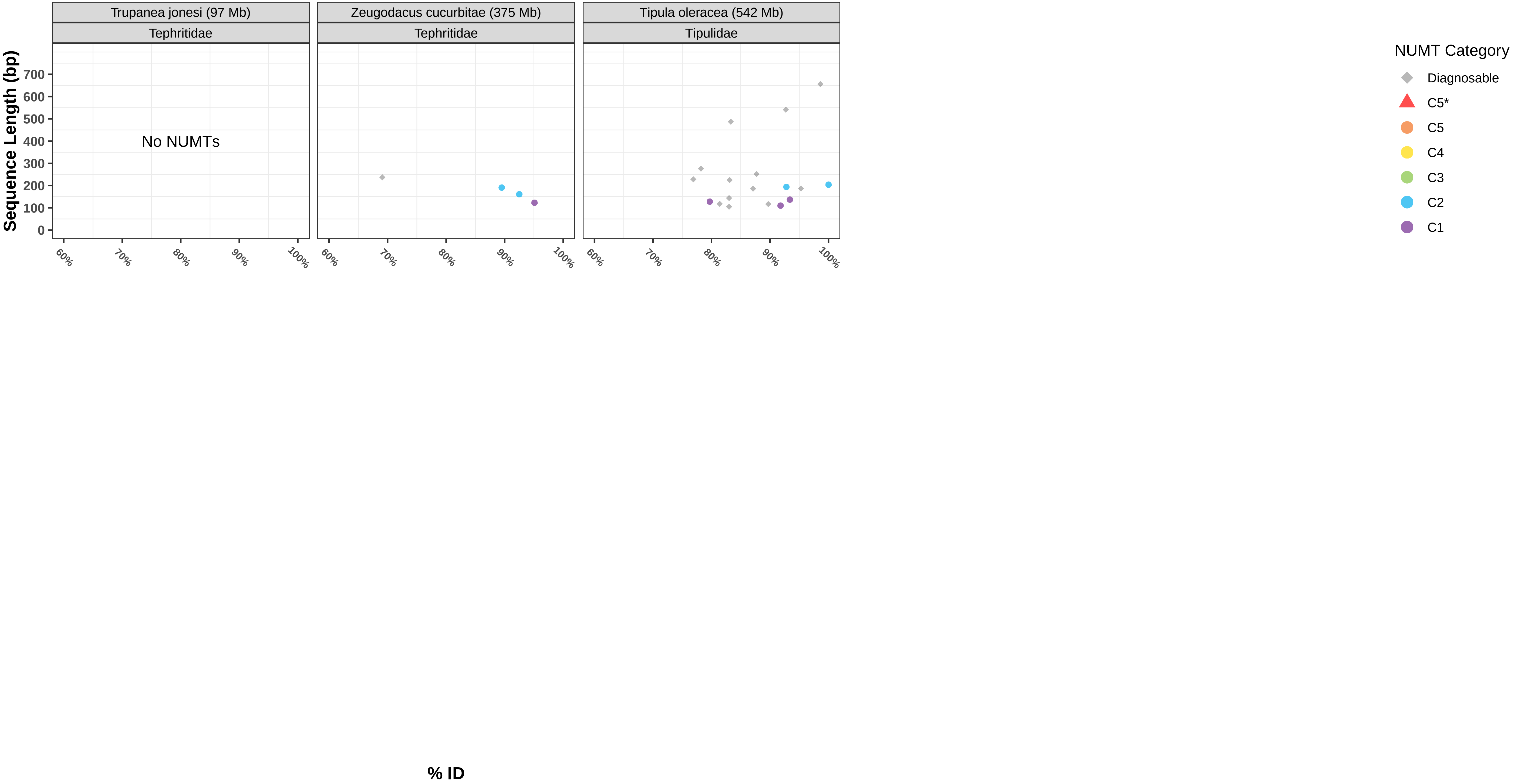

Hemiptera (pg 1 of 4)

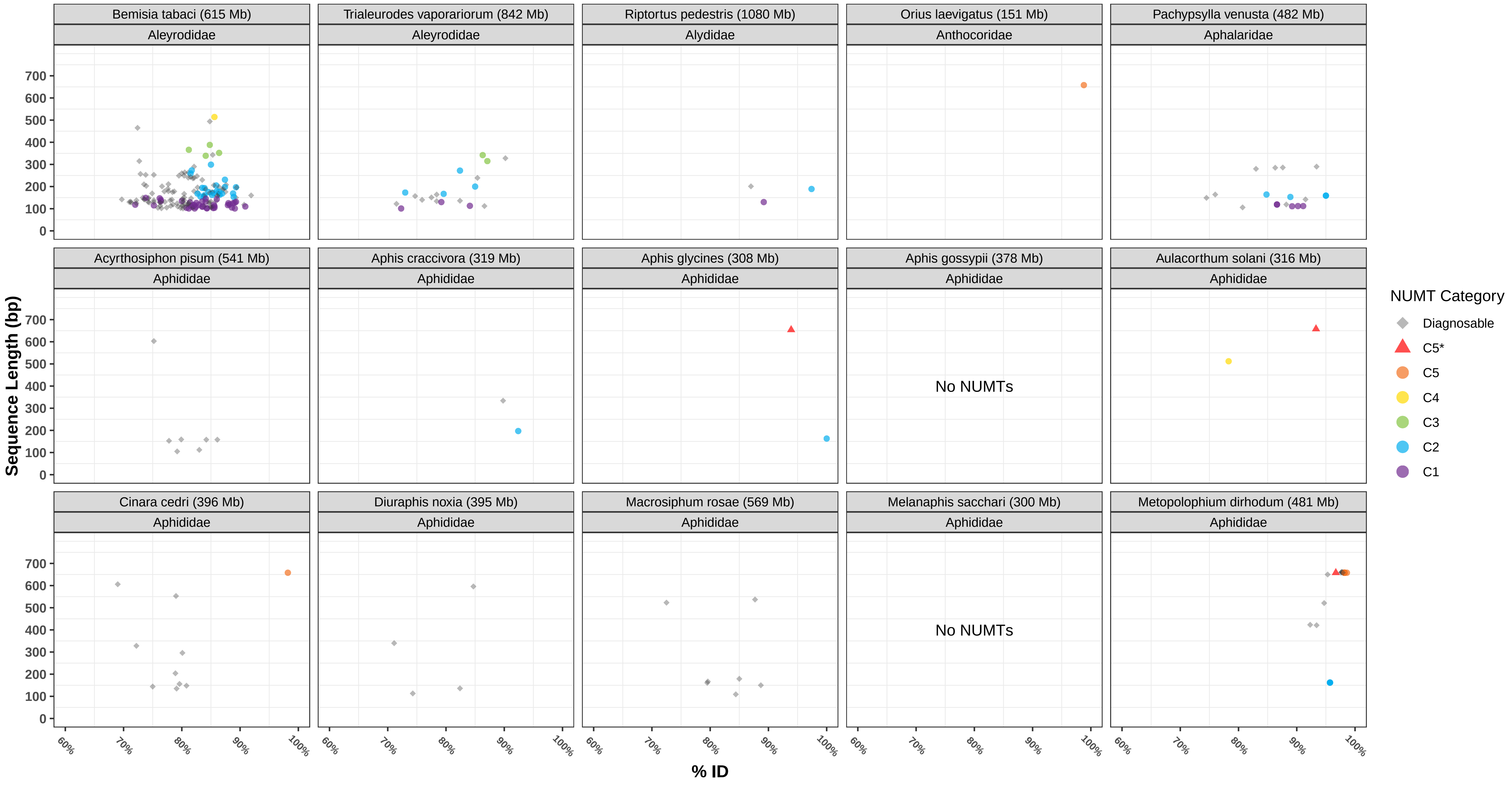

Hemiptera (pg 2 of 4)

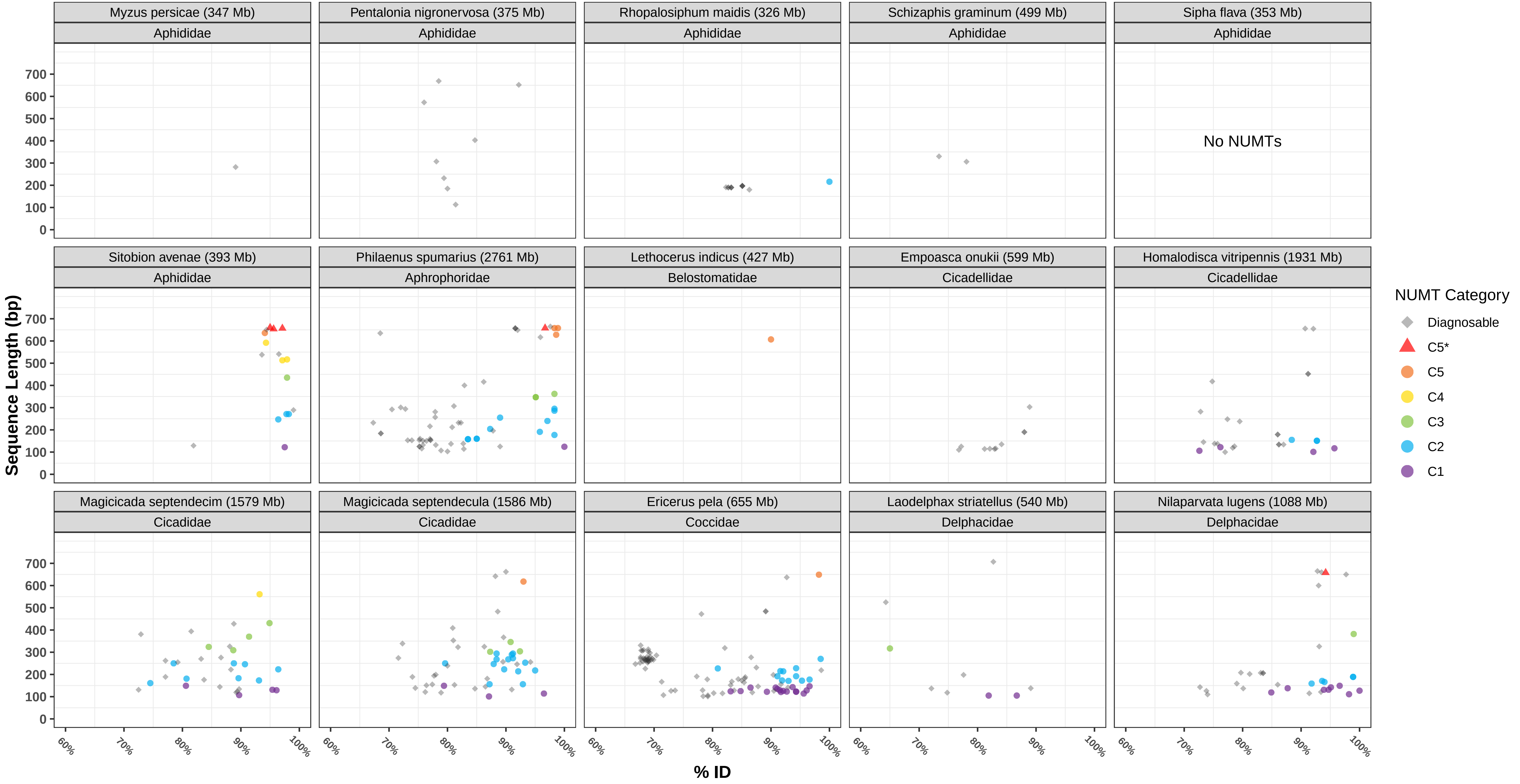

Hemiptera (pg 3 of 4)

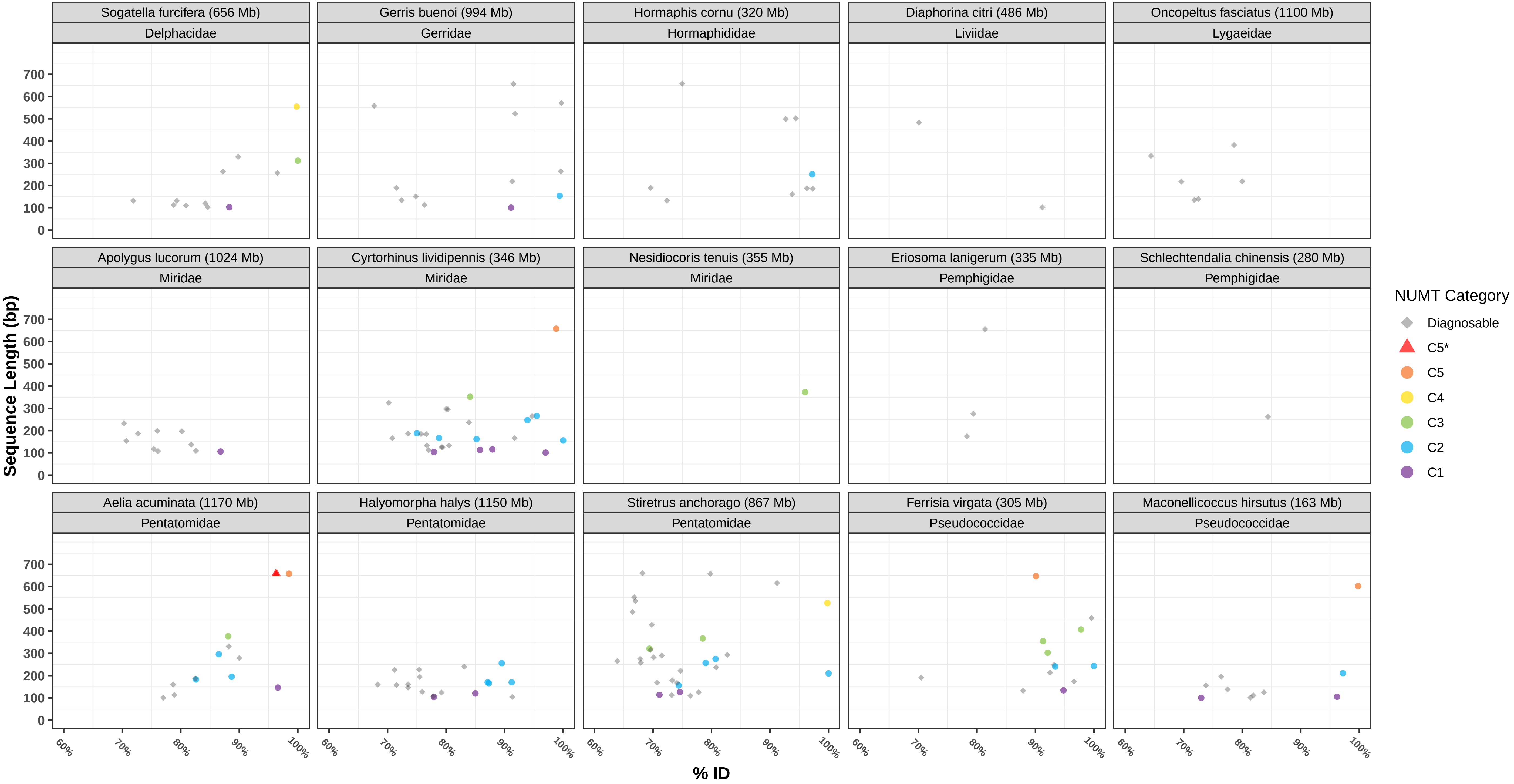

Hemiptera (pg 4 of 4)

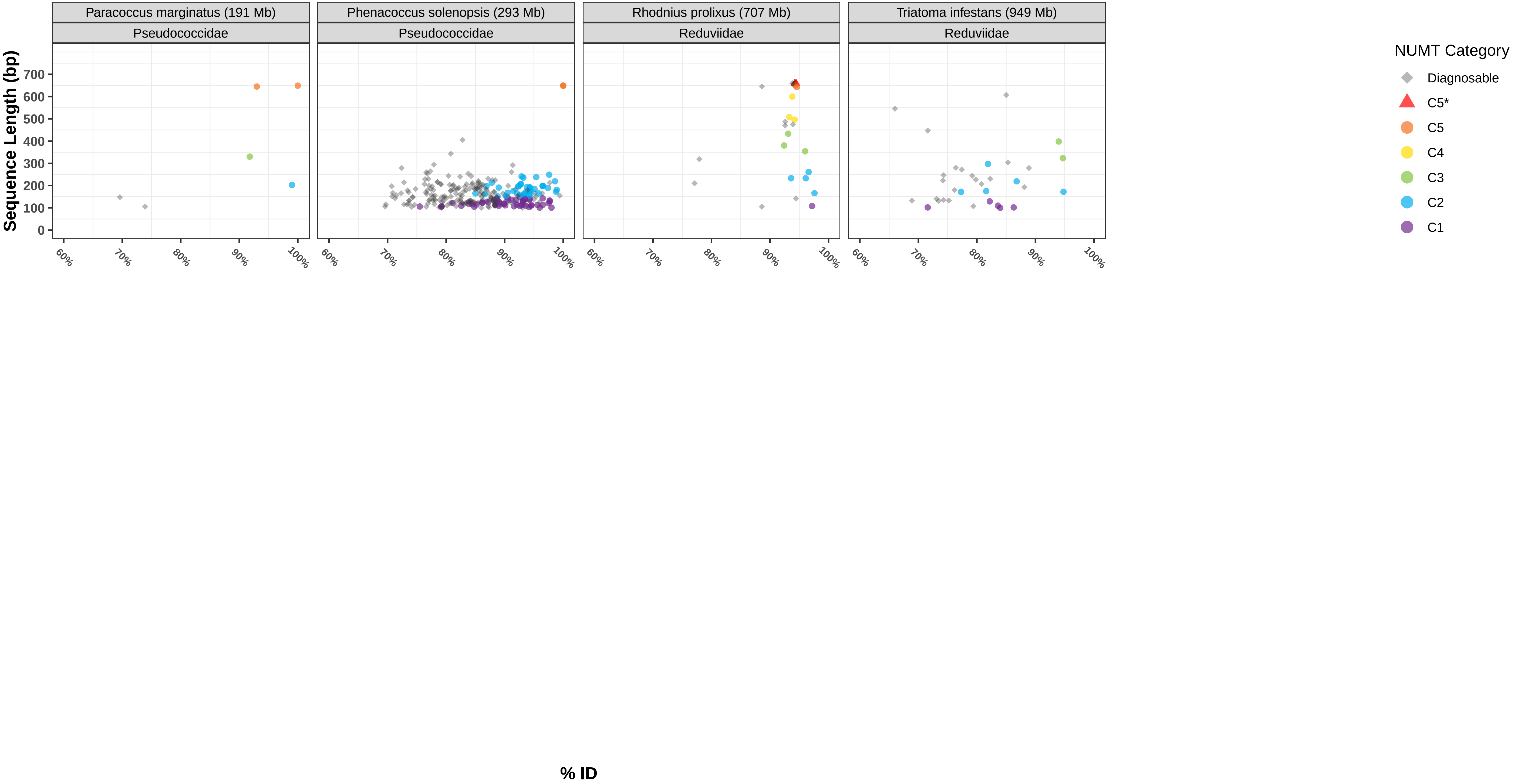

Hymenoptera (pg 1 of 9)

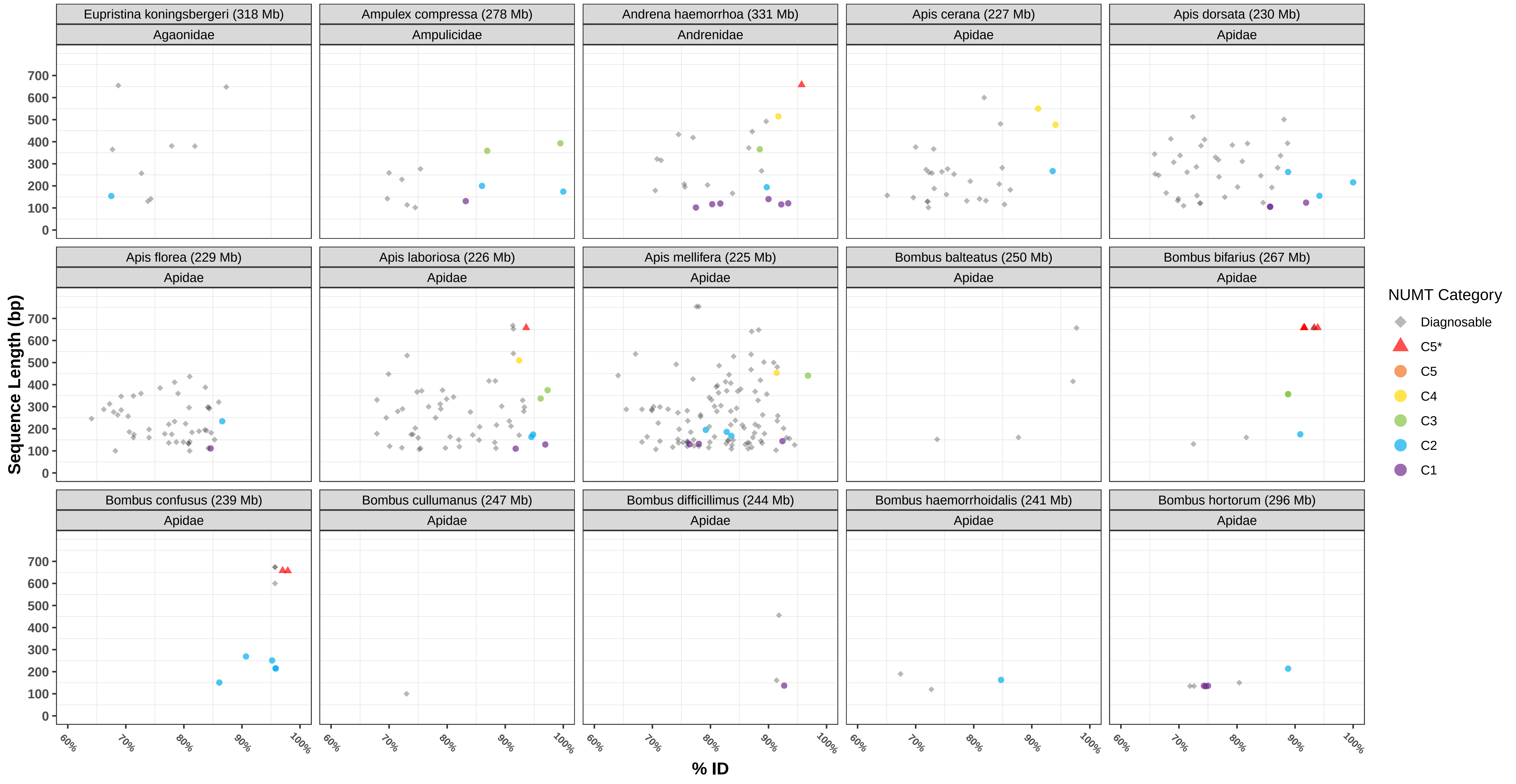

Hymenoptera (pg 2 of 9)

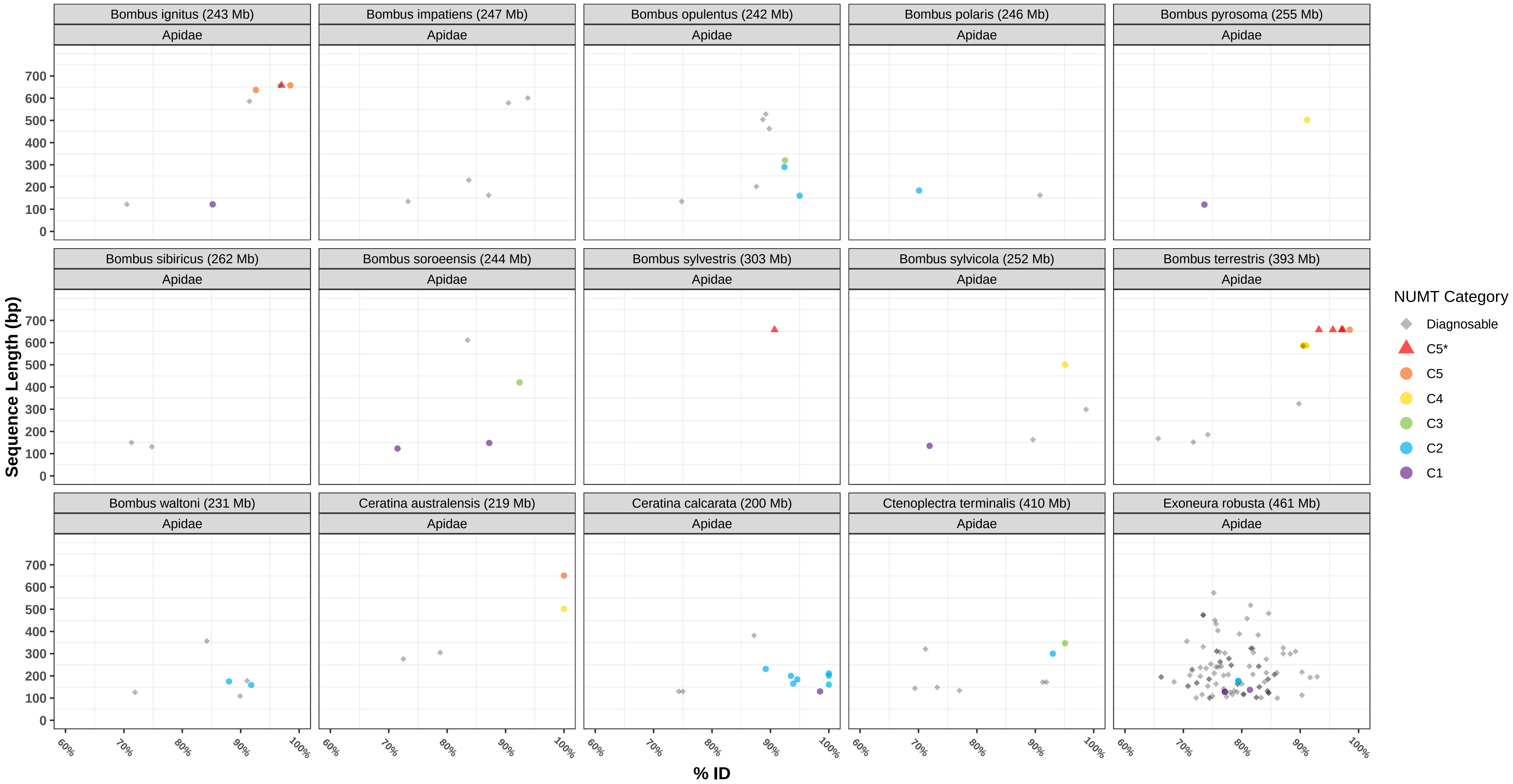

Hymenoptera (pg 3 of 9)

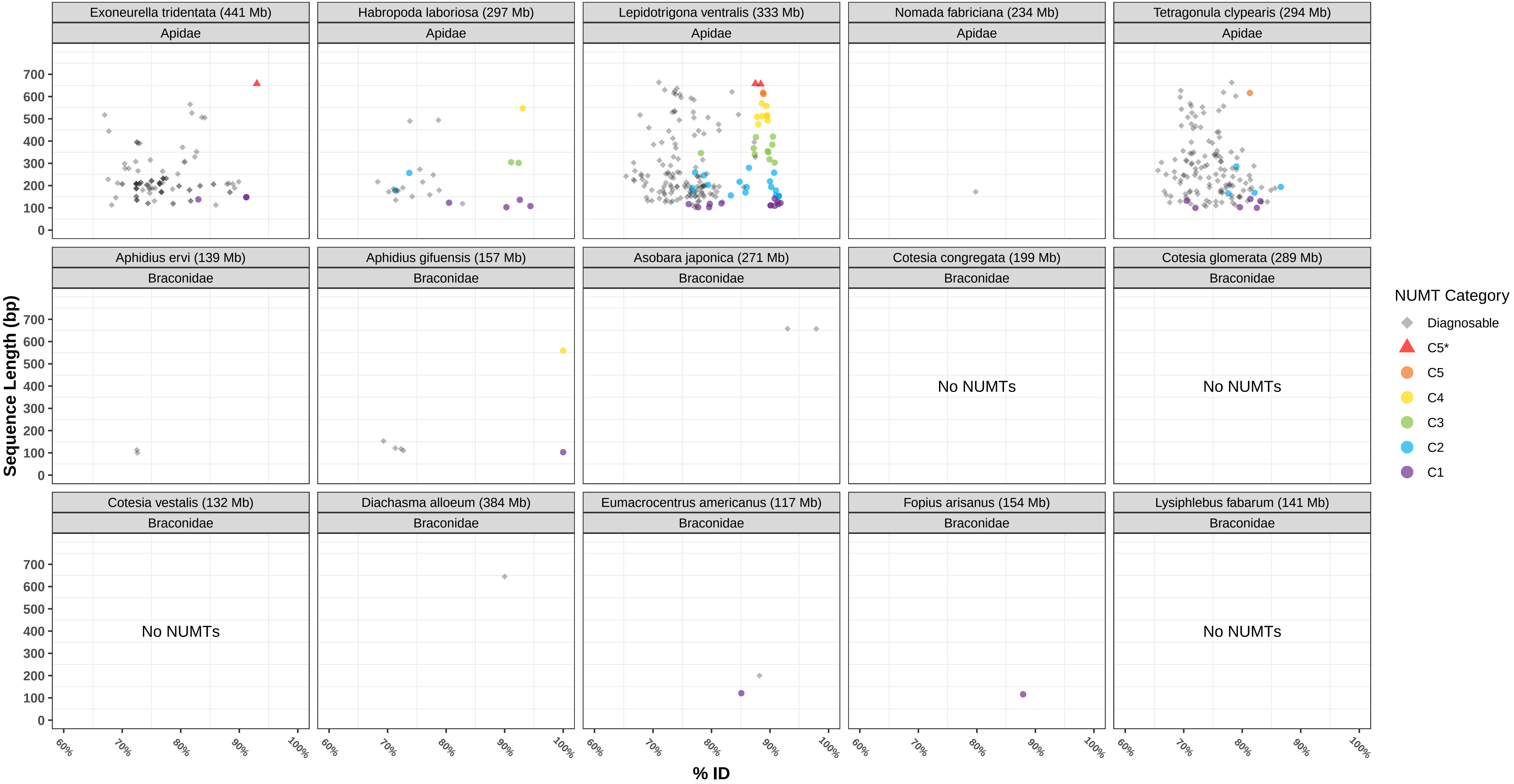

Hymenoptera (pg 4 of 9)

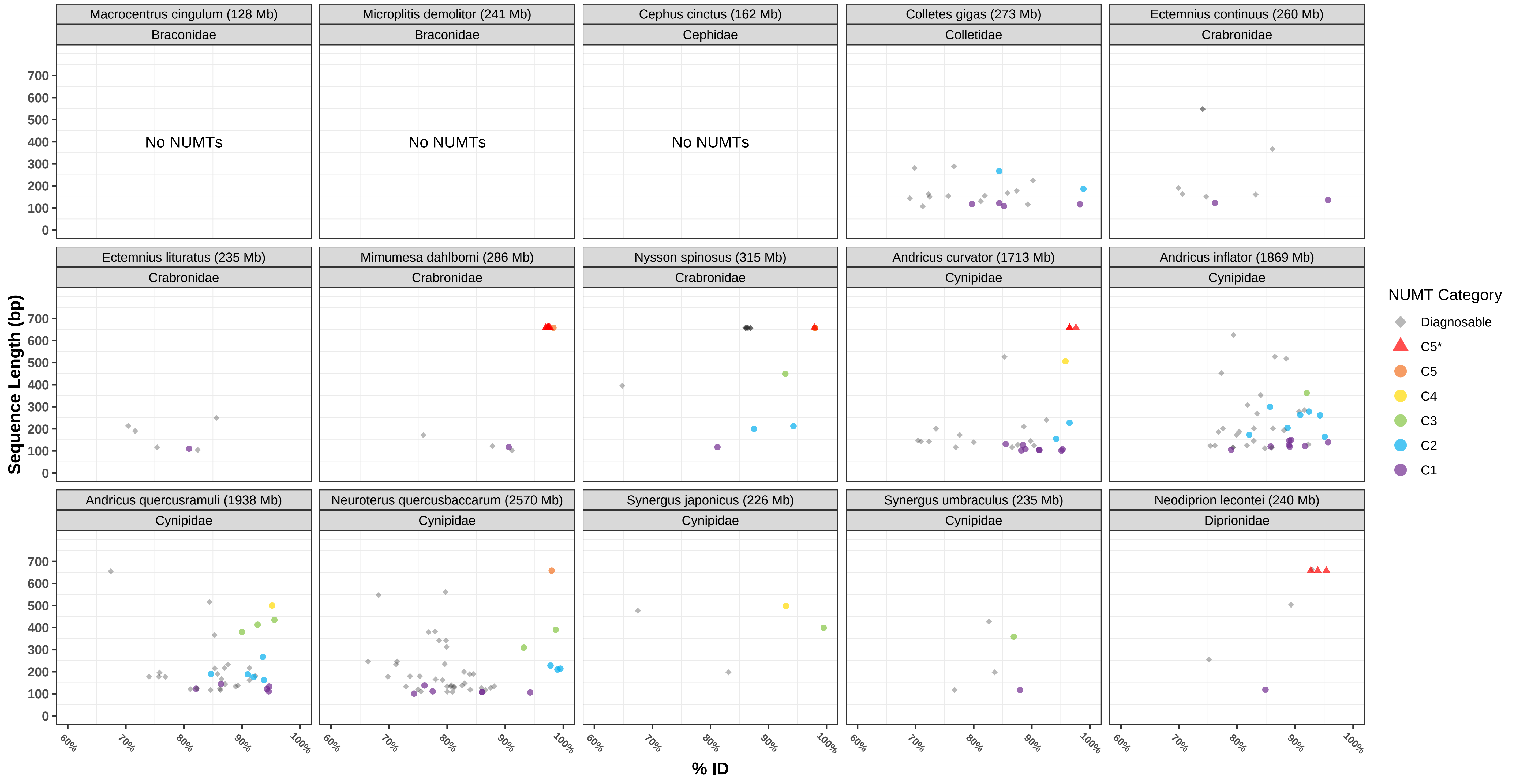

Hymenoptera (pg 5 of 9)

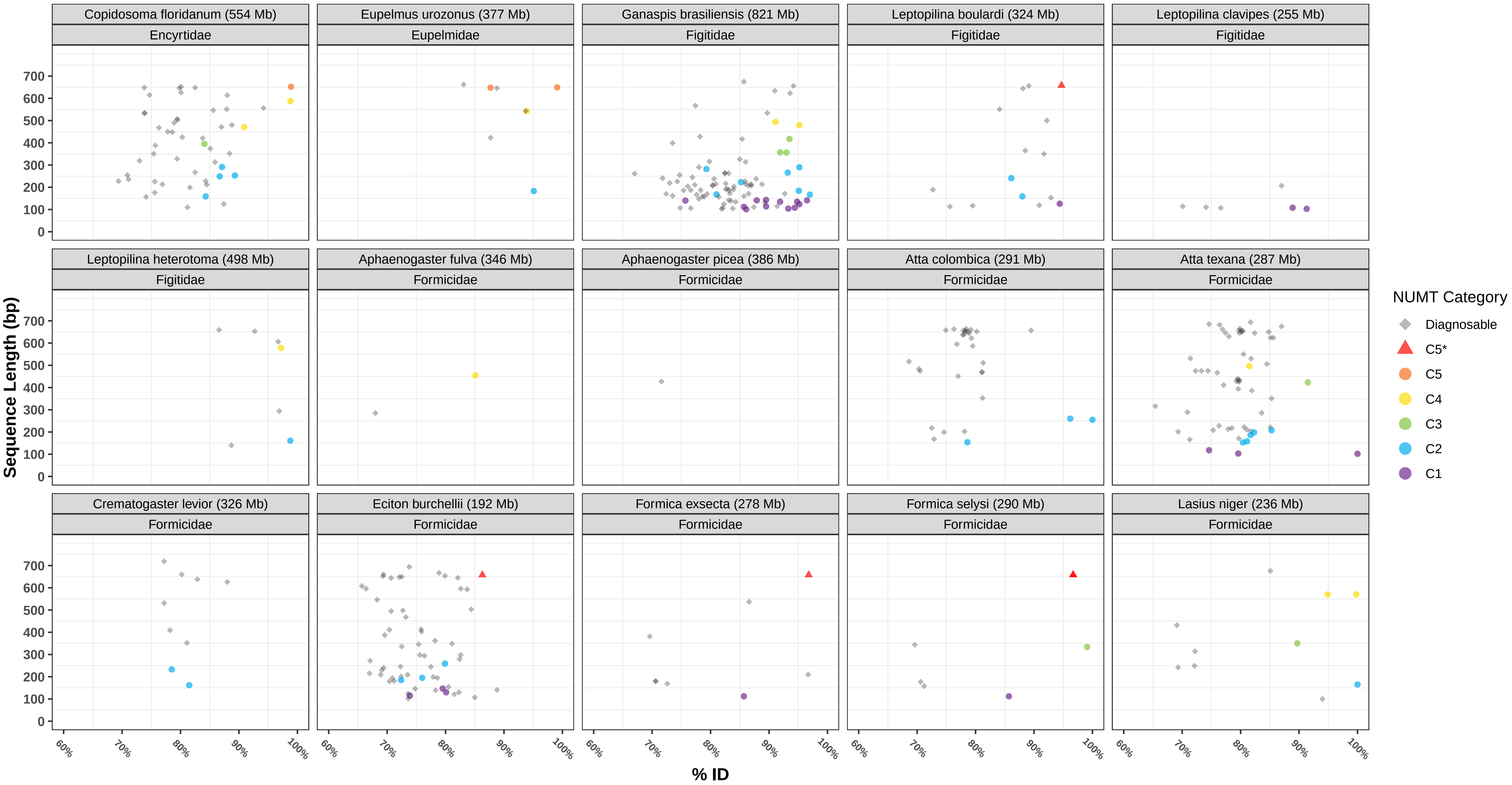

Hymenoptera (pg 6 of 9)

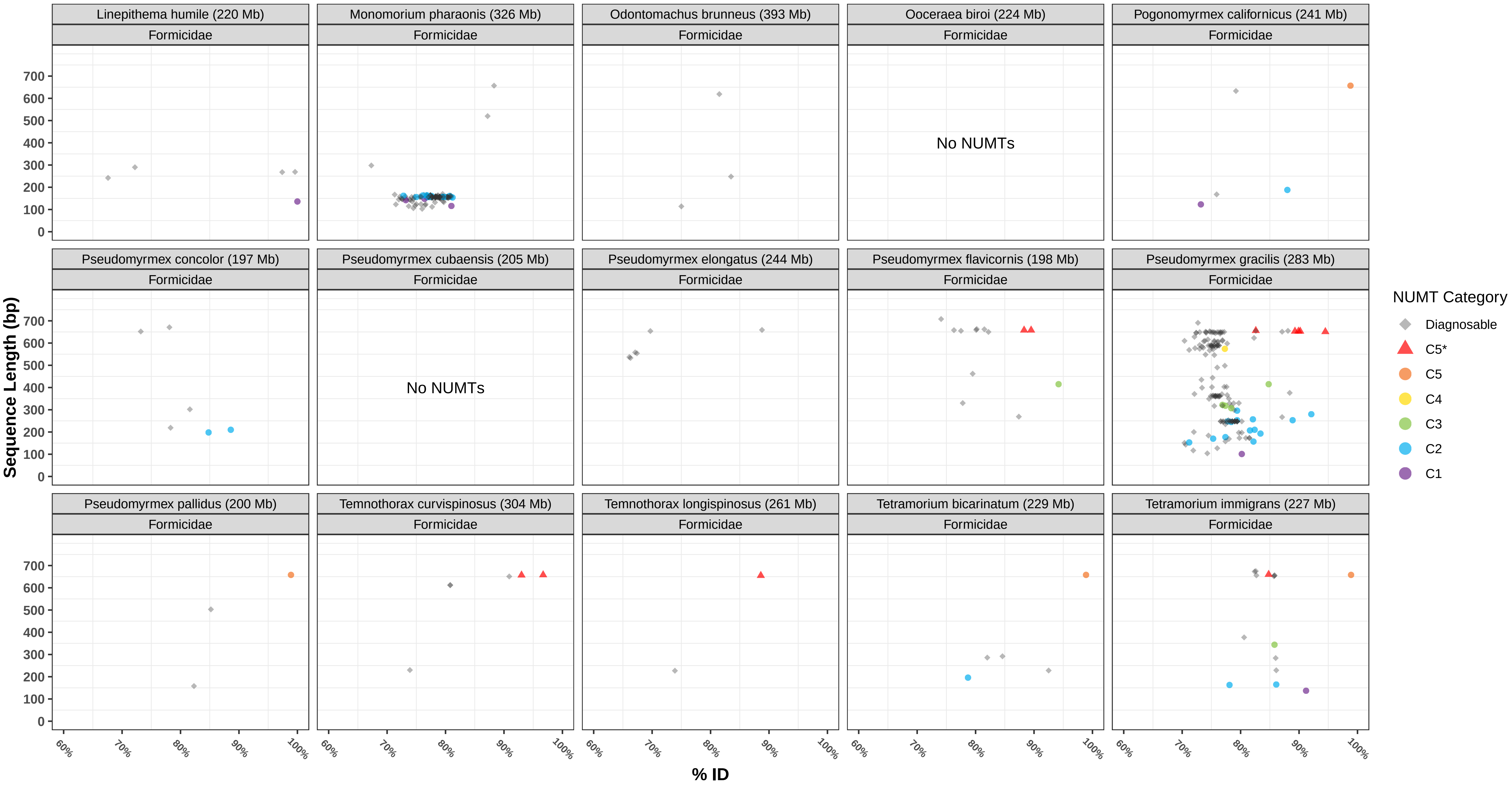

Hymenoptera (pg 7 of 9)

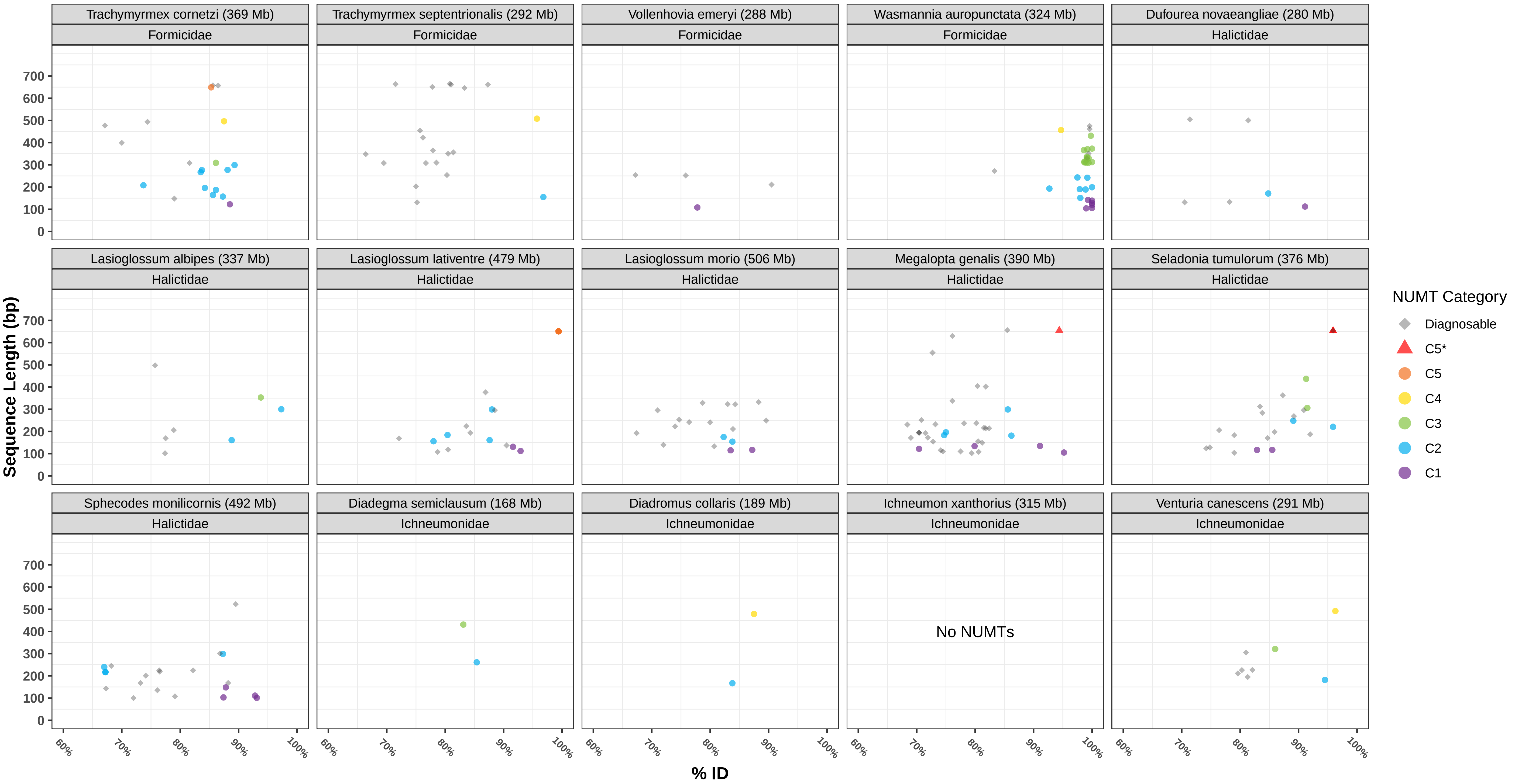

Hymenoptera (pg 8 of 9)

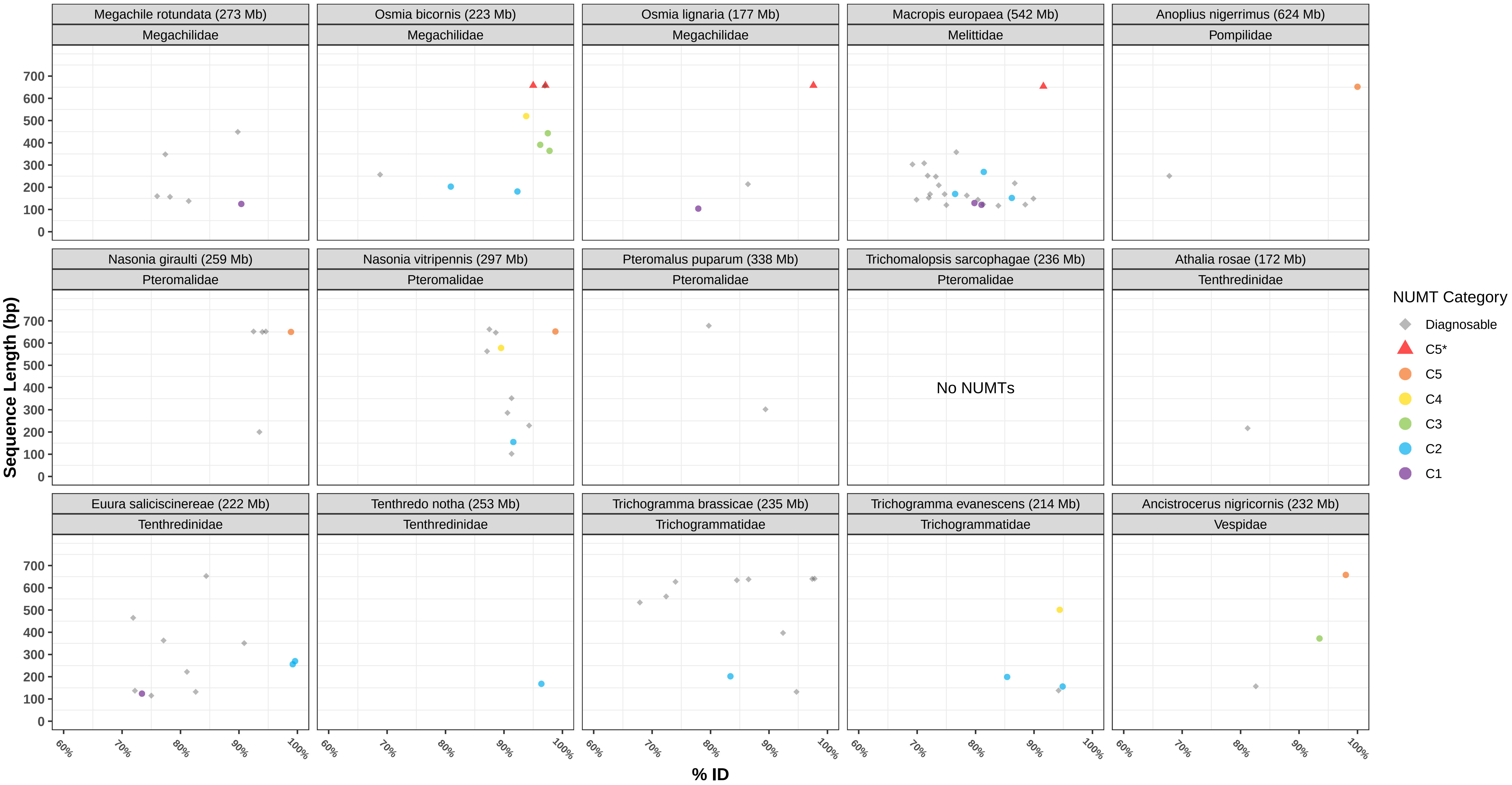

Hymenoptera (pg 9 of 9)

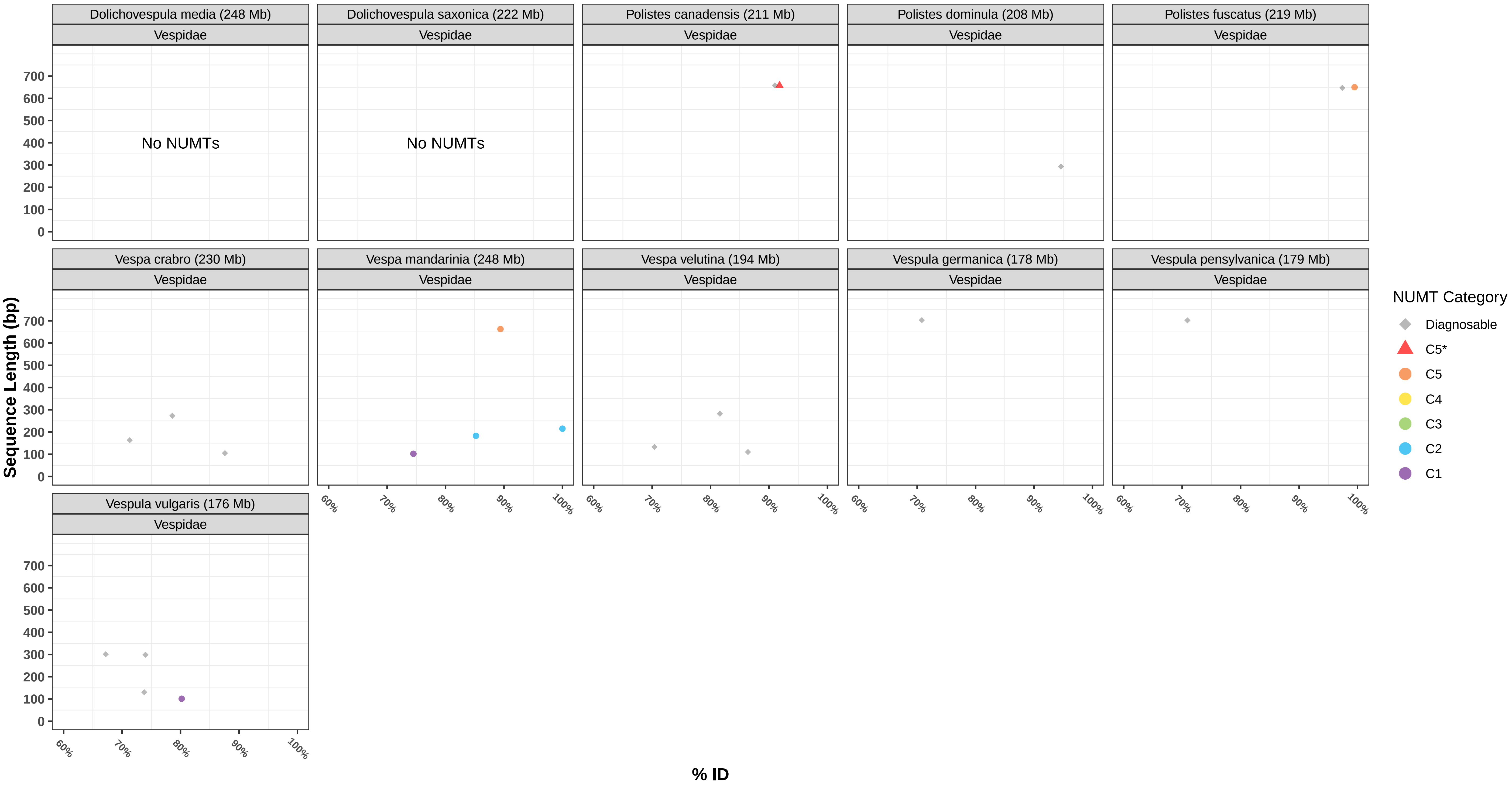

Lepidoptera (pg 1 of 13)

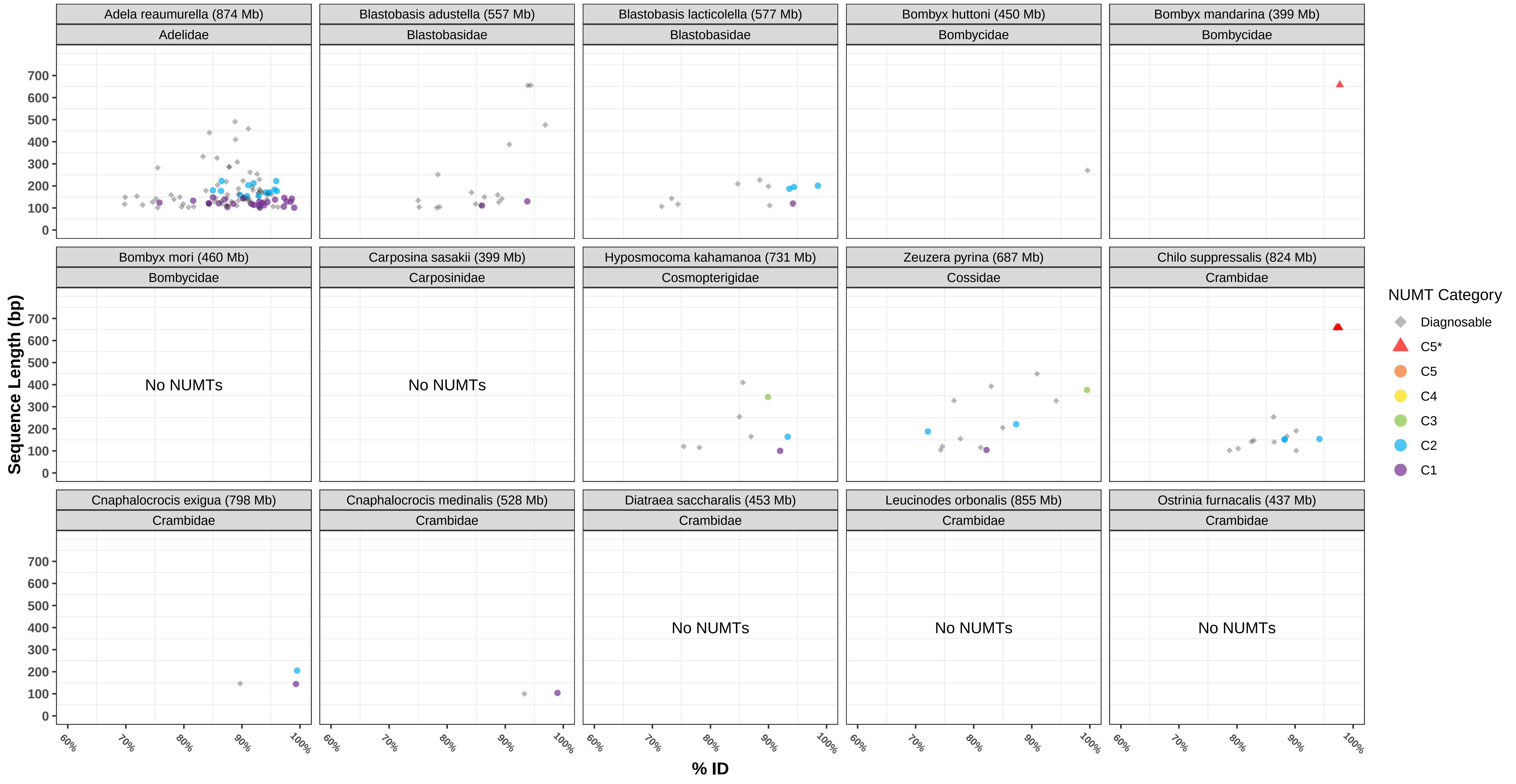

Lepidoptera (pg 2 of 13)

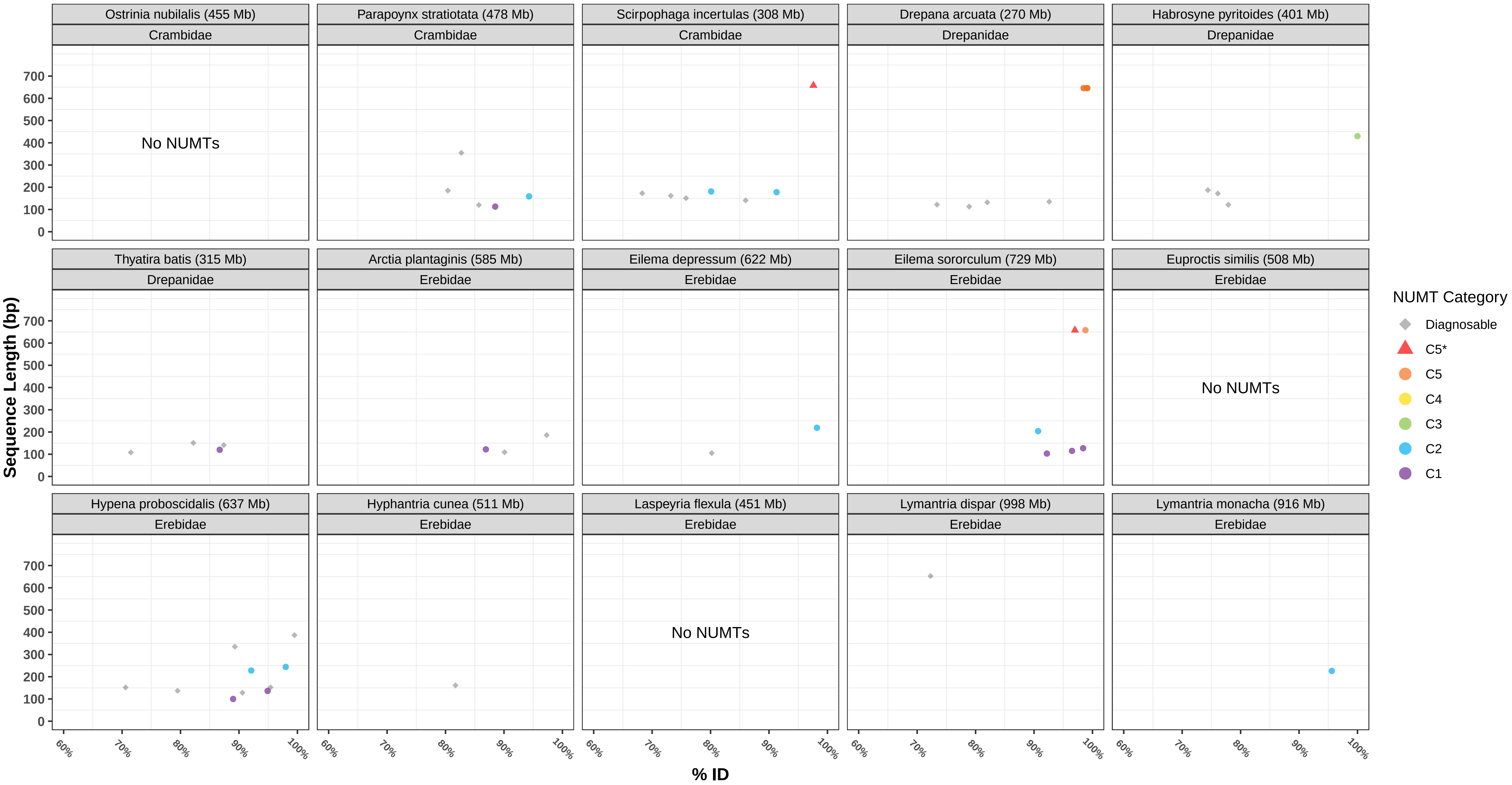

Lepidoptera (pg 3 of 13)

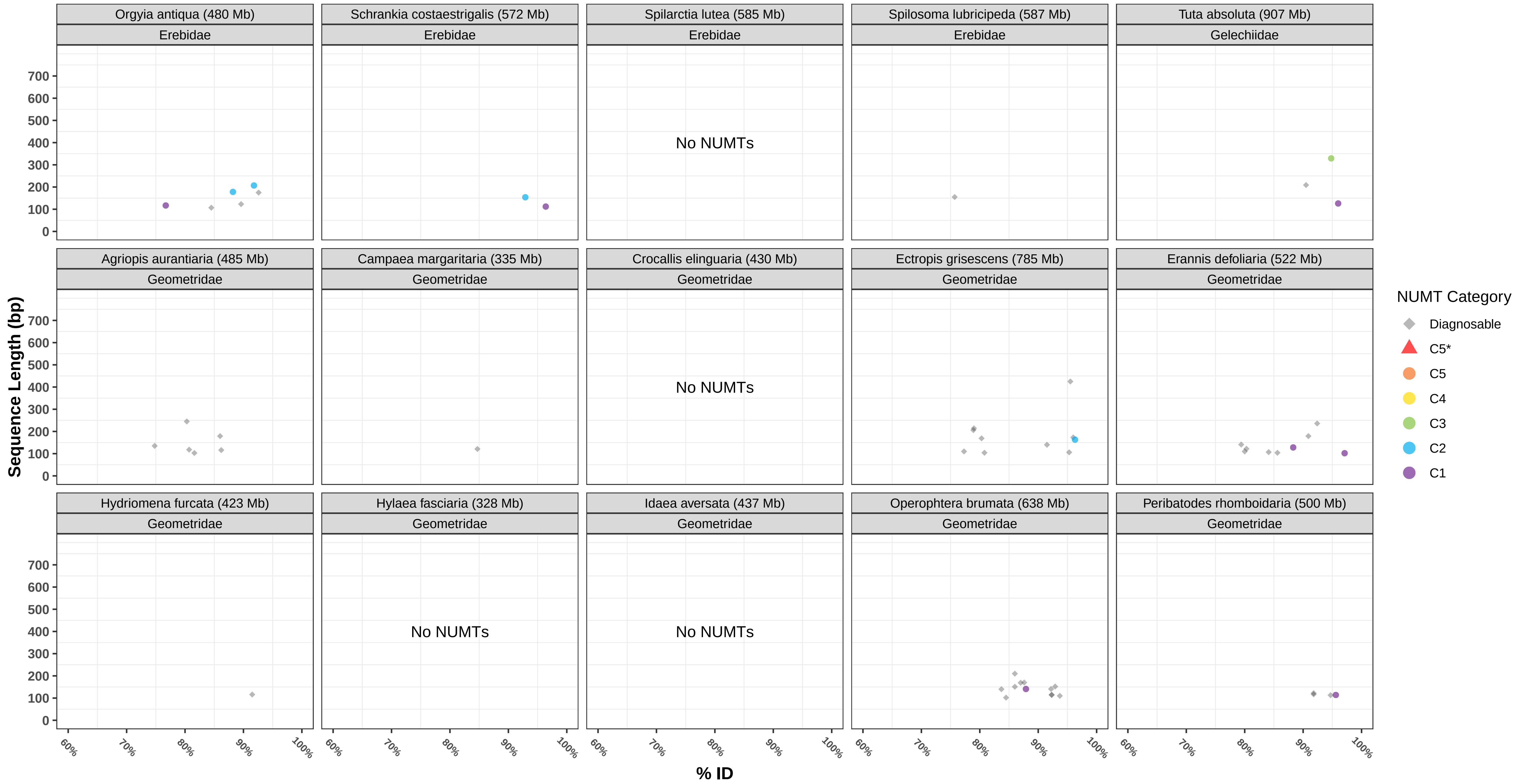

Lepidoptera (pg 4 of 13)

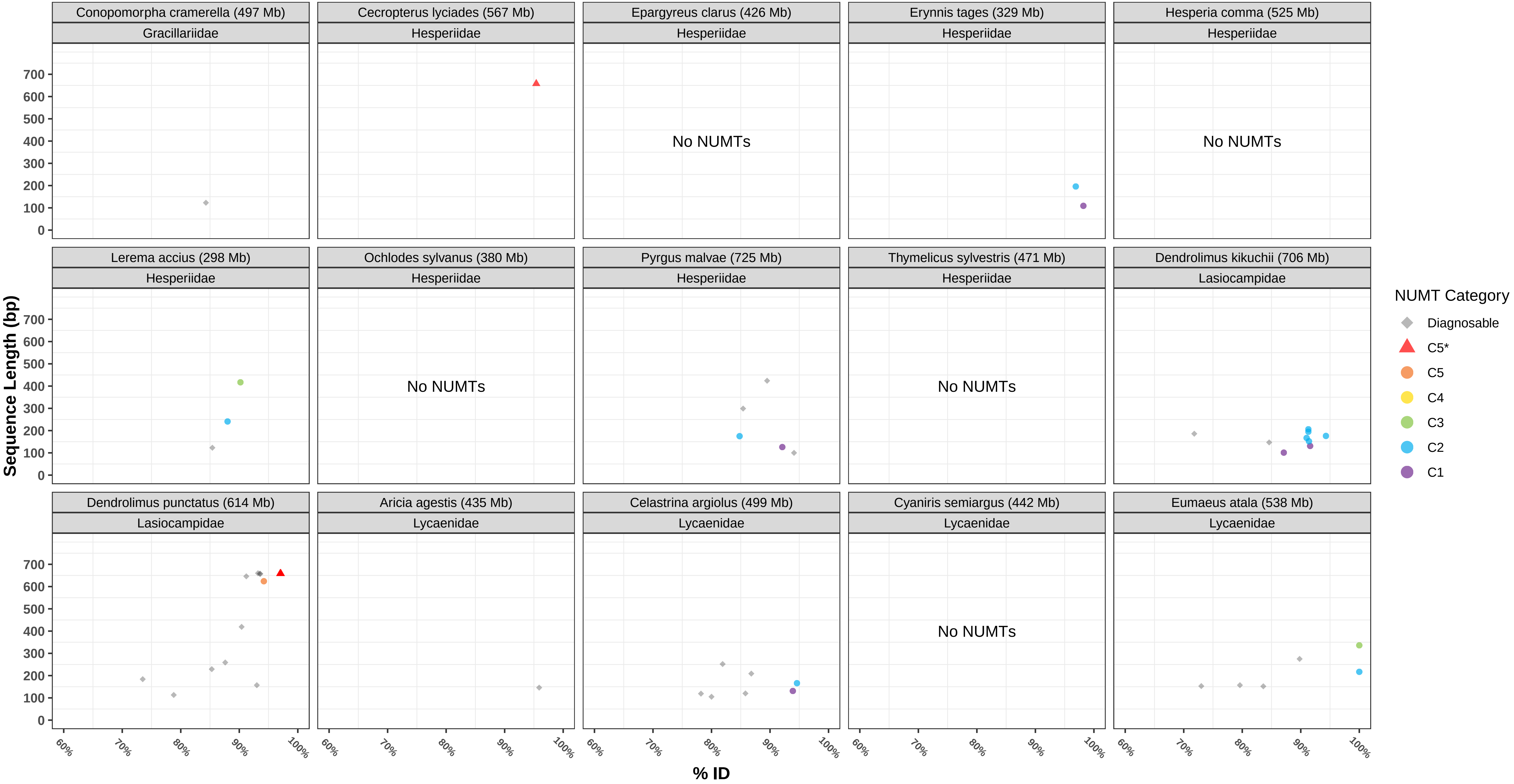

Lepidoptera (pg 5 of 13)

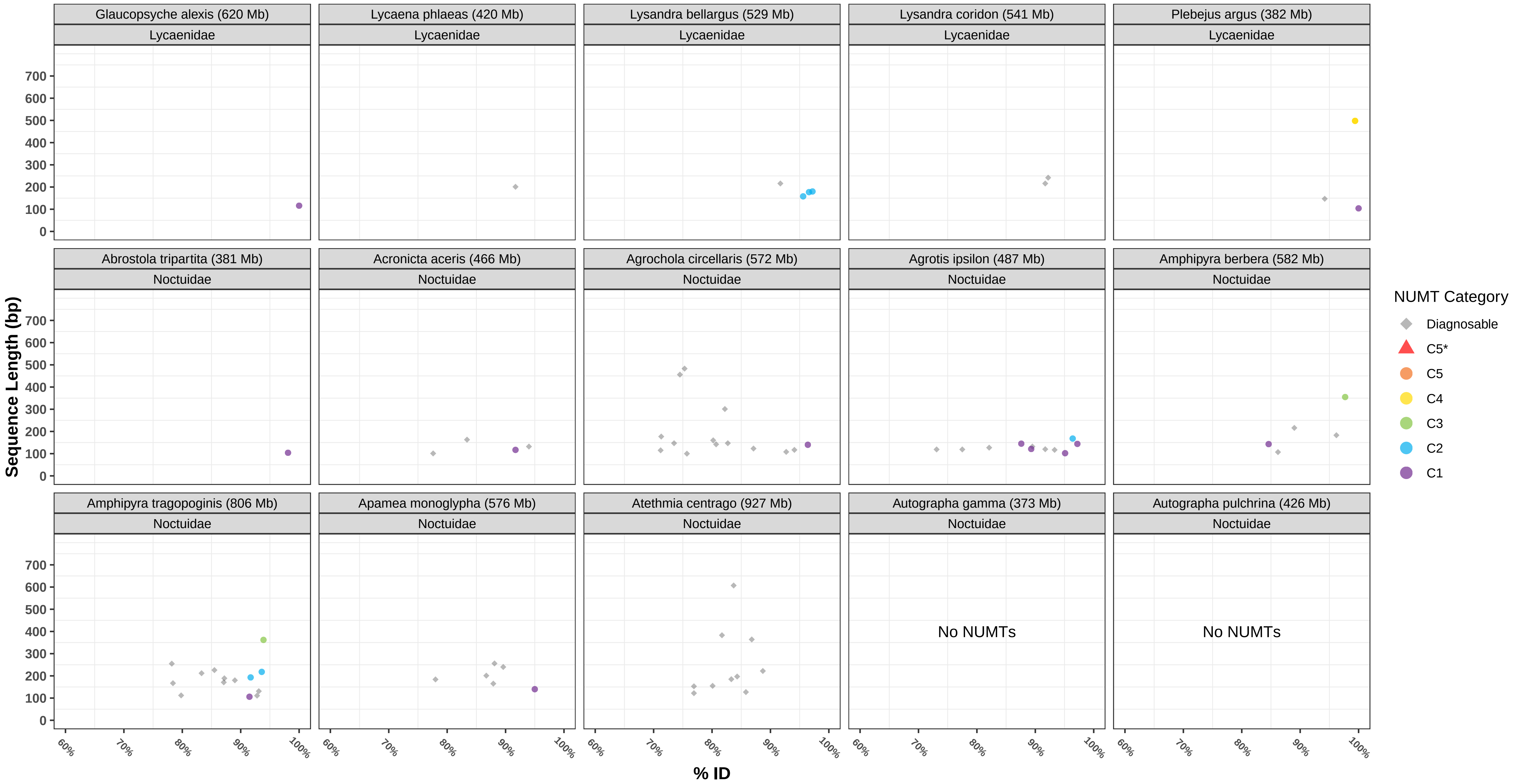

Lepidoptera (pg 6 of 13)

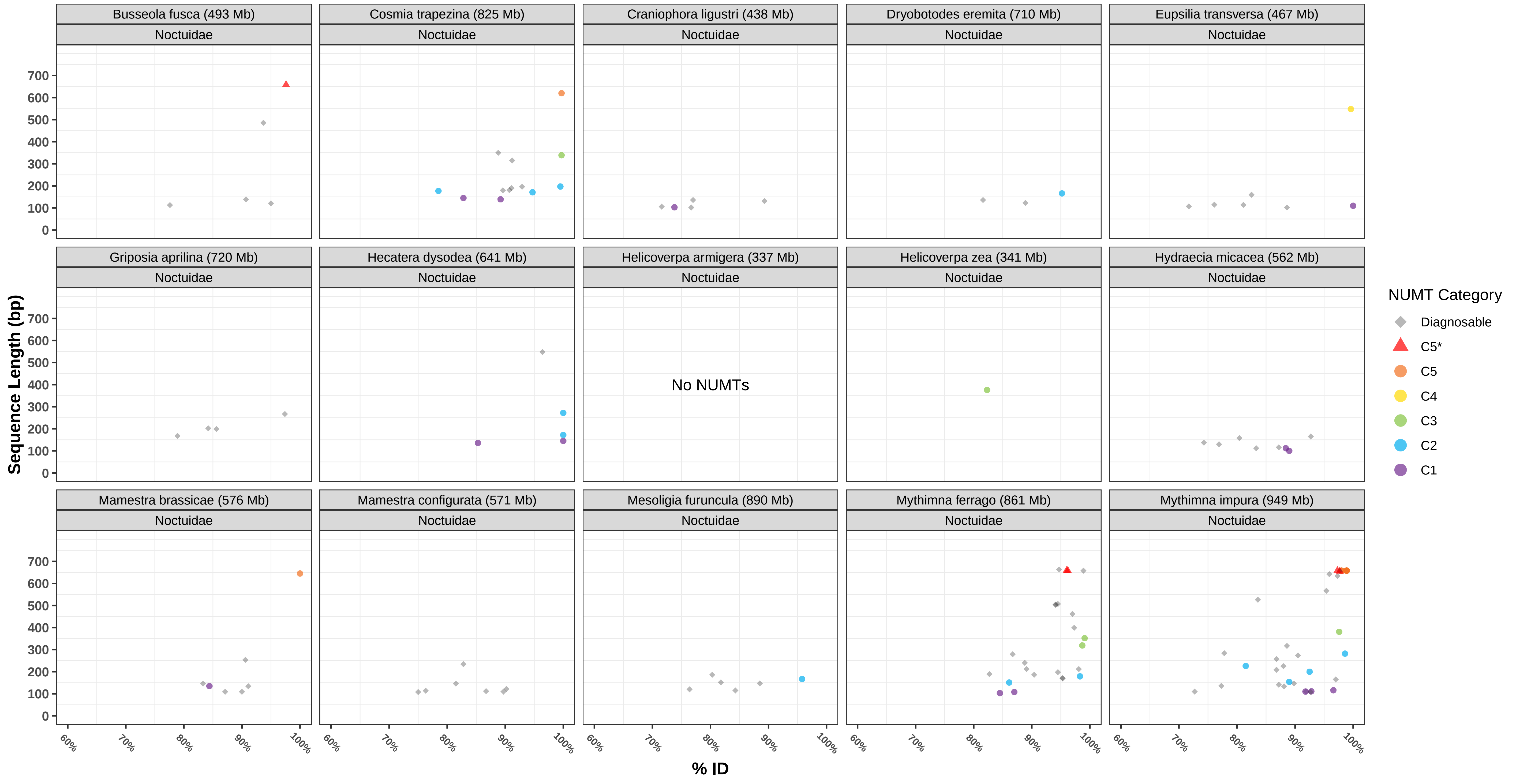

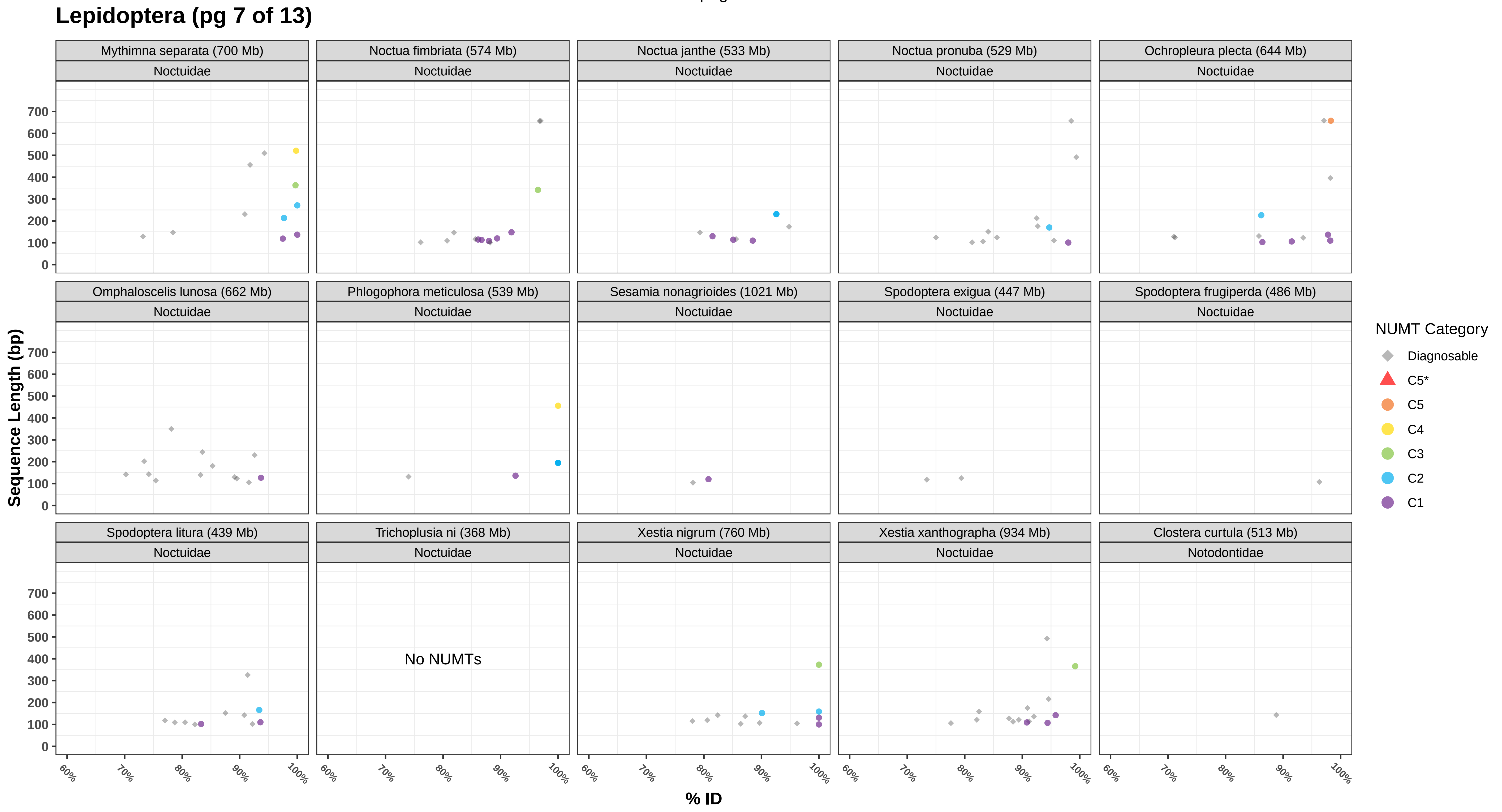
Lepidoptera (pg 7 of 13)
Mythimna separata (700 Mb)
Noctuidae
Noctua fimbriata (574 Mb)
Noctuidae
Noctua janthe (533 Mb)
Noctuidae
Noctua pronuba (529 Mb)
Noctuidae
Ochropleura plecta (644 Mb)
Noctuidae
Omphaloscelis lunosa (662 Mb)
Noctuidae
Phlogophora meticulosa (539 Mb)
Noctuidae
Sesamia nonagrioides (1021 Mb)
Noctuidae
Spodoptera exigua (447 Mb)
Noctuidae
Spodoptera frugiperda (486 Mb)
Noctuidae
Spodoptera litura (439 Mb)
Noctuidae
Trichoplusia ni (368 Mb)
Noctuidae
No NUMTs
Xestia nigrum (760 Mb)
Noctuidae
Xestia xanthographa (934 Mb)
Noctuidae
Clostera curtula (513 Mb)
Notodontidae
Sequence Length (bp)
0
100
200
300
400
500
600
700
% ID
60%
70%
80%
90%
100%
NUMT Category
Diagnosable
C5*
C5
C4
C3
C2
C1

Lepidoptera (pg 8 of 13)

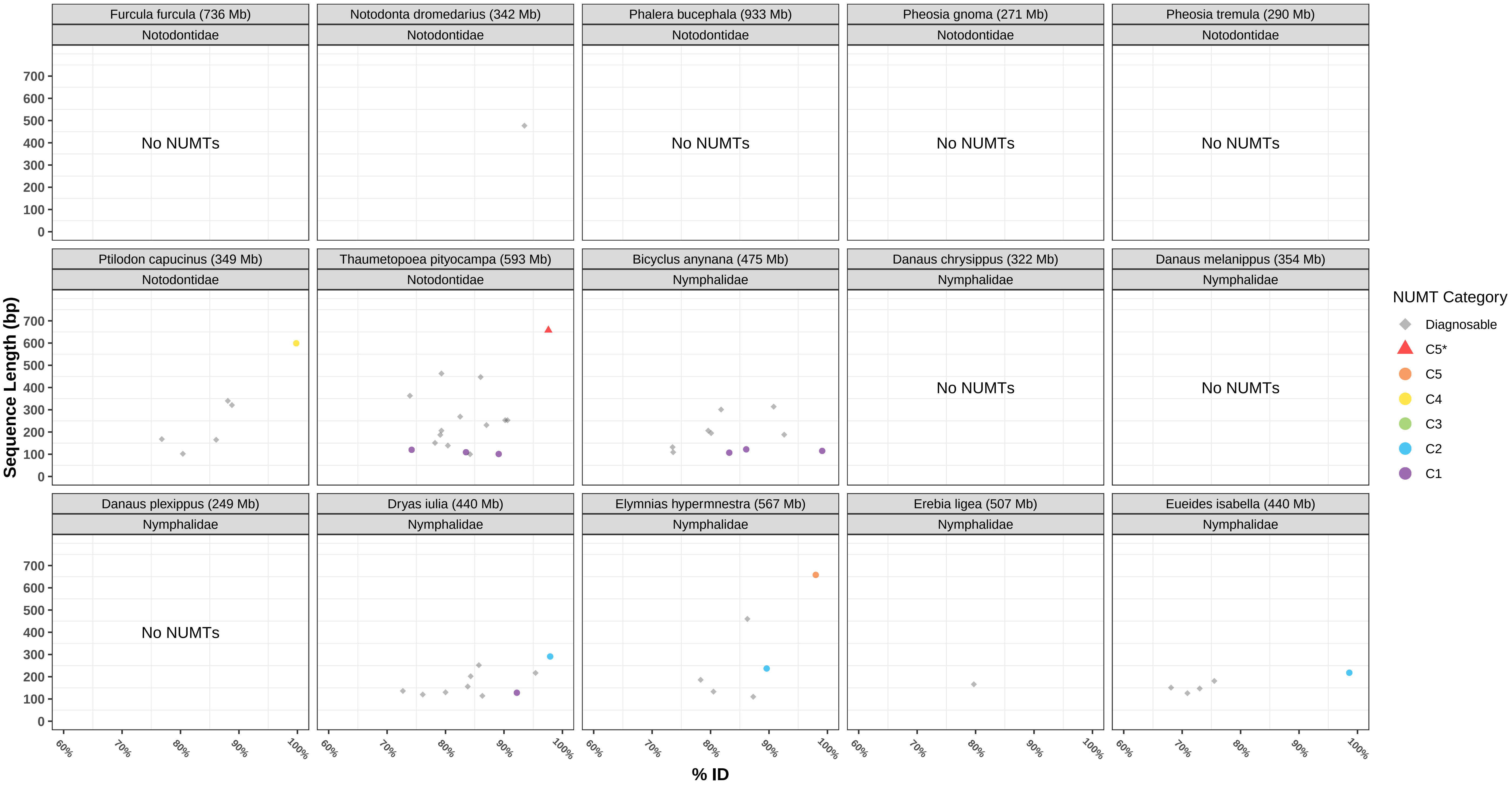

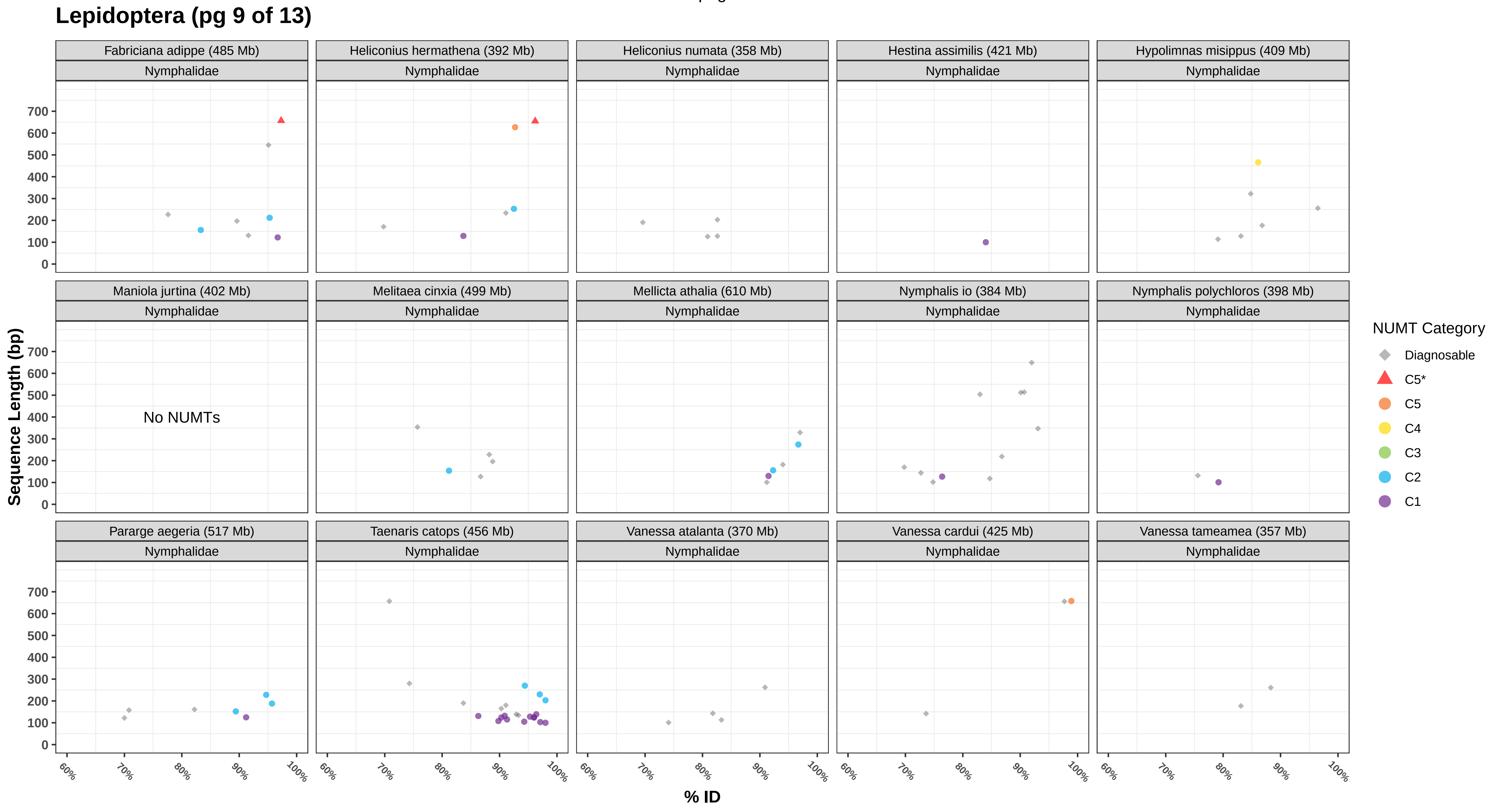
Lepidoptera (pg 9 of 13)
Fabriciana adippe (485 Mb)
Nymphalidae
Heliconius hermathena (392 Mb)
Nymphalidae
Heliconius numata (358 Mb)
Nymphalidae
Hestina assimilis (421 Mb)
Nymphalidae
Hypolimnas misippus (409 Mb)
Nymphalidae
Maniola jurtina (402 Mb)
Nymphalidae
No NUMTs
Melitaea cinxia (499 Mb)
Nymphalidae
Mellicta athalia (610 Mb)
Nymphalidae
Nymphalis io (384 Mb)
Nymphalidae
Nymphalis polychloros (398 Mb)
Nymphalidae
Pararge aegeria (517 Mb)
Nymphalidae
Taenaris catops (456 Mb)
Nymphalidae
Vanessa atalanta (370 Mb)
Nymphalidae
Vanessa cardui (425 Mb)
Nymphalidae
Vanessa tameamea (357 Mb)
Nymphalidae
Sequence Length (bp)
0
100
200
300
400
500
600
700
% ID
60%
70%
80%
90%
100%
NUMT Category
Diagnosable
C5*
C5
C4
C3
C2
C1

Lepidoptera (pg 10 of 13)

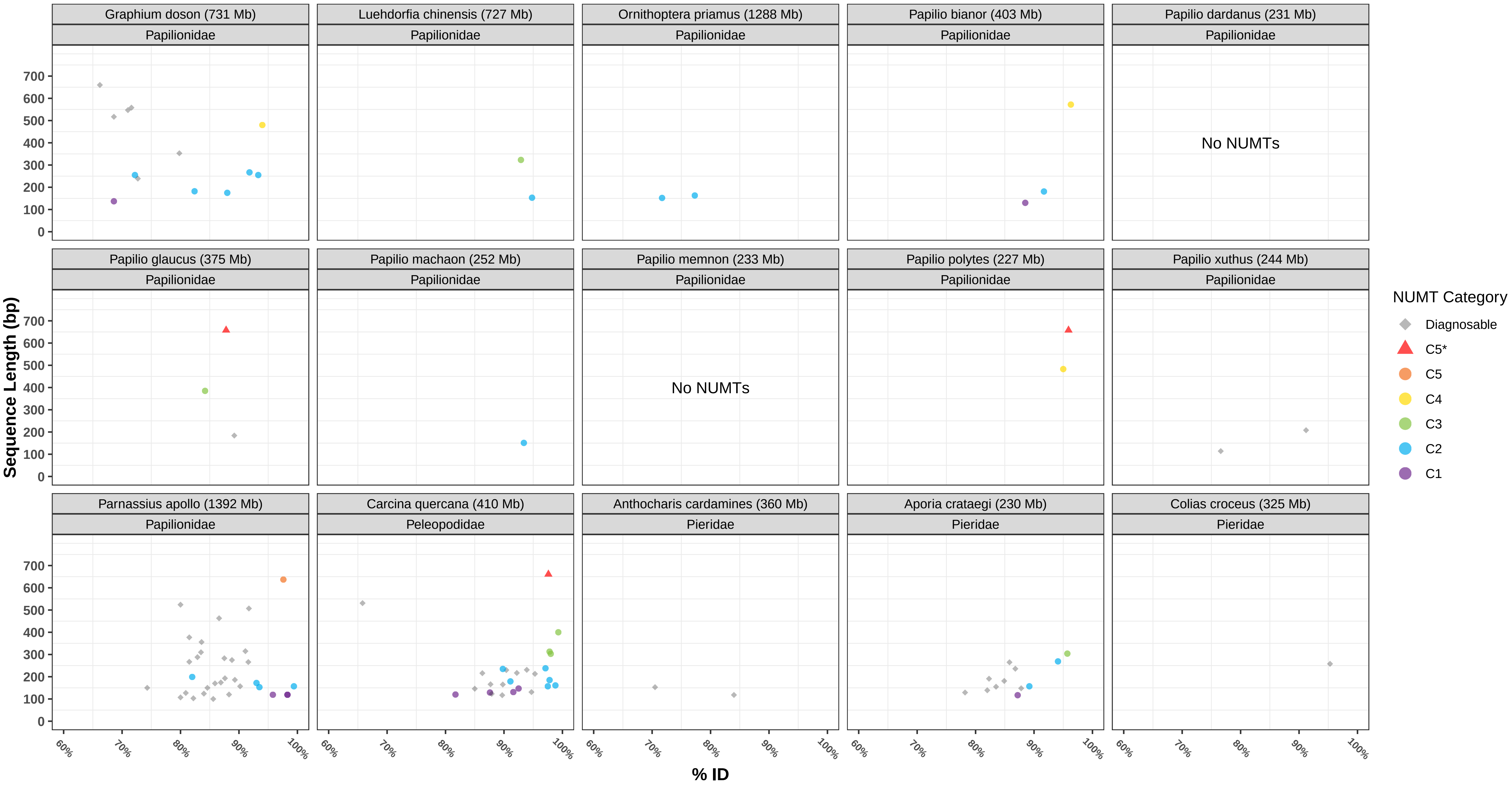

Lepidoptera (pg 11 of 13)

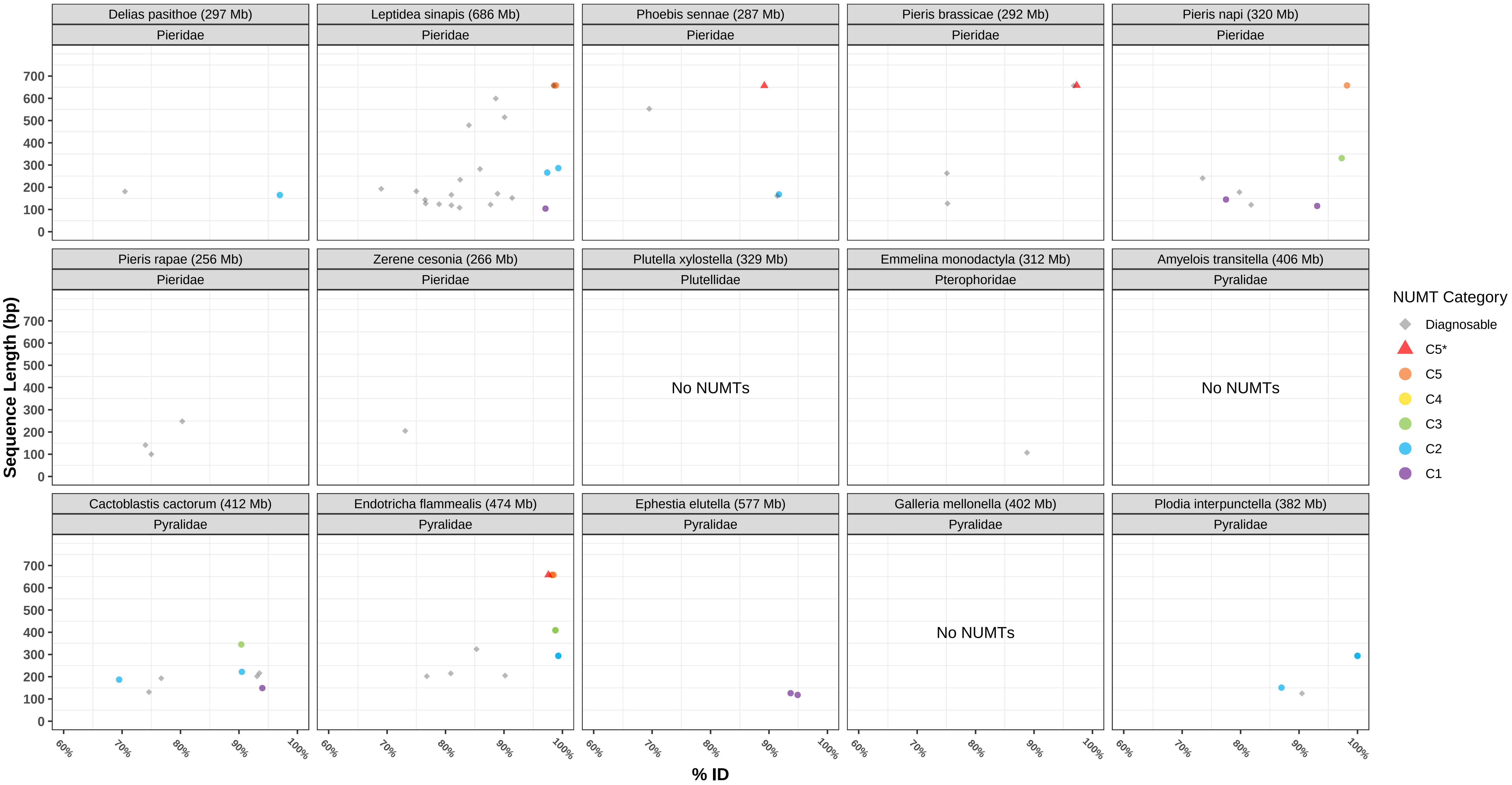

Lepidoptera (pg 12 of 13)

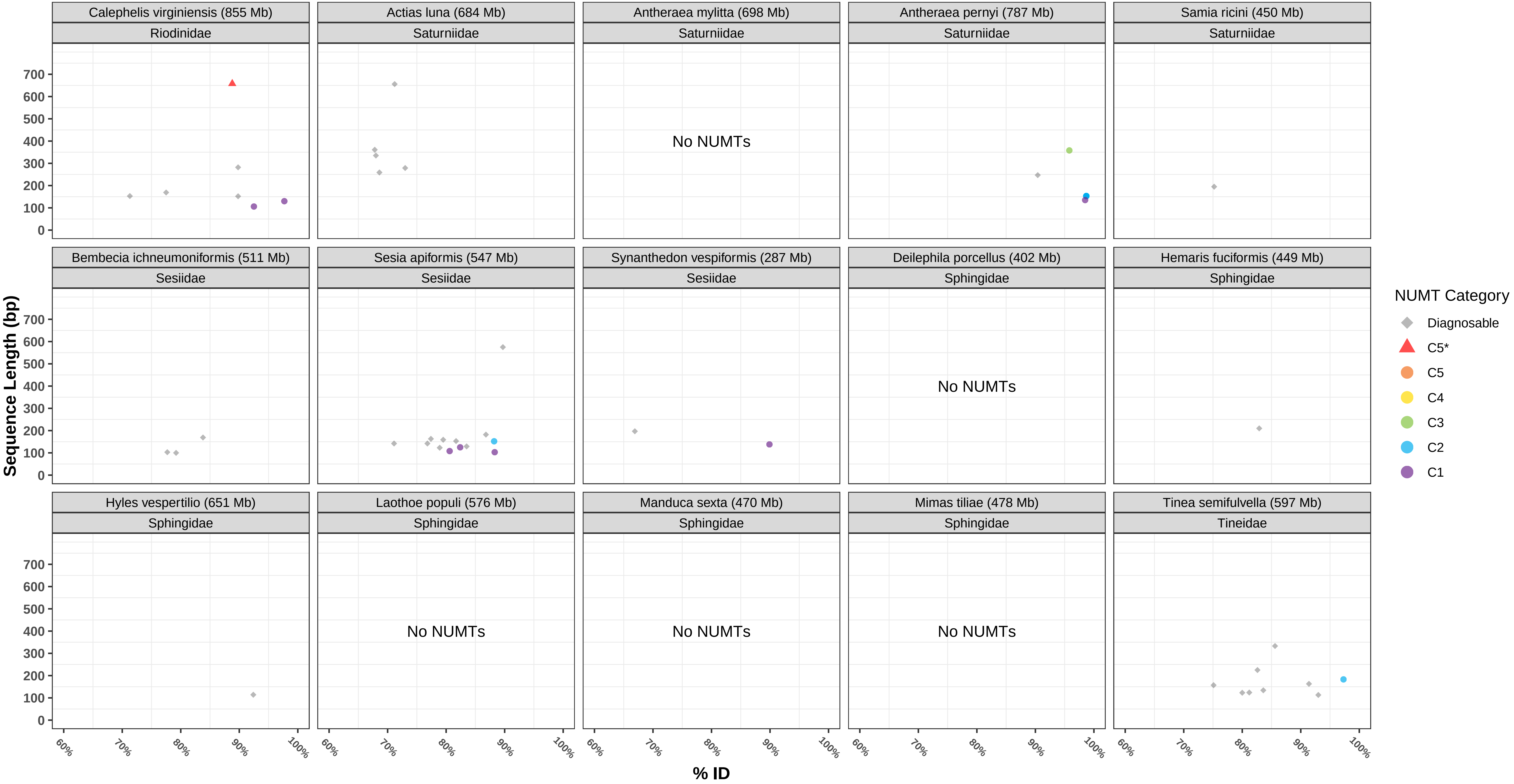

Lepidoptera (pg 13 of 13)

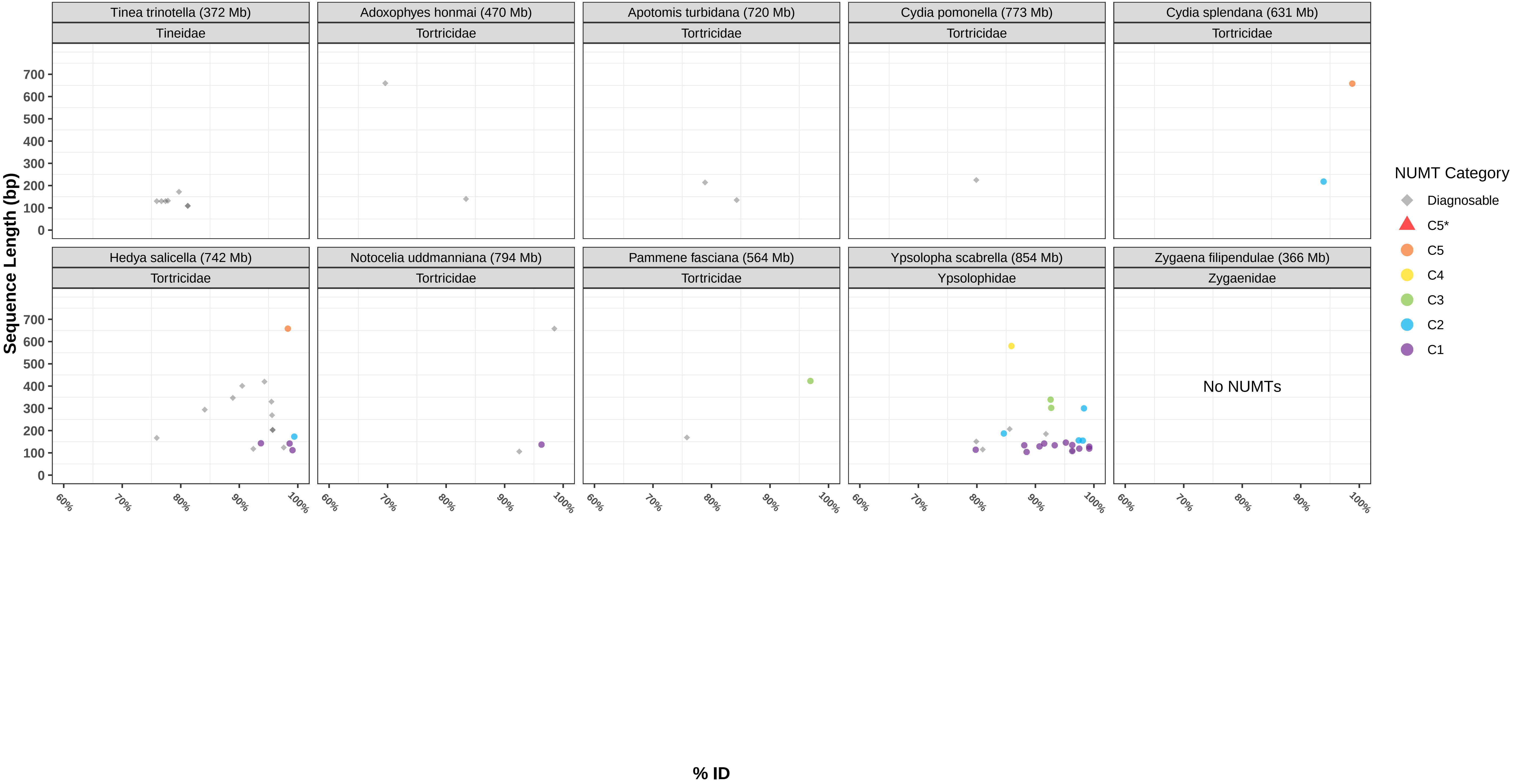

Other Orders (pg 1 of 3)

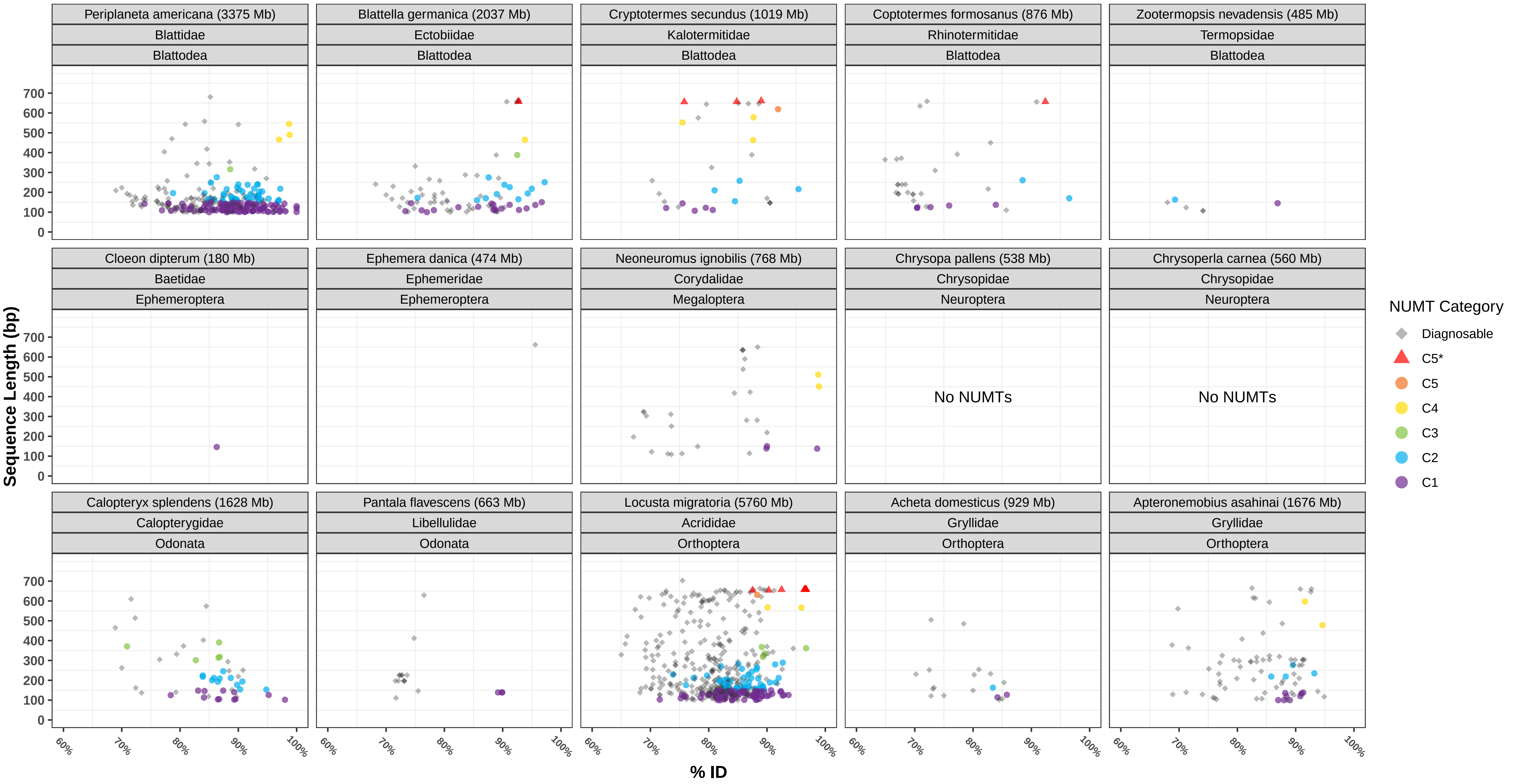

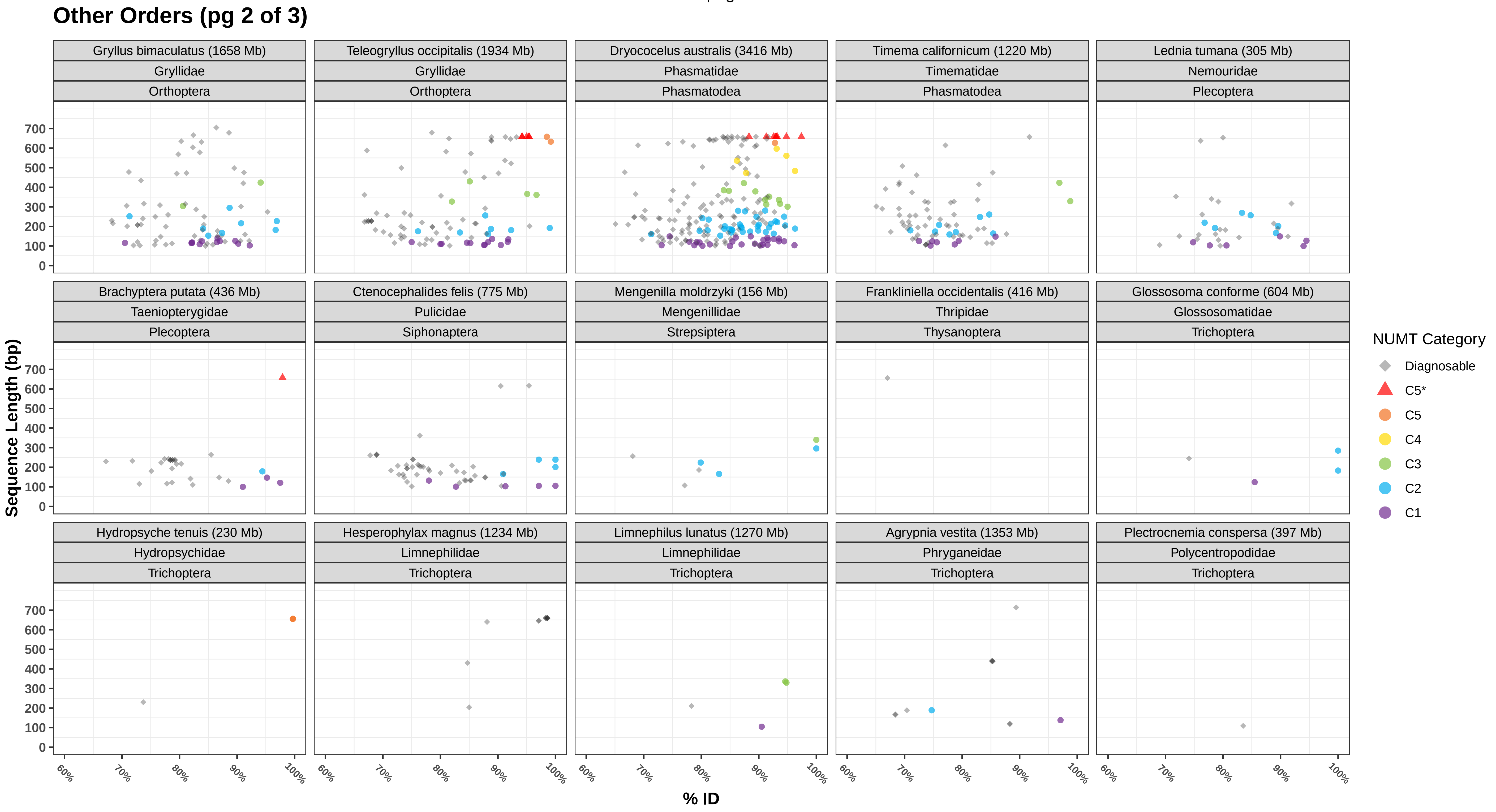
Other Orders (pg 2 of 3)
Gryllus bimaculatus (1658 Mb)
Gryllidae
Orthoptera
Teleogryllus occipitalis (1934 Mb)
Gryllidae
Orthoptera
Dryococelus australis (3416 Mb)
Phasmatidae
Phasmatodea
Timema californicum (1220 Mb)
Timematidae
Phasmatodea
Lednia tumana (305 Mb)
Nemouridae
Plecoptera
Brachyptera putata (436 Mb)
Taeniopterygidae
Plecoptera
Ctenocephalides felis (775 Mb)
Pulicidae
Siphonaptera
Mengenilla moldrzyki (156 Mb)
Mengenillidae
Strepsiptera
Frankliniella occidentalis (416 Mb)
Thripidae
Thysanoptera
Glossosoma conforme (604 Mb)
Glossosomatidae
Trichoptera
Hydropsyche tenuis (230 Mb)
Hydropsychidae
Trichoptera
Hesperophylax magnus (1234 Mb)
Limnephilidae
Trichoptera
Limnephilus lunatus (1270 Mb)
Limnephilidae
Trichoptera
Agrypnia vestita (1353 Mb)
Phryganeidae
Trichoptera
Plectrocnemia conspersa (397 Mb)
Polycentropodidae
Trichoptera
Sequence Length (bp)
0
100
200
300
400
500
600
700
% ID
60%
70%
80%
90%
100%
NUMT Category
Diagnosable
C5*
C5
C4
C3
C2
C1

Other Orders (pg 3 of 3)

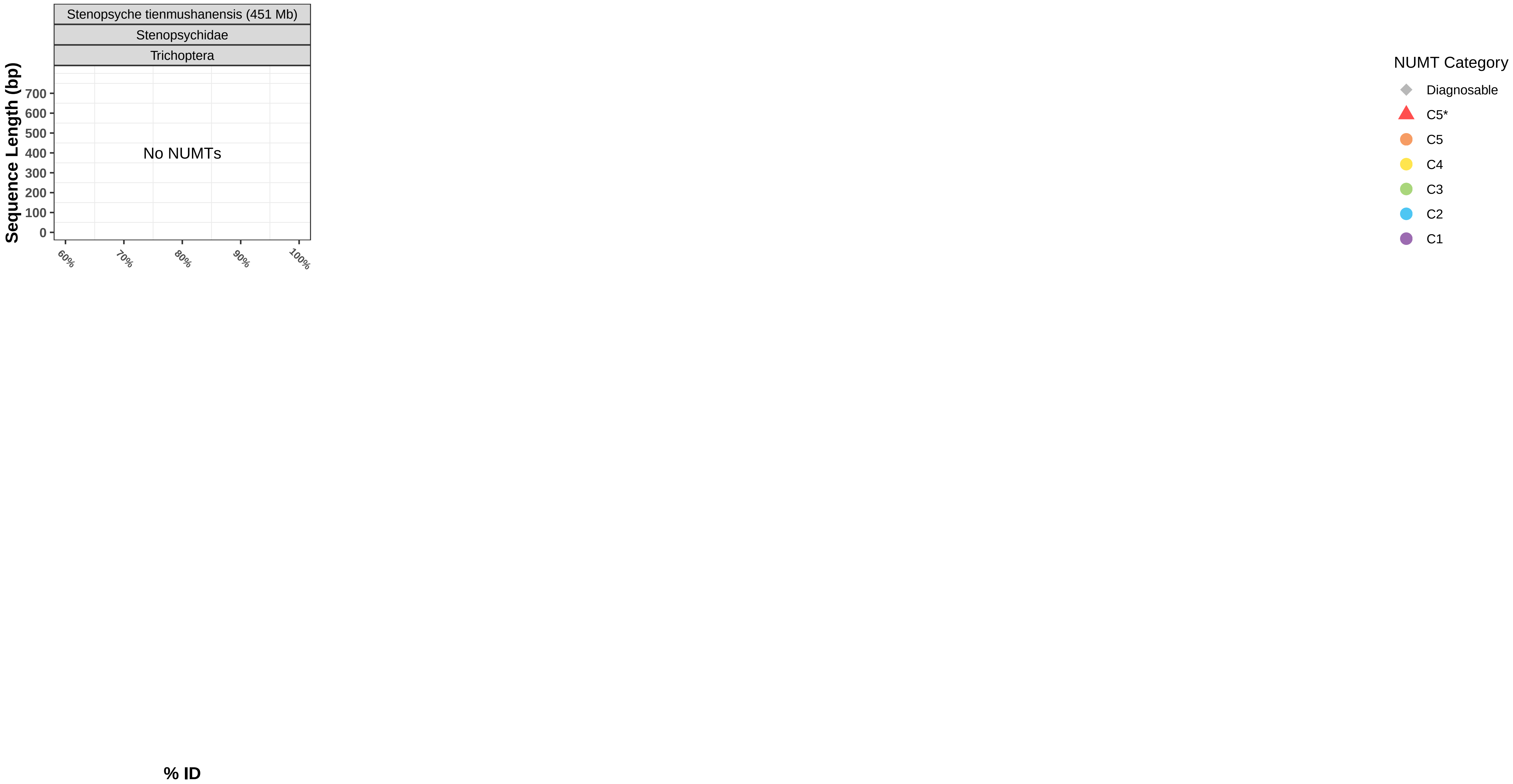

Supplement: S10 Fig — Gray indicates NUMTs with IPSCs (indels or premature stop codons) that enable their recognition and exclusion. Other colours show the varied length categories of NUMTs lacking IPSCs. (PDF) [file pone.0286620.s010.pdf]
