# Supplementary material for: Interrogating 1000 insect genomes for NUMTs: A risk assessment for estimates of species richness
Source: PLoS One. 2023 Jun 8;18(6):e0286620. doi: 10.1371/journal.pone.0286620 (PMC10249859; doi:10.1371/journal.pone.0286620)
Supplement: S3 Table — (DOCX) [file pone.0286620.s016.docx]

| ***High coverage*** |  |  |  | ***Low coverage*** |  |
| --- | --- | --- | --- | --- | --- |
| **Family** | **n** | **Proportion** |  | **Family** | **n** |
| Adelidae | 1 | 0.5 |  | Hesperiidae | 205 |
| Blastobasidae | 2 | 1.1 |  | Lycaenidae | 6 |
| Bombycidae | 3 | 1.6 |  | Nymphalidae | 35 |
| Carposinidae | 1 | 0.5 |  | Papilionidae | 39 |
| Cosmopterigidae | 1 | 0.5 |  | Pieridae | 3 |
| Cossidae | 1 | 0.5 |  | Riodinidae | 39 |
| Crambidae | 9 | 4.7 |  |  |  |
| Drepanidae | 3 | 1.6 |  |  |  |
| Erebidae | 13 | 6.8 |  |  |  |
| Gelechiidae | 1 | 0.5 |  |  |  |
| Geometridae | 10 | 5.3 |  |  |  |
| Gracillariidae | 1 | 0.5 |  |  |  |
| Hesperiidae | 8 | 4.2 |  |  |  |
| Lasiocampidae | 2 | 1.1 |  |  |  |
| Lycaenidae | 9 | 4.7 |  |  |  |
| Noctuidae | 39 | 20.5 |  |  |  |
| Notodontidae | 8 | 4.2 |  |  |  |
| Nymphalidae | 23 | 12.1 |  |  |  |
| Papilionidae | 11 | 5.8 |  |  |  |
| Peleopodidae | 1 | 0.5 |  |  |  |
| Pieridae | 10 | 5.3 |  |  |  |
| Plutellidae | 1 | 0.5 |  |  |  |
| Pterophoridae | 1 | 0.5 |  |  |  |
| Pyralidae | 6 | 3.2 |  |  |  |
| Riodinidae | 1 | 0.5 |  |  |  |
| Saturniidae | 4 | 2.1 |  |  |  |
| Sesiidae | 3 | 1.6 |  |  |  |
| Sphingidae | 6 | 3.2 |  |  |  |
| Tineidae | 2 | 1.1 |  |  |  |
| Tortricidae | 7 | 3.7 |  |  |  |
| Ypsolophidae | 1 | 0.5 |  |  |  |
| Zygaenidae | 1 | 0.5 |  |  |  |
