# Supplementary material for: Interrogating 1000 insect genomes for NUMTs: A risk assessment for estimates of species richness
Source: PLoS One. 2023 Jun 8;18(6):e0286620. doi: 10.1371/journal.pone.0286620 (PMC10249859; doi:10.1371/journal.pone.0286620)
Supplement: S5 Table — ^ Order employing direct development, * order with incomplete metamorphosis. Those lacking a symbol develop via complete metamorphosis. n = number of families examined in each order. (DOCX) [file pone.0286620.s018.docx]

| Order | Mean Genome Size | n | <200 | 201 - 400 | 401 - 800 | 801 - 1600 | 1601 - 3200 | 3201 - 6400 | >6401 |
| --- | --- | --- | --- | --- | --- | --- | --- | --- | --- |
| Archaeognatha^ | 2,887 | 1 |  |  |  |  | 1 |  |  |
| Zoraptera^ | 1,848 | 1 |  |  |  |  | 1 |  |  |
| Zygentoma^ | 3,022 | 1 |  |  |  |  | 1 |  |  |
| Blattodea* | 1,779 | 12 |  |  | 1 | 7 | 3 | 1 | - |
| Dermaptera* | 580 | 2 |  |  | 2 |  |  |  |  |
| Embioptera* | 2,601 | 1 |  |  |  |  | 1 |  |  |
| Ephemeroptera* | 443 | 1 | 1 | - | 1 |  |  |  |  |
| Hemiptera* | 870 | 23 | - | 3 | 9 | 8 | 1 | 2 |  |
| Mantodea* | 3,476 | 2 |  |  |  |  |  | 2 | - |
| Notoptera* | 1,600 | 1 |  |  |  |  | 1 |  |  |
| Odonata* | 1,012 | 10 |  |  | 3 | 5 | 2 |  |  |
| Orthoptera* | 7,737 | 8 |  |  |  |  | 2 | 4 | 2 |
| Phasmatodea | 2,158 | 4 |  |  |  | 1 | 2 | 1 |  |
| Plecoptera* | 370 | 2 |  | 1 | 1 |  |  |  |  |
| Coleoptera | 712 | 34 | 2 | 7 | 11 | 11 | 3 |  |  |
| Diptera | 344 | 32 | 6 | 7 | 16 | 3 |  |  |  |
| Hymenoptera | 383 | 38 | 5 | 18 | 14 | 1 |  |  |  |
| Lepidoptera | 543 | 38 |  | 7 | 27 | 4 |  |  |  |
| Mecoptera | 2,015 | 1 |  |  |  |  | 1 |  |  |
| Megaloptera | 768 | 1 |  |  | 1 |  |  |  |  |
| Neuroptera | 549 | 1 |  |  | 1 |  |  |  |  |
| Psocodea | 81 | 2 | 2 |  |  |  |  |  |  |
| Siphonaptera | 775 | 1 |  |  | 1 |  |  |  |  |
| Strepsiptera | 130 | 3 | 3 |  |  |  |  |  |  |
| Trichoptera | 791 | 6 |  | 2 | 2 | 2 |  |  |  |
| Thysanoptera | 369 | 2 |  | 1 | 1 |  |  |  |  |
