# Supplementary material for: Interrogating 1000 insect genomes for NUMTs: A risk assessment for estimates of species richness
Source: PLoS One. 2023 Jun 8;18(6):e0286620. doi: 10.1371/journal.pone.0286620 (PMC10249859; doi:10.1371/journal.pone.0286620)
Supplement: S6 Table — Analysis only considered orders represented by five or more families. * represented by a single species. (DOCX) [file pone.0286620.s019.docx]

| *Order* | *# Families* | *Mean genome size (Mb)* | | *Mean genome size (Mb)* | | *Ratio* |
| --- | --- | --- | --- | --- | --- | --- |
| Coleoptera | 33 | 100 | Silvanidae* | 2,230 | Phengodidae* | 22.3 |
| Hemiptera | 22 | 236 | Pseudococcidae | 4,209 | Cicadidae | 17.8 |
| Diptera | 32 | 70 | Agromyzidae* | 1,005 | Stratiomyiidae* | 14.3 |
| Hymenoptera | 37 | 128 | Scelionidae* | 1,188 | Cynipidae | 9.3 |
| Blattodea | 12 | 485 | Archotermitidae* | 3,562 | Blaberidae | 7.4 |
| Trichoptera | 5 | 230 | Hydropsychidae* | 1,353 | Phyrganeidae* | 5.9 |
| Orthoptera | 8 | 1,674 | Gryllidae | 9,587 | Acrididae | 5.7 |
| Odonata | 10 | 627 | Lestidae | 1,872 | Chlorocyphidae* | 3.0 |
| Lepidoptera | 37 | 312 | Pterophoridae* | 907 | Gelechiidae* | 2.9 |
