# Supplementary material for: Interrogating 1000 insect genomes for NUMTs: A risk assessment for estimates of species richness
Source: PLoS One. 2023 Jun 8;18(6):e0286620. doi: 10.1371/journal.pone.0286620 (PMC10249859; doi:10.1371/journal.pone.0286620)
Supplement: S7 Table — (DOCX) [file pone.0286620.s020.docx]

| **Order** | **Family** | **Species** | **Source** |
| --- | --- | --- | --- |
| Blattodea | Archotermopsidae | *Zootermopsis nevadensis* | Public: Annotated |
| Blattodea | Blattidae | *Periplaneta americana* | Public: Annotated |
| Blattodea | Ectobiidae | *Blattella germanica* | Public: Annotated |
| Blattodea | Rhinotermitidae | *Coptotermes formosanus* | Public: Annotated |
| Coleoptera | Buprestidae | *Agrilus planipennis* | Public: Annotated |
| Coleoptera | Cantharidae | *Cantharis rustica* | Public: Non-annotated |
| Coleoptera | Cantharidae | *Rhagonycha fulva* | Public: Non-annotated |
| Coleoptera | Carabidae | *Pterostichus madidus* | Public: Non-annotated |
| Coleoptera | Cerambycidae | *Anoplophora glabripennis* | Public: Annotated |
| Coleoptera | Chrysomelidae | *Leptinotarsa lineolata* | Mined from scaffolds |
| Coleoptera | Chrysomelidae | *Leptinotarsa rubiginosa* | Mined from scaffolds |
| Coleoptera | Chrysomelidae | *Ophraella communa* | Public: Annotated |
| Coleoptera | Coccinellidae | *Harmonia axyridis* | Public: Non-annotated |
| Coleoptera | Curculionidae | *Elaeidobius kamerunicus* | Public: Annotated |
| Coleoptera | Curculionidae | *Sitophilus oryzae* | Public: Annotated |
| Coleoptera | Elateridae | *Limonius californicus* | Public: Annotated |
| Coleoptera | Lampyridae | *Abscondita terminalis* | Public: Annotated |
| Coleoptera | Lampyridae | *Lamprigera yunnana* | Public: Annotated |
| Coleoptera | Meloidae | *Hycleus cichorii* | Public: Annotated |
| Coleoptera | Meloidae | *Hycleus phaleratus* | Public: Annotated |
| Coleoptera | Nitidulidae | *Aethina tumida* | Public: Annotated |
| Coleoptera | Pyrochroidae | *Pyrochroa serraticornis* | Public: Non-annotated |
| Coleoptera | Scarabaeidae | *Popillia japonica* | Public: Annotated |
| Coleoptera | Scarabaeidae | *Protaetia brevitarsis* | Public: Annotated |
| Coleoptera | Staphylinidae | *Ocypus olens* | Public: Non-annotated |
| Coleoptera | Tenebrionidae | *Asbolus verrucosus* | Public: Annotated |
| Coleoptera | Tenebrionidae | *Tenebrio molitor* | Public: Annotated |
| Coleoptera | Tenebrionidae | *Tribolium castaneum* | Public: Annotated |
| Coleoptera | Tenebrionidae | *Tribolium confusum* | Public: Annotated |
| Diptera | Agromyzidae | *Liriomyza trifolii* | Public: Annotated |
| Diptera | Asilidae | *Dasypogon diadema* | Public: Annotated |
| Diptera | Calliphoridae | *Calliphora vicina* | Public: Annotated |
| Diptera | Calliphoridae | *Cochliomyia hominivorax* | Public: Annotated |
| Diptera | Calliphoridae | *Lucilia cuprina* | Public: Annotated |
| Diptera | Calliphoridae | *Lucilia sericata* | Public: Annotated |
| Diptera | Calliphoridae | *Phormia regina* | Public: Annotated |
| Diptera | Cecidomyiidae | *Mayetiola destructor* | Public: Annotated |
| Diptera | Ceratopogonidae | *Culicoides sonorensis* | Mined from scaffolds |
| Diptera | Chironomidae | *Clunio marinus* | Mined from scaffolds |
| Diptera | Chironomidae | *Polypedilum vanderplanki* | Public: Annotated |
| Diptera | Coelopidae | *Coelopa frigida* | Mined from scaffolds |
| Diptera | Culicidae | *Aedes aegypti* | Public: Annotated |
| Diptera | Culicidae | *Aedes albopictus* | Public: Annotated |
| Diptera | Culicidae | *Anopheles arabiensis* | Public: Annotated |
| Diptera | Culicidae | *Anopheles atroparvus* | Public: Annotated |
| Diptera | Culicidae | *Anopheles christyi* | Public: Annotated |
| Diptera | Culicidae | *Anopheles coluzzii* | Public: Non-annotated |
| Diptera | Culicidae | *Anopheles cracens* | Public: Annotated |
| Diptera | Culicidae | *Anopheles culicifacies* | Public: Annotated |
| Diptera | Culicidae | *Anopheles darlingi* | Public: Annotated |
| Diptera | Culicidae | *Anopheles dirus* | Public: Annotated |
| Diptera | Culicidae | *Anopheles epiroticus* | Public: Annotated |
| Diptera | Culicidae | *Anopheles funestus* | Public: Annotated |
| Diptera | Culicidae | *Anopheles gambiae* | Public: Non-annotated |
| Diptera | Culicidae | *Anopheles longipalpis* | Public: Non-annotated |
| Diptera | Culicidae | *Anopheles maculatus* | Public: Annotated |
| Diptera | Culicidae | *Anopheles melas* | Public: Annotated |
| Diptera | Culicidae | *Anopheles merus* | Public: Annotated |
| Diptera | Culicidae | *Anopheles minimus* | Public: Annotated |
| Diptera | Culicidae | *Anopheles nili* | Mined from scaffolds |
| Diptera | Culicidae | *Anopheles parensis* | Public: Non-annotated |
| Diptera | Culicidae | *Anopheles punctulatus* | Public: Annotated |
| Diptera | Culicidae | *Anopheles rivulorum* | Mined from scaffolds |
| Diptera | Culicidae | *Anopheles sinensis* | Public: Annotated |
| Diptera | Culicidae | *Anopheles stephensi* | Public: Annotated |
| Diptera | Culicidae | *Anopheles vaneedeni* | Public: Non-annotated |
| Diptera | Culicidae | *Culex pipiens* | Public: Annotated |
| Diptera | Culicidae | *Culex quinquefasciatus* | Public: Annotated |
| Diptera | Drosophilidae | *Drosophila albomicans* | Public: Annotated |
| Diptera | Drosophilidae | *Drosophila busckii* | Public: Annotated |
| Diptera | Drosophilidae | *Drosophila mauritiana* | Public: Annotated |
| Diptera | Drosophilidae | *Drosophila melanogaster* | Public: Annotated |
| Diptera | Drosophilidae | *Drosophila pseudoobscura* | Public: Annotated |
| Diptera | Drosophilidae | *Drosophila repletoides* | Mined from scaffolds |
| Diptera | Drosophilidae | *Drosophila robusta* | Mined from scaffolds |
| Diptera | Drosophilidae | *Drosophila sechellia* | Public: Annotated |
| Diptera | Drosophilidae | *Drosophila simulans* | Public: Annotated |
| Diptera | Drosophilidae | *Drosophila subobscura* | Public: Annotated |
| Diptera | Muscidae | *Musca domestica* | Public: Annotated |
| Diptera | Phoridae | *Megaselia scalaris* | Public: Annotated |
| Diptera | Psychodidae | *Phlebotomus papatasi* | Public: Annotated |
| Diptera | Sarcophagidae | *Sarcophaga peregrina* | Public: Annotated |
| Diptera | Stratiomyidae | *Hermetia illucens* | Public: Annotated |
| Diptera | Syrphidae | *Cheilosia vulpina* | Public: Non-annotated |
| Diptera | Syrphidae | *Chrysotoxum bicinctum* | Public: Non-annotated |
| Diptera | Syrphidae | *Eristalis arbustorum* | Public: Non-annotated |
| Diptera | Syrphidae | *Eristalis pertinax* | Public: Non-annotated |
| Diptera | Syrphidae | *Eristalis tenax* | Public: Annotated |
| Diptera | Syrphidae | *Melanostoma mellinum* | Public: Non-annotated |
| Diptera | Syrphidae | *Scaeva pyrastri* | Public: Non-annotated |
| Diptera | Syrphidae | *Syritta pipiens* | Public: Non-annotated |
| Diptera | Syrphidae | *Volucella inanis* | Public: Non-annotated |
| Diptera | Syrphidae | *Xanthogramma pedissequum* | Public: Non-annotated |
| Diptera | Tachinidae | *Gymnosoma rotundatum* | Public: Non-annotated |
| Diptera | Tachinidae | *Tachina fera* | Public: Non-annotated |
| Diptera | Tachinidae | *Thecocarcelia acutangulata* | Public: Non-annotated |
| Diptera | Tephritidae | *Bactrocera dorsalis* | Public: Annotated |
| Diptera | Tephritidae | *Bactrocera latifrons* | Public: Annotated |
| Diptera | Tephritidae | *Bactrocera oleae* | Public: Annotated |
| Diptera | Tephritidae | *Bactrocera tryoni* | Public: Annotated |
| Diptera | Tephritidae | *Ceratitis capitata* | Public: Annotated |
| Diptera | Tephritidae | *Zeugodacus cucurbitae* | Public: Annotated |
| Ephemeroptera | Baetidae | *Cloeon dipterum* | Mined from scaffolds |
| Hemiptera | Aleyrodidae | *Bemisia tabaci* | Public: Annotated |
| Hemiptera | Aleyrodidae | *Trialeurodes vaporariorum* | Public: Annotated |
| Hemiptera | Alydidae | *Riptortus pedestris* | Public: Annotated |
| Hemiptera | Aphididae | *Acyrthosiphon pisum* | Public: Annotated |
| Hemiptera | Aphididae | *Aphis craccivora* | Public: Annotated |
| Hemiptera | Aphididae | *Aphis glycines* | Public: Annotated |
| Hemiptera | Aphididae | *Aphis gossypii* | Public: Annotated |
| Hemiptera | Aphididae | *Diuraphis noxia* | Public: Annotated |
| Hemiptera | Aphididae | *Eriosoma lanigerum* | Public: Annotated |
| Hemiptera | Aphididae | *Myzus persicae* | Public: Annotated |
| Hemiptera | Aphididae | *Schizaphis graminum* | Public: Annotated |
| Hemiptera | Aphididae | *Schlechtendalia chinensis* | Public: Annotated |
| Hemiptera | Aphididae | *Sitobion avenae* | Public: Annotated |
| Hemiptera | Aphrophoridae | *Philaenus spumarius* | Public: Annotated |
| Hemiptera | Belostomatidae | *Lethocerus indicus* | Public: Annotated |
| Hemiptera | Cicadellidae | *Empoasca onukii* | Public: Annotated |
| Hemiptera | Cicadellidae | *Homalodisca vitripennis* | Public: Annotated |
| Hemiptera | Cicadidae | *Magicicada septendecim* | Public: Annotated |
| Hemiptera | Cicadidae | *Magicicada septendecula* | Public: Annotated |
| Hemiptera | Delphacidae | *Laodelphax striatellus* | Public: Annotated |
| Hemiptera | Delphacidae | *Nilaparvata lugens* | Public: Annotated |
| Hemiptera | Delphacidae | *Sogatella furcifera* | Public: Annotated |
| Hemiptera | Liviidae | *Diaphorina citri* | Public: Annotated |
| Hemiptera | Miridae | *Apolygus lucorum* | Public: Annotated |
| Hemiptera | Miridae | *Nesidiocoris tenuis* | Public: Annotated |
| Hemiptera | Pentatomidae | *Halyomorpha halys* | Public: Annotated |
| Hemiptera | Reduviidae | *Rhodnius prolixus* | Public: Annotated |
| Hemiptera | Reduviidae | *Triatoma infestans* | Public: Annotated |
| Hymenoptera | Apidae | *Apis cerana* | Public: Annotated |
| Hymenoptera | Apidae | *Apis dorsata* | Public: Annotated |
| Hymenoptera | Apidae | *Apis florea* | Public: Annotated |
| Hymenoptera | Apidae | *Apis laboriosa* | Public: Annotated |
| Hymenoptera | Apidae | *Apis mellifera* | Public: Annotated |
| Hymenoptera | Apidae | *Bombus waltoni* | Public: Annotated |
| Hymenoptera | Apidae | *Ceratina australensis* | Mined from scaffolds |
| Hymenoptera | Apidae | *Nomada fabriciana* | Public: Non-annotated |
| Hymenoptera | Braconidae | *Aphidius gifuensis* | Public: Annotated |
| Hymenoptera | Braconidae | *Asobara japonica* | Public: Annotated |
| Hymenoptera | Braconidae | *Cotesia vestalis* | Public: Annotated |
| Hymenoptera | Braconidae | *Diachasma alloeum* | Mined from scaffolds |
| Hymenoptera | Cephidae | *Cephus cinctus* | Public: Annotated |
| Hymenoptera | Formicidae | *Eciton burchellii* | Mined from scaffolds |
| Hymenoptera | Formicidae | *Formica selysi* | Public: Annotated |
| Hymenoptera | Formicidae | *Linepithema humile* | Public: Annotated |
| Hymenoptera | Formicidae | *Monomorium pharaonis* | Public: Non-annotated |
| Hymenoptera | Formicidae | *Ooceraea biroi* | Public: Annotated |
| Hymenoptera | Formicidae | *Pseudomyrmex cubaensis* | Mined from scaffolds |
| Hymenoptera | Formicidae | *Pseudomyrmex elongatus* | Mined from scaffolds |
| Hymenoptera | Formicidae | *Pseudomyrmex flavicornis* | Mined from scaffolds |
| Hymenoptera | Formicidae | *Vollenhovia emeryi* | Public: Annotated |
| Hymenoptera | Formicidae | *Wasmannia auropunctata* | Public: Annotated |
| Hymenoptera | Halictidae | *Lasioglossum albipes* | Mined from scaffolds |
| Hymenoptera | Halictidae | *Lasioglossum morio* | Public: Non-annotated |
| Hymenoptera | Ichneumonidae | *Diadegma semiclausum* | Public: Annotated |
| Hymenoptera | Ichneumonidae | *Ichneumon xanthorius* | Public: Non-annotated |
| Hymenoptera | Melittidae | *Macropis europaea* | Public: Non-annotated |
| Hymenoptera | Pompilidae | *Anoplius nigerrimus* | Public: Non-annotated |
| Hymenoptera | Psenidae | *Mimumesa dahlbomi* | Public: Non-annotated |
| Hymenoptera | Pteromalidae | *Pteromalus puparum* | Public: Annotated |
| Hymenoptera | Tenthredinidae | *Athalia rosae* | Public: Non-annotated |
| Hymenoptera | Tenthredinidae | *Tenthredo notha* | Public: Non-annotated |
| Hymenoptera | Vespidae | *Ancistrocerus nigricornis* | Public: Non-annotated |
| Hymenoptera | Vespidae | *Dolichovespula media* | Public: Non-annotated |
| Hymenoptera | Vespidae | *Dolichovespula saxonica* | Public: Non-annotated |
| Hymenoptera | Vespidae | *Vespa mandarinia* | Public: Annotated |
| Hymenoptera | Vespidae | *Vespa velutina* | Public: Annotated |
| Hymenoptera | Vespidae | *Vespula germanica* | Public: Non-annotated |
| Hymenoptera | Vespidae | *Vespula pensylvanica* | Mined from scaffolds |
| Hymenoptera | Vespidae | *Vespula vulgaris* | Public: Non-annotated |
| Lepidoptera | Blastobasidae | *Blastobasis adustella* | Public: Non-annotated |
| Lepidoptera | Blastobasidae | *Blastobasis lacticolella* | Public: Non-annotated |
| Lepidoptera | Bombycidae | *Bombyx huttoni* | Public: Annotated |
| Lepidoptera | Bombycidae | *Bombyx mandarina* | Public: Annotated |
| Lepidoptera | Bombycidae | *Bombyx mori* | Public: Annotated |
| Lepidoptera | Carposinidae | *Carposina sasakii* | Public: Annotated |
| Lepidoptera | Cossidae | *Zeuzera pyrina* | Public: Non-annotated |
| Lepidoptera | Crambidae | *Chilo suppressalis* | Public: Annotated |
| Lepidoptera | Crambidae | *Cnaphalocrocis medinalis* | Public: Annotated |
| Lepidoptera | Crambidae | *Diatraea saccharalis* | Public: Annotated |
| Lepidoptera | Crambidae | *Ostrinia furnacalis* | Public: Annotated |
| Lepidoptera | Crambidae | *Ostrinia nubilalis* | Public: Annotated |
| Lepidoptera | Crambidae | *Parapoynx stratiotata* | Public: Non-annotated |
| Lepidoptera | Crambidae | *Scirpophaga incertulas* | Public: Annotated |
| Lepidoptera | Erebidae | *Arctia plantaginis* | Public: Annotated |
| Lepidoptera | Erebidae | *Eilema depressum* | Public: Non-annotated |
| Lepidoptera | Erebidae | *Eilema sororculum* | Public: Non-annotated |
| Lepidoptera | Erebidae | *Euproctis similis* | Public: Annotated |
| Lepidoptera | Erebidae | *Hypena proboscidalis* | Public: Non-annotated |
| Lepidoptera | Erebidae | *Hyphantria cunea* | Public: Annotated |
| Lepidoptera | Erebidae | *Laspeyria flexula* | Public: Non-annotated |
| Lepidoptera | Erebidae | *Lymantria dispar* | Public: Annotated |
| Lepidoptera | Erebidae | *Lymantria monacha* | Public: Non-annotated |
| Lepidoptera | Erebidae | *Orgyia antiqua* | Public: Non-annotated |
| Lepidoptera | Erebidae | *Schrankia costaestrigalis* | Public: Non-annotated |
| Lepidoptera | Erebidae | *Spilarctia lutea* | Public: Non-annotated |
| Lepidoptera | Erebidae | *Spilosoma lubricipeda* | Public: Annotated |
| Lepidoptera | Gelechiidae | *Tuta absoluta* | Public: Annotated |
| Lepidoptera | Geometridae | *Campaea margaritaria* | Public: Non-annotated |
| Lepidoptera | Geometridae | *Crocallis elinguaria* | Public: Non-annotated |
| Lepidoptera | Geometridae | *Ectropis grisescens* | Public: Annotated |
| Lepidoptera | Geometridae | *Hydriomena furcata* | Public: Non-annotated |
| Lepidoptera | Geometridae | *Idaea aversata* | Public: Non-annotated |
| Lepidoptera | Geometridae | *Operophtera brumata* | Public: Annotated |
| Lepidoptera | Geometridae | *Peribatodes rhomboidaria* | Public: Non-annotated |
| Lepidoptera | Hesperiidae | *Anisynta dominula* | Mined from scaffolds |
| Lepidoptera | Hesperiidae | *Apostictopterus fuliginosus* | Public: Annotated |
| Lepidoptera | Hesperiidae | *Apyrrothrix aesculapus* | Mined from scaffolds |
| Lepidoptera | Hesperiidae | *Apyrrothrix chalybea* | Mined from scaffolds |
| Lepidoptera | Hesperiidae | *Aspitha aspitha* | Mined from scaffolds |
| Lepidoptera | Hesperiidae | *Astictopterus jama* | Public: Annotated |
| Lepidoptera | Hesperiidae | *Atarnes sallei* | Mined from scaffolds |
| Lepidoptera | Hesperiidae | *Atrytonopsis hianna* | Mined from scaffolds |
| Lepidoptera | Hesperiidae | *Azonax typhaon* | Mined from scaffolds |
| Lepidoptera | Hesperiidae | *Barca bicolor* | Public: Annotated |
| Lepidoptera | Hesperiidae | *Bibasis iluska* | Mined from scaffolds |
| Lepidoptera | Hesperiidae | *Bolla brennus* | Mined from scaffolds |
| Lepidoptera | Hesperiidae | *Camptopleura auxo* | Mined from scaffolds |
| Lepidoptera | Hesperiidae | *Carystus phorcus* | Mined from scaffolds |
| Lepidoptera | Hesperiidae | *Cecropterus lyciades* | Public: Non-annotated |
| Lepidoptera | Hesperiidae | *Cecropterus tehuacana* | Mined from scaffolds |
| Lepidoptera | Hesperiidae | *Cecropterus toxeus* | Mined from scaffolds |
| Lepidoptera | Hesperiidae | *Chamunda chamunda* | Mined from scaffolds |
| Lepidoptera | Hesperiidae | *Choaspes benjaminii* | Public: Annotated |
| Lepidoptera | Hesperiidae | *Codatractus alcaeus* | Mined from scaffolds |
| Lepidoptera | Hesperiidae | *Cupitha purreea* | Mined from scaffolds |
| Lepidoptera | Hesperiidae | *Cycloglypha thrasibulus* | Mined from scaffolds |
| Lepidoptera | Hesperiidae | *Dalla cyprius* | Mined from scaffolds |
| Lepidoptera | Hesperiidae | *Ebrietas anacreon* | Mined from scaffolds |
| Lepidoptera | Hesperiidae | *Eburuncus unifasciata* | Mined from scaffolds |
| Lepidoptera | Hesperiidae | *Ernsta confusa* | Mined from scaffolds |
| Lepidoptera | Hesperiidae | *Ernsta delagoae* | Mined from scaffolds |
| Lepidoptera | Hesperiidae | *Erynnis brizo* | Mined from scaffolds |
| Lepidoptera | Hesperiidae | *Erynnis tages* | Public: Non-annotated |
| Lepidoptera | Hesperiidae | *Euphyes dion* | Mined from scaffolds |
| Lepidoptera | Hesperiidae | *Euschemon rafflesia* | Public: Annotated |
| Lepidoptera | Hesperiidae | *Gindanes brontinus* | Mined from scaffolds |
| Lepidoptera | Hesperiidae | *Hasora badra* | Public: Annotated |
| Lepidoptera | Hesperiidae | *Hesperia comma* | Public: Non-annotated |
| Lepidoptera | Hesperiidae | *Jera tricuspidata* | Mined from scaffolds |
| Lepidoptera | Hesperiidae | *Jonaspyge jonas* | Mined from scaffolds |
| Lepidoptera | Hesperiidae | *Lerema accius* | Public: Annotated |
| Lepidoptera | Hesperiidae | *Metardaris cosinga* | Mined from scaffolds |
| Lepidoptera | Hesperiidae | *Microceris merops* | Mined from scaffolds |
| Lepidoptera | Hesperiidae | *Microceris scylla* | Mined from scaffolds |
| Lepidoptera | Hesperiidae | *Mimoniades versicolor* | Mined from scaffolds |
| Lepidoptera | Hesperiidae | *Muschampia proto* | Mined from scaffolds |
| Lepidoptera | Hesperiidae | *Mysoria galgala* | Mined from scaffolds |
| Lepidoptera | Hesperiidae | *Mysoria sejanus* | Mined from scaffolds |
| Lepidoptera | Hesperiidae | *Noctuana haematospila* | Mined from scaffolds |
| Lepidoptera | Hesperiidae | *Ochlodes sylvanus* | Public: Non-annotated |
| Lepidoptera | Hesperiidae | *Ouleus salvina* | Mined from scaffolds |
| Lepidoptera | Hesperiidae | *Oxynetra semihyalina* | Mined from scaffolds |
| Lepidoptera | Hesperiidae | *Parelbella macleannani* | Mined from scaffolds |
| Lepidoptera | Hesperiidae | *Parnara guttata* | Public: Annotated |
| Lepidoptera | Hesperiidae | *Passova nigrocephala* | Mined from scaffolds |
| Lepidoptera | Hesperiidae | *Polygonus leo* | Mined from scaffolds |
| Lepidoptera | Hesperiidae | *Pseudosarbia flavofasciata* | Mined from scaffolds |
| Lepidoptera | Hesperiidae | *Pyrgus malvae* | Public: Non-annotated |
| Lepidoptera | Hesperiidae | *Pyrrhopyge papius* | Mined from scaffolds |
| Lepidoptera | Hesperiidae | *Pyrrhopyge schausi* | Mined from scaffolds |
| Lepidoptera | Hesperiidae | *Pyrrhopyge sergius* | Mined from scaffolds |
| Lepidoptera | Hesperiidae | *Pyrrhopyge tatei* | Mined from scaffolds |
| Lepidoptera | Hesperiidae | *Pythonides amaryllis* | Mined from scaffolds |
| Lepidoptera | Hesperiidae | *Salantoia eriopis* | Mined from scaffolds |
| Lepidoptera | Hesperiidae | *Spialia spio* | Mined from scaffolds |
| Lepidoptera | Hesperiidae | *Spioniades abbreviata* | Mined from scaffolds |
| Lepidoptera | Hesperiidae | *Tagiades litigiosa* | Public: Annotated |
| Lepidoptera | Hesperiidae | *Thymelicus lineola* | Mined from scaffolds |
| Lepidoptera | Hesperiidae | *Thymelicus sylvestris* | Public: Non-annotated |
| Lepidoptera | Hesperiidae | *Udranomia eurus* | Mined from scaffolds |
| Lepidoptera | Hesperiidae | *Urbanus proteus* | Mined from scaffolds |
| Lepidoptera | Hesperiidae | *Yanguna spatiosa* | Mined from scaffolds |
| Lepidoptera | Hesperiidae | *Zonia zonia* | Mined from scaffolds |
| Lepidoptera | Lasiocampidae | *Dendrolimus kikuchii* | Public: Annotated |
| Lepidoptera | Lasiocampidae | *Dendrolimus punctatus* | Public: Annotated |
| Lepidoptera | Lycaenidae | *Aricia agestis* | Public: Non-annotated |
| Lepidoptera | Lycaenidae | *Curetis bulis* | Public: Annotated |
| Lepidoptera | Lycaenidae | *Cyaniris semiargus* | Public: Non-annotated |
| Lepidoptera | Lycaenidae | *Cyclargus thomasi* | Mined from scaffolds |
| Lepidoptera | Lycaenidae | *Glaucopsyche alexis* | Public: Non-annotated |
| Lepidoptera | Lycaenidae | *Lycaena phlaeas* | Public: Annotated |
| Lepidoptera | Lycaenidae | *Lysandra coridon* | Public: Non-annotated |
| Lepidoptera | Lycaenidae | *Plebejus argus* | Public: Non-annotated |
| Lepidoptera | Noctuidae | *Agrochola circellaris* | Public: Non-annotated |
| Lepidoptera | Noctuidae | *Agrotis ipsilon* | Public: Annotated |
| Lepidoptera | Noctuidae | *Amphipyra berbera* | Public: Non-annotated |
| Lepidoptera | Noctuidae | *Amphipyra tragopoginis* | Public: Non-annotated |
| Lepidoptera | Noctuidae | *Autographa gamma* | Public: Non-annotated |
| Lepidoptera | Noctuidae | *Autographa pulchrina* | Public: Non-annotated |
| Lepidoptera | Noctuidae | *Craniophora ligustri* | Public: Non-annotated |
| Lepidoptera | Noctuidae | *Eupsilia transversa* | Public: Non-annotated |
| Lepidoptera | Noctuidae | *Griposia aprilina* | Public: Non-annotated |
| Lepidoptera | Noctuidae | *Helicoverpa armigera* | Public: Annotated |
| Lepidoptera | Noctuidae | *Helicoverpa zea* | Public: Annotated |
| Lepidoptera | Noctuidae | *Hydraecia micacea* | Public: Non-annotated |
| Lepidoptera | Noctuidae | *Mamestra brassicae* | Public: Non-annotated |
| Lepidoptera | Noctuidae | *Mamestra configurata* | Public: Annotated |
| Lepidoptera | Noctuidae | *Mesoligia furuncula* | Public: Non-annotated |
| Lepidoptera | Noctuidae | *Mythimna ferrago* | Public: Non-annotated |
| Lepidoptera | Noctuidae | *Mythimna impura* | Public: Non-annotated |
| Lepidoptera | Noctuidae | *Mythimna separata* | Public: Annotated |
| Lepidoptera | Noctuidae | *Noctua janthe* | Public: Non-annotated |
| Lepidoptera | Noctuidae | *Ochropleura plecta* | Public: Non-annotated |
| Lepidoptera | Noctuidae | *Omphaloscelis lunosa* | Public: Non-annotated |
| Lepidoptera | Noctuidae | *Phlogophora meticulosa* | Public: Non-annotated |
| Lepidoptera | Noctuidae | *Sesamia nonagrioides* | Public: Non-annotated |
| Lepidoptera | Noctuidae | *Spodoptera exigua* | Public: Annotated |
| Lepidoptera | Noctuidae | *Spodoptera frugiperda* | Public: Annotated |
| Lepidoptera | Noctuidae | *Spodoptera litura* | Public: Annotated |
| Lepidoptera | Noctuidae | *Trichoplusia ni* | Public: Non-annotated |
| Lepidoptera | Noctuidae | *Xestia c-nigrum* | Public: Non-annotated |
| Lepidoptera | Noctuidae | *Xestia xanthographa* | Public: Non-annotated |
| Lepidoptera | Notodontidae | *Clostera curtula* | Public: Non-annotated |
| Lepidoptera | Notodontidae | *Furcula furcula* | Public: Non-annotated |
| Lepidoptera | Notodontidae | *Notodonta dromedarius* | Public: Non-annotated |
| Lepidoptera | Notodontidae | *Phalera bucephala* | Public: Non-annotated |
| Lepidoptera | Notodontidae | *Pheosia gnoma* | Public: Non-annotated |
| Lepidoptera | Notodontidae | *Pheosia tremula* | Public: Non-annotated |
| Lepidoptera | Notodontidae | *Ptilodon capucinus* | Public: Non-annotated |
| Lepidoptera | Notodontidae | *Thaumetopoea pityocampa* | Public: Annotated |
| Lepidoptera | Nymphalidae | *Danaus chrysippus* | Public: Annotated |
| Lepidoptera | Nymphalidae | *Danaus melanippus* | Mined from scaffolds |
| Lepidoptera | Nymphalidae | *Danaus plexippus* | Public: Annotated |
| Lepidoptera | Nymphalidae | *Dryas iulia* | Mined from scaffolds |
| Lepidoptera | Nymphalidae | *Elymnias hypermnestra* | Public: Annotated |
| Lepidoptera | Nymphalidae | *Erebia ligea* | Public: Non-annotated |
| Lepidoptera | Nymphalidae | *Heliconius clysonymus* | Public: Annotated |
| Lepidoptera | Nymphalidae | *Heliconius cydno* | Public: Annotated |
| Lepidoptera | Nymphalidae | *Heliconius elevatus* | Mined from scaffolds |
| Lepidoptera | Nymphalidae | *Heliconius ethilla* | Mined from scaffolds |
| Lepidoptera | Nymphalidae | *Heliconius hecale* | Public: Annotated |
| Lepidoptera | Nymphalidae | *Heliconius heurippa* | Mined from scaffolds |
| Lepidoptera | Nymphalidae | *Heliconius ismenius* | Public: Annotated |
| Lepidoptera | Nymphalidae | *Heliconius pachinus* | Public: Annotated |
| Lepidoptera | Nymphalidae | *Heliconius pardalinus* | Mined from scaffolds |
| Lepidoptera | Nymphalidae | *Heliconius timareta* | Mined from scaffolds |
| Lepidoptera | Nymphalidae | *Junonia coenia* | Public: Annotated |
| Lepidoptera | Nymphalidae | *Junonia litoralis* | Public: Annotated |
| Lepidoptera | Nymphalidae | *Junonia vestina* | Public: Annotated |
| Lepidoptera | Nymphalidae | *Limenitis arthemis* | Public: Annotated |
| Lepidoptera | Nymphalidae | *Maniola jurtina* | Public: Non-annotated |
| Lepidoptera | Nymphalidae | *Melitaea cinxia* | Public: Non-annotated |
| Lepidoptera | Nymphalidae | *Mellicta athalia* | Public: Non-annotated |
| Lepidoptera | Nymphalidae | *Nymphalis io* | Public: Non-annotated |
| Lepidoptera | Nymphalidae | *Pararge aegeria* | Public: Non-annotated |
| Lepidoptera | Nymphalidae | *Vanessa atalanta* | Public: Non-annotated |
| Lepidoptera | Nymphalidae | *Vanessa tameamea* | Mined from scaffolds |
| Lepidoptera | Papilionidae | *Atrophaneura alcinous* | Public: Annotated |
| Lepidoptera | Papilionidae | *Euryades corethrus* | Public: Annotated |
| Lepidoptera | Papilionidae | *Lamproptera meges* | Public: Annotated |
| Lepidoptera | Papilionidae | *Losaria neptunus* | Public: Annotated |
| Lepidoptera | Papilionidae | *Luehdorfia chinensis* | Public: Annotated |
| Lepidoptera | Papilionidae | *Mimoides lysithous* | Public: Annotated |
| Lepidoptera | Papilionidae | *Ornithoptera priamus* | Public: Annotated |
| Lepidoptera | Papilionidae | *Ornithoptera richmondia* | Public: Annotated |
| Lepidoptera | Papilionidae | *Papilio bianor* | Public: Annotated |
| Lepidoptera | Papilionidae | *Papilio dardanus* | Public: Annotated |
| Lepidoptera | Papilionidae | *Papilio machaon* | Public: Annotated |
| Lepidoptera | Papilionidae | *Papilio memnon* | Public: Annotated |
| Lepidoptera | Papilionidae | *Papilio polytes* | Public: Annotated |
| Lepidoptera | Papilionidae | *Papilio protenor* | Public: Annotated |
| Lepidoptera | Papilionidae | *Papilio slateri* | Public: Annotated |
| Lepidoptera | Papilionidae | *Papilio xuthus* | Public: Annotated |
| Lepidoptera | Papilionidae | *Parnassius apollo* | Public: Annotated |
| Lepidoptera | Papilionidae | *Protesilaus protesilaus* | Mined from scaffolds |
| Lepidoptera | Papilionidae | *Teinopalpus imperialis* | Public: Annotated |
| Lepidoptera | Papilionidae | *Trogonoptera brookiana* | Public: Annotated |
| Lepidoptera | Pieridae | *Aporia crataegi* | Public: Annotated |
| Lepidoptera | Pieridae | *Delias pasithoe* | Public: Annotated |
| Lepidoptera | Pieridae | *Pieris brassicae* | Public: Non-annotated |
| Lepidoptera | Pieridae | *Pieris napi* | Public: Annotated |
| Lepidoptera | Pieridae | *Pieris rapae* | Public: Annotated |
| Lepidoptera | Plutellidae | *Plutella xylostella* | Public: Annotated |
| Lepidoptera | Pterophoridae | *Emmelina monodactyla* | Public: Non-annotated |
| Lepidoptera | Pyralidae | *Amyelois transitella* | Public: Annotated |
| Lepidoptera | Pyralidae | *Endotricha flammealis* | Public: Non-annotated |
| Lepidoptera | Pyralidae | *Ephestia elutella* | Public: Annotated |
| Lepidoptera | Pyralidae | *Galleria mellonella* | Public: Annotated |
| Lepidoptera | Pyralidae | *Plodia interpunctella* | Public: Annotated |
| Lepidoptera | Riodinidae | *Apodemia mormo* | Public: Annotated |
| Lepidoptera | Riodinidae | *Apodemia multiplaga* | Mined from scaffolds |
| Lepidoptera | Riodinidae | *Apodemia murphyi* | Mined from scaffolds |
| Lepidoptera | Riodinidae | *Emesis heteroclita* | Mined from scaffolds |
| Lepidoptera | Riodinidae | *Emesis mandana* | Mined from scaffolds |
| Lepidoptera | Riodinidae | *Emesis tenedia* | Mined from scaffolds |
| Lepidoptera | Riodinidae | *Eurybia lycisca* | Mined from scaffolds |
| Lepidoptera | Riodinidae | *Hypophylla argenissa* | Mined from scaffolds |
| Lepidoptera | Riodinidae | *Stalachtis halloweeni* | Mined from scaffolds |
| Lepidoptera | Saturniidae | *Actias luna* | Public: Annotated |
| Lepidoptera | Saturniidae | *Samia ricini* | Public: Annotated |
| Lepidoptera | Sesiidae | *Sesia apiformis* | Public: Non-annotated |
| Lepidoptera | Sesiidae | *Synanthedon vespiformis* | Public: Non-annotated |
| Lepidoptera | Sphingidae | *Deilephila porcellus* | Public: Non-annotated |
| Lepidoptera | Sphingidae | *Hemaris fuciformis* | Public: Non-annotated |
| Lepidoptera | Sphingidae | *Hyles vespertilio* | Public: Non-annotated |
| Lepidoptera | Sphingidae | *Manduca sexta* | Public: Annotated |
| Lepidoptera | Sphingidae | *Mimas tiliae* | Public: Non-annotated |
| Lepidoptera | Tineidae | *Tinea semifulvella* | Public: Non-annotated |
| Lepidoptera | Tortricidae | *Adoxophyes honmai* | Public: Annotated |
| Lepidoptera | Tortricidae | *Cydia pomonella* | Public: Annotated |
| Lepidoptera | Tortricidae | *Cydia splendana* | Public: Non-annotated |
| Lepidoptera | Tortricidae | *Hedya salicella* | Mined from scaffolds |
| Lepidoptera | Tortricidae | *Notocelia uddmanniana* | Public: Non-annotated |
| Lepidoptera | Tortricidae | *Pammene fasciana* | Public: Non-annotated |
| Lepidoptera | Ypsolophidae | *Ypsolopha scabrella* | Public: Non-annotated |
| Lepidoptera | Zygaenidae | *Zygaena filipendulae* | Public: Non-annotated |
| Neuroptera | Chrysopidae | *Chrysopa pallens* | Public: Annotated |
| Odonata | Libellulidae | *Pantala flavescens* | Public: Annotated |
| Orthoptera | Acrididae | *Locusta migratoria* | Public: Annotated |
| Orthoptera | Gryllidae | *Gryllus bimaculatus* | Public: Annotated |
| Orthoptera | Gryllidae | *Teleogryllus occipitalis* | Public: Annotated |
| Orthoptera | Trigonidiidae | *Apteronemobius asahinai* | Mined from scaffolds |
| Siphonaptera | Pulicidae | *Ctenocephalides felis* | Public: Annotated |
| Strepsiptera | Mengenillidae | *Mengenilla moldrzyki* | Public: Annotated |
| Trichoptera | Limnephilidae | *Limnephilus lunatus* | Public: Non-annotated |
| Trichoptera | Stenopsychidae | *Stenopsyche tienmushanensis* | Public: Annotated |
