# Supplementary material for: Interrogating 1000 insect genomes for NUMTs: A risk assessment for estimates of species richness
Source: PLoS One. 2023 Jun 8;18(6):e0286620. doi: 10.1371/journal.pone.0286620 (PMC10249859; doi:10.1371/journal.pone.0286620)
Supplement: S1 File — (DOCX) [file pone.0286620.s021.docx]

**Supplementary Materials**

**Nuclear genome sizes**

When available, an assembly length was obtained for each of the 1,446 species using the assembly-stats option of ‘ncbi-genome-download’. Pfenninger *et al*. [1] have shown that 10x assemblies accurately estimate genome size, and our examination of the 1,429 species with a coverage estimate showed they fell into two categories **(S2 Fig**). Among the 538 assemblies with < 5x coverage, 99% were at a contig level, while 88% of the 891 assemblies with > 5x coverage were at a scaffold or chromosome level. Assembly length was not correlated with sequence coverage (*r* = -0.07; *P* = 0.07) for species in the high category as expected if it indicates genome size rather than sequence volume. By contrast, assembly length (*r* = 0.97; *P* < 4.4 x 10^-16^) was strongly correlated with coverage for species in the low category as expected if it simply reflects data volume. Fifteen genomes annotated as high coverage insect genomes possessed a very low assembly length (**S2 Fig**). As inspection (i.e., the associated “Comments” section) indicated they were endosymbiont bacteria (e.g. *Rickettsia*), they were removed from the dataset. This analysis suggested that the length of assemblies based on >5x coverage is an accurate proxy for genome size. We tested this conclusion by comparing genome size estimates for 5x assemblies with those obtained by flow cytophotometry.

On 1/12/21, the animal genome size database [2] possessed cytophotometric estimates for 148 of the 891 high coverage species, and these values were converted from picograms to megabases (1 pg = 978 Mb; [3]). When several cytophotometric estimates were available for a species, they were averaged. We then examined the correlation between these independent measures of genome size using the *cor.test* function in R. Because some genera (e.g. *Drosophila*) were heavily represented, the strength of the correlation was reassessed using one species per genus. Assembly length for high coverage genomes was positively correlated with genome size estimates determined by flow cytometry for all species (*r* = 0.83; *P* < 4.4 x 10^-16^) and for one representative per genus (*r* = 0.78; *P* < 9.4 x 10^-16^) (**S4 Fig**).

**Identification of new mitogenomes**

Unrecognized mitogenomes can occur within nuclear genome assemblies. To identify such cases, we first isolated scaffolds/contigs ranging in size from 14–21 kb for each of the 1,446 species. We employed this size range because it spanned the lengths (14.4–20.9 kb) of the annotated NCBI mitogenomes represented in our dataset and previous reports on mitogenome size [4]. By only retaining 14–21 kb scaffolds, we excluded large NUMTs that were assembled with their flanking nuclear genome regions.

Using the subset of 14–21 kb scaffolds, we performed BLASTn searches with COI barcodes derived from our BOLD-derived COI barcode dataset. When possible, we employed a COI sequence from the same family as the target species. If unavailable, we used sequential BLASTn searches with five COI sequences, employing one representative from each of the five major insect orders: Coleoptera (Curculionidae), Diptera (Drosophilidae), Hemiptera (Aphididae), Hymenoptera (Braconidae), and Lepidoptera (Geometridae). We retained scaffolds from all species for which COI barcode hits > 600 bp were detected in one or, at most, two scaffolds. In all, nuclear assemblies for 423 species contained scaffolds meeting these criteria, including 398 for which a single candidate mitogenome scaffold was found, and 25 for which two candidate mitogenome scaffolds were found. Following annotation using MITOS and filtering (detailed below), we retained 251 mitogenomes including 108 that were new (**Table S6**).

**Mitogenome filtering and annotation**

Among the 404 mitogenomes on NCBI for species examined in this study, just 219 were annotated. We filtered these mitogenomes, keeping 215 that contained all 13 protein-coding genes (PCGs) expected for animal mitochondrial genomes [5], and those that listed these genes in the expected order. To confirm the correct gene order, we recorded the identity of the first PCG listed in each GenBank-formatted mitogenome file. Considering the identity of this first gene, we verified that the last PCG is as expected, given knowledge of gene order in the ancestral insect mitogenome [4], and the circularity of this genome. We next processed the 185 NCBI mitogenomes that lacked an annotation as well as all 423 candidate mitogenomes identified through our search of the nuclear genome assemblies. These sequences were submitted for *de novo* annotation on MITOS and the resulting BED files were used to verify that each mitogenome contained only one copy of COI and at least 13 PCGs. While only 13 PCGs are present in animal mitochondrial genomes [5], more can be designated by annotation software due to fragmented gene predictions [6]. We next filtered mitogenomes to retain assemblies with the expected gene order, as described above. Among assemblies passing these filters, we excluded eight mitogenomes derived from NCBI (i.e. from those that lacked a public annotation) whose length was > 20.9 kb (21,636–42,543 bp; mean = 28,472 bp). These filtering steps produced 117 filtered mitogenomes from those available on NCBI but lacking a public annotation, and 108 new filtered mitogenomes identified from nuclear assemblies.

**References**

1. Pfenninger M, Schonnenbeck P, Schell T. ModEst – Accurate estimation of genome size from NGS data. Mol. Ecol. Res. 2022; 22: 1454-1464.

2. Gregory TR. Animal Genome Size Database. 2021; http://www.genomesize.com.

3. Dolezel J, Bartos J, Voglmayr H, Greilhuber J. Nuclear DNA content and genome size of trout and human. Cytometry A. 2003; 51: 127–128.

4. Cameron SL. Insect mitochondrial genomics: implications for evolution and phylogeny. Annu. Rev. Entomol., 2014; 59: 95–117.

5. Boore JL. Animal mitochondrial genomes. Nucleic Acids Res. 1999; 27: 1767–1780.

6. Bernt M, Donath A, Jühling F, Externbrink F, Florentz C, Fritzsch G, et al. MITOS: improved de novo metazoan mitochondrial genome annotation. Mol. Phylogenet. Evol. 2013; 69: 313–319.
